# Impact of duplicate gene copies on phylogenetic analysis and divergence time estimates in butterflies

**DOI:** 10.1186/1471-2148-9-99

**Published:** 2009-05-13

**Authors:** Nélida Pohl, Marilou P Sison-Mangus, Emily N Yee, Saif W Liswi, Adriana D Briscoe

**Affiliations:** 1 Department of Ecology and Evolutionary Biology, University of California, Irvine, Irvine, CA 92697, USA

## Abstract

**Background:**

The increase in availability of genomic sequences for a wide range of organisms has revealed gene duplication to be a relatively common event. Encounters with duplicate gene copies have consequently become almost inevitable in the context of collecting gene sequences for inferring species trees. Here we examine the effect of incorporating duplicate gene copies evolving at different rates on tree reconstruction and time estimation of recent and deep divergences in butterflies.

**Results:**

Sequences from ultraviolet-sensitive (*UVRh*), blue-sensitive (*BRh*), and long-wavelength sensitive (*LWRh*) opsins,*EF-1α *and *COI *were obtained from 27 taxa representing the five major butterfly families (5535 bp total). Both *BRh *and *LWRh *are present in multiple copies in some butterfly lineages and the different copies evolve at different rates. Regardless of the phylogenetic reconstruction method used, we found that analyses of combined data sets using either slower or faster evolving copies of duplicate genes resulted in a single topology in agreement with our current understanding of butterfly family relationships based on morphology and molecules. Interestingly, individual analyses of *BRh *and *LWRh *sequences also recovered these family-level relationships. Two different relaxed clock methods resulted in similar divergence time estimates at the shallower nodes in the tree, regardless of whether faster or slower evolving copies were used, with larger discrepancies observed at deeper nodes in the phylogeny. The time of divergence between the monarch butterfly *Danaus plexippus *and the queen *D. gilippus *(15.3–35.6 Mya) was found to be much older than the time of divergence between monarch co-mimic *Limenitis archippus *and red-spotted purple *L. arthemis *(4.7–13.6 Mya), and overlapping with the time of divergence of the co-mimetic passionflower butterflies *Heliconius erato *and *H. melpomene *(13.5–26.1 Mya). Our family-level results are congruent with recent estimates found in the literature and indicate an age of 84–113 million years for the divergence of all butterfly families.

**Conclusion:**

These results are consistent with diversification of the butterfly families following the radiation of angiosperms and suggest that some classes of opsin genes may be usefully employed for both phylogenetic reconstruction and divergence time estimation.

## Background

Gene duplication has long been recognized as a major source of evolutionary innovation [1]. It is a pervasive evolutionary process, with 50% of all genes in any given genome expected to duplicate and proliferate at least once in time scales ranging from 35 to 350 MA [2]. In molecular phylogenetics, gene duplication is a process that can lead to discordance between gene and species trees, and previous work has shown large problems with duplicate genes undergoing concerted evolution or birth-and-death processes [3]. The consensus has been to avoid the use of paralogous genes until methods are developed to handle their potential confounding effects [4]. Given the high likelihood of gene duplication under neutral evolutionary processes, however, as the size of molecular data sets gets larger (in number of genes and taxa used) the amplification of duplicated genes may happen inadvertently even in the course of targeting genes that appear at first glance to be single copy (See below). The challenge is to begin studying the range of phylogenetic signal such duplicate genes provide, to assess their evolutionary dynamics and potential signal for phylogenetics. Within the butterflies, for instance, duplicate copies of opsin genes have been found both within and between families [5], and our previous work suggested the possible utility of one of the opsins for phylogenetic purposes[6]. The current work seeks to clarify the potential utility of an expanded collection of opsins for butterfly phylogenetic reconstruction and divergence time estimation.

Historically opsin genes have been advocated as phylogenetic markers, due to the amount of information we possess about their molecular evolution relative to other nuclear genes, and the wealth of cloned sequences available for a wide array of organisms [7]. In fact, the long wavelength-sensitive opsin gene (*LWRh*) has routinely been used for the past 10 years in bee, bumblebee and wasp phylogenetic studies [8-12], and has proven useful at both shallow [13] and deep phylogenetic levels, suggesting its utility at resolving family level, Cretaceous age insect divergences [14,15]. After gaining momentum as a phylogenetic marker, a second copy of the gene, *LWRh2*, was subsequently discovered in bees, which fortunately appears to have evolved under a trajectory independent of the first copy, making both copies suitable for tree building [16]. Currently there is little information about the potential use of other opsin genes in reconstructing insect phylogenies [16], yet almost all insects that have been studied including butterflies have three clades of opsins that encode spectrally distinct visual pigments present in the adult compound eye that are ultraviolet- (*UVRh*), blue- (*BRh*) and long wavelength (*LWRh*)-sensitive [17]. This suggests that other clades of opsins may also be useful for phylogenetic reconstruction over a similar range of divergence times.

Butterflies are some of the best known organisms, possessing remarkable life histories and an uncanny beauty, but yet their most basal relationships have been until recently still obscure [18]. In the early days of butterfly evolution research, the study of the oldest butterfly lineages was intertwined with speculations about timing of their origins [19-22]. In more recent studies, the complexity of simply finding the most plausible topologies, and the difficulty of disentangling molecular evolutionary rates and divergence times, resulted in few studies directly concerned with timing the divergence of butterfly clades. Concerns about the applicability of a molecular clock [23], and the scarcity of butterfly fossils with which to calibrate it [24-26] have also undoubtedly contributed to this paucity in the literature. In recent years the advent of both non-parametric and highly parametric Bayesian [27,28] methods that free the estimations of divergence times from the restrictions of a molecular clock and permit the incorporation of flexible fossil or biogeographical calibration points, has rekindled efforts to date the different diversification events within butterflies, and in the process sparked a controversy about when and where butterflies originated [27,29]. A generally young and scant record comprised of about 50 Rhopaloceran fossils, a group which includes the skippers (Hesperioidea), nocturnal butterflies (Hedyloidea) and true dayflying butterflies (Papilionoidea), all found within the Cenozoic Era (65.5-0 Mya) and not older than 56–57 Ma (a skipper) and 48 Ma (a papilionid), respectively [30,31], has in of itself not been particularly useful in the direct estimation of the earliest butterfly divergences; and is considered by some researchers as a veritable indicator of a recent butterfly origin, dating back to the last epoch of the late Cretaceous (70.6 ± 0.6 – 65.8 ± 0.3 Mya) or early Cenozoic (65.5 – 0.0 Mya) no earlier than 70 Ma ago [18,29]. On the other hand, molecular phylogenetic methods have produced much older divergence time estimates for several butterfly families, prompting many to ascribe the origin of butterflies to the diversification of angiosperms, between 100 and 140 Mya [32-37].

Another group of insects thought to have evolved concordantly with the early diversification of angiosperms are the ants [38], but despite thorough sampling and an ample fossil record, disagreements concerning basal relationships and timing of the earliest divergences still exist [39,40](but see [41]). In contrast, the basal relationships of butterflies are for the most part resolved, with our current understanding of relationships at the familial level being based on the study of Wahlberg and collaborators, which employed both molecular and morphological data to resolve deep nodes in the phylogeny of butterflies [42]. Therefore, with a known phylogeny, butterflies are a useful group of organisms for examining the impact of duplicate genes on phylogenetic reconstruction and divergence time estimation.

In this study, we examine the effect of including duplicated opsin genes evolving at different rates on phylogenetic reconstruction and divergence time estimates. We find that individual and combined analyses of the *BRh *and *LWRh *genes are able to recover butterfly family-level relationships where previously morphological characters were required in addition to molecular [42]. We estimate divergence times for clades of high interest to the ecology and evolutionary biology communities, such as for the co-mimics *Heliconius erato *and *H. melpomene *[43], the migratory monarch *Danaus plexippus *and the non-migratory queen *D. gilippus *[44] and their co-mimics in the genus *Limenitis *[45-47]. We find our estimates of family-level times of divergence with slower evolving gene duplicates to mostly be in agreement with other recent estimates found in the literature based on molecular data, and we push back the minimum age of divergence for the most basal butterfly family to 113 Ma. Our results suggest the potential utility of the opsins for resolving even older and more complex group relationships such as the moths.

## Results and discussion

### Relative rates tests classify duplicate BRh and LWRh opsins functionally

As mentioned previously, most insects including butterflies have adult compound eyes that contain at least three classes of opsin genes (*UVRh, BRh *and *LWRh*) that encode visual pigments with wavelengths of peak absorbance, λ_max_, that fall roughly into the UV (300–400 nm), blue (400–500 nm) and long wavelength (500–600 nm) portions of the light spectrum. Within these broad partitions of the spectrum, the visual pigments of butterflies typically cluster in narrower ranges (i.e., 345–380 nm, 437–470 nm and 514–565) (reviewed in [48]). There are, however, additional opsins that evolved from these three basic classes in some butterfly eyes, which encode visual pigments with λ_max _values outside of these typical ranges (see below).

All three basic classes of opsin (*UVRh, BRh *and *LWRh*) were present in all 27 butterfly species included in this study [Additional File 1], except in the two closely-related satyrines, *Neominois ridingsii *and *Oeneis chryxus*, in which no *BRh *gene could be found after exhaustive screening of head-specific cDNAs. We think that these species probably do have blue-sensitive visual pigments in the eye based on physiological studies (Gary Bernard, pers. comm.) but we were simply unsuccessful in retrieving them. Similarly, we also think that besides the violet opsin we found in the pierid *Colias philodice *this species likely has a blue-sensitive visual pigment in the eye based on electrophysiological studies of a related species [49] but we did not find it. Full-length coding regions were otherwise obtained for the opsin cDNAs, including both start and stop codons. The size of these transcripts, including 3' and 5' UTR regions ranged from 1137–1575 bp (*UVRh*), 996–1590 bp (*BRh*), and 1143–1743 bp (*LWRh*). Besides the three basic opsin classes, all lycaenid butterflies (this study and [50]) and the pierid *Pieris rapae *[51] have duplicated blue opsins, representing two independent *BRh *duplications, while the papilionids *Papilio xuthus *and *P. glaucus*, the riodinid *Apodemia mormo *and the moth *Bombyx mori *possess duplicated *LWRh *opsins [6,52,53] representing four independent gene duplications (gene names for the long-wavelength pigments have been renamed here for simplicity).

Relative rates test showed that of the lycaenid blue opsin duplicates, *BRh1 *evolved slower than *BRh2 *in every one of the 7 lycaenid species sampled, although not significantly so (Table 1). The slower rate of evolution of the *BRh1 *opsin is consistent with our observation that this gene encodes the 437 nm visual pigment in *Lycaena rubidus*, which is a wavelength of peak absorbance that is more typical of the "blue-sensitive" visual pigments in butterflies, than the duplicate *BRh2 *gene in *L. rubidus *which encodes the unusually red-shifted 500 nm visual pigment [50]. Similarly, in the pierid *Pieris rapae*, the blue opsin copy (*B*), which encodes a 450 nm visual pigment [51] evolved slower than the violet copy (*V*), which encodes a more unusual 425 nm pigment, but this result was not significant either. Among the duplicated *LWRh *genes, significantly different rates of evolution were observed where *Bombyx mori LWRh2 *evolved slower than *B. mori LWRh1*, *Papilio LWRh2 *evolved slower than both *Papilio LWRh1 *and *Papilio LWRh3*, and *Apodemia mormo LWRh1 *evolved slower than *A. mormo LWRh2 *(Table 1). Here too, the slowest evolving *Papilio LWRh2 *encodes a pigment that with a λ_max _at 520 nm is functionally more similar to other butterfly long-wavelength sensitive visual pigments in its wavelength of peak absorbance than the pigment encoded by *Papilio LWRh3 *(λ_max _= 575 nm), similarly, the slower evolving *Apodemia LWRh1 *encodes a pigment that with a slightly blue-shifted λ_max _at 505 nm is much more typical of other butterfly pigments than its faster evolving *LWRh2 *copy that encodes a pigment with the highly atypical λ_max _at 600 nm [6,54]. Given that the relative rates tests seem able to classify the slowest and fastest evolving gene copies in a way that also roughly reflected their function, with the slowest evolving copies having spectral properties falling in a more narrow, similar and presumably ancestral range than the fastest evolving copies, we decided to divide our data for further analysis (see below) into alignments which included the slowest or fastest evolving copies.

**Table 1:** Tajima relative rates tests between duplicated copies of *BRh *and *LWRh *genes.

**Species**	*L. rubidus*	*L. heteronea*	*L. helloides*	*L. nivalis*	*P. icarus*	*A. glandon*	*S. behrii*	*P. rapae*
Gene copies (seq A/B)	*BRh1/2*	*BRh1/2*	*BRh1/2*	*BRh1/2*	*BRh1/2*	*BRh1/2*	*BRh1/2*	*BRhV/B*
Outgroup (seq C)	*A. mormo*	*A. mormo*	*A. mormo*	*A. mormo*	*A. mormo*	*A. mormo*	*A. mormo*	*P. xuthus*
N sites	1127	1126	994	1129	1115	1117	1139	1125
Unique differences seq A	101	98	80	90	112	113	97	125
Unique differences seq B	106	119	103	115	140	138	113	120
Unique differences seq C	122	125	109	123	110	112	135	115
χ^2 ^(1 d.f.)	0.12	2.03	2.89	3.05	3.11	2.49	1.22	0.10
*P*	0.728	0.154	0.089	0.081	0.078	0.115	0.270	0.750

**Species**	*B. mori*	*A. mormo*	*P. xuthus*	*P. xuthus*	*P. xuthus*	*P. glaucus*	*P. glaucus*	*P. glaucus*

Gene copies (seq A/B)	*LWRh1/2*	*LWRh1/2*	*LWRh1/2*	*LWRh1/3*	*LWRh2/3*	*LWRh1/2*	*LWRh1/3*	*LWRh2/3*
Outgroup (seq C)	*M. sexta*	*D. plexippus*	*P. rapae*	*P. rapae*	*P. rapae*	*P. rapae*	*P. rapae*	*P. rapae*
N sites	1128	1109	1137	1137	1137	1137	1137	1137
Unique differences seq A	107	97	145	114	97	156	117	90
Unique differences seq B	67	135	88	103	143	88	103	144
Unique differences seq C	114	123	116	141	105	104	145	106
χ^2 ^(1 d.f.)	9.20	6.22	13.94	0.56	8.82	18.95	0.89	12.46
*P*	**0.002**	**0.0126**	**0.0002**	0.455	**0.003**	**0.00001**	0.345	**0.0004**

*P*-values of gene pairs evolving at different rates are shown in bold.

For phylogenetic analysis and divergence time estimation, we also obtained *EF-1 α *and *COI *for individual taxa where such sequences were not already available in GenBank. A total of 66 new gene sequences, including 38 opsin genes, 15 *EF-1α *and 13 *COI *sequences, are reported in this study (See Additional File 2). Accession numbers for all new sequences and those downloaded from GenBank are shown in Additional File 1. The combined data set consisted of a total of 5523 bp, of which 1158, 1156, 1164, 1066 and 982 bp, belonged to the *UVRh*, *BRh, LWRh, EF-1α *and *COI *genes respectively.

### Maximum parsimony analysis recovers a single tree for butterflies

**Figure 1:**
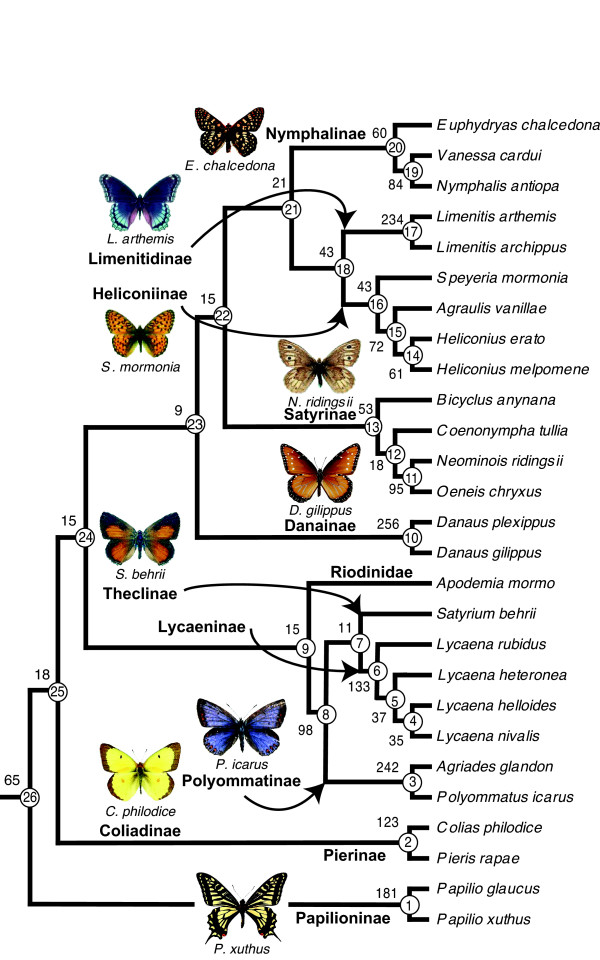
**Maximum parsimony tree from 5 genes and 2388 combined equally weighted parsimony-informative sites**. Numbers correspond to un-partitioned decay indexes (Bremer support values) for the data set containing the slower evolving gene copies of duplicated genes. Circled numbers label individual nodes. Representative images of most sampled subfamilies are shown. Wing sizes are not to scale.

Maximum parsimony (MP) analysis of all five genes using the slowest evolving opsin duplicates identified by the relative rates test resulted in a single tree (Figure 1, Additional File 1) with a topology congruent with that inferred in a previous study from molecular and morphological data [42]. Re-running this analysis using the faster evolving gene copies also revealed the same topology. As traditionally recognized, Papilionidae is placed as sister to (Pieridae + (Nymphalidae + (Lycaenidae + Riodinidae))). The relationships recovered within Nymphalidae also correspond to the current consensus, which groups Limenitidinae with Heliconiinae and Nymphalinae as sister to the previous two, Satyrinae as sister to (Nymphalinae + (Limenitidinae + Heliconiinae)) and Danainae as the basal subfamily, sister to (Satyrinae + (Nymphalinae + (Limenitidinae + Heliconiinae))). Within Lycaenidae, the Theclinae clade, represented by the hairstreak *Satyrium behrii*, groups together with the Lycaeninae, and these two clades are sister to the Polyommatinae.

Dissecting the contribution of different genes to this topological hypothesis through partitioned analysis reveals that the *LWRh *gene provides the strongest support to the topology, followed by the *BRh*, *UVRh*, *EF-1α *and *COI *genes (Table 2). This holds true for both combined analyses which included slower and faster gene copies of duplicate genes. Using the slower evolving copies we can see that both *LWRh *and *BRh *support all nodes, as shown by positive partitioned Bremer support values, whereas the information provided by *UVRh*, *EF-1α *and *COI *conflicts with one or more nodes. The UV opsin data strongly renders the grouping of both (Lycaenidae + Riodinidae), and (Nymphalidae + (Lycaenidae + Riodinidae)) spurious, and also does not support the monophyly of Satyrinae. *EF-1α *does not recover the (Lycaeninae + Theclinae) clade, but it does not take many steps to retrieve it (partitioned Bremer support -2, Table 2). *COI *conflicts with 10 out of the 26 total nodes, resulting in many negative support values (Table 2). This is not surprising since it has long been recognized that its fast rate of evolution renders *COI *of limited use when reconstructing phylogenetic history at levels deeper than species [33,55].

**Table 2:** Partitioned Bremer support values for nodes shown in Figure 1.

**Node**	**Node name**	***UV***	***B***	***LW***	***EF-1α ***	***COI***	**Total**
1	*Papilio*	69	39/43	49/72	12	12	181/208
2	*Colias + Pieris*	45/26	36/26	19/37	13/7	8	123/104
3	*Agriades + Polyommatus*	47	60/74	63/58	57	15	242/251
4	*L. helloides + L. nivalis*	13	11/9	7	0	4	35/33
5	*(L. helloides + L. nivalis) L. heteronea*	14	4/6	11	7/6	1/2	37/39
6	*((L. helloides + L. nivalis) L. heteronea) L. rubidus*	32	43/31	55/49	12/13	-9/-10	133/115
7	Lycaeninae + *Satyrium*	6	3/11	15/20	-2/-1	-11/-12	11/24
8	(Lycaeninae + *Satyrium)(Agriades + Polyommatus)*	17	35/40	37/30	3	6	98/96
9	Lycaenidae + *Apodemia*	-20	14/7	20/22	7	-6	15/10
10	*Danaus*	60/67	63/75	83/62	36/28	14/24	256
11	*Neominois + Oeneis*	24/23	0	36/34	27/28.5	8/6.5	95/92
12	*(N. ridingsii + O. chryxus) C. tullia*	2	0	12/6	6	-2	18/12
13	*((N. ridingsii + O. chryxus) C. tullia) Bicyclus*	22	12/10	8/10	4	7	53
14	*Heliconius*	22	19	13	12	-5	61
15	*Heliconius *+*Agraulis*	28	16	21	3	4	72
16	*(Heliconius *+ *Agraulis) Speyeria*	12	10	12/13	10	-1	43/44
17	*Limenitis*	48	67/68	46/47	44	29	234/236
18	*Limenitis + *node 16	9	15/17	10/7	6	3	43/42
19	*Vanessa + Nymphalis*	20/16	23/27	23/27	14/17	4/-1	84/86
20	*(Vanessa + Nymphalis) Euphydryas*	9.5	23	13/14	6.5	8	60/61
21	Node 18 + Node 20	12	1/4	7/1	2	-1	21/18
22	Node 21 + Node 13	-4	8/11	8/9	4	-1	15/19
23	Node 22 + Node 10	2/-4	2/3	4/5	0/4	1/-1	9/7
24	Node 23 + Node 9	-20/-4	14/3	20/5	7/4	-6/-1	15/7
25	Node 24 + Node 2	4	0/-2	8/5	0	6	18/13
26	Node 25 + Node 1	18/13	14/9	35/9	0/6	-2/2	65/39
	Total	491.5/479.5	532/540	635/594	290.5/289	88/95.5	2037/1998
	Percentage	24	26/27	31/29.7	14/14.5	4.3/4.8	100

Values represent support as calculated using data sets containing slower/faster evolving gene copies. Only one number is displayed when both analyses rendered the same value.

The Bremer support values rendered by the analysis of the combined data set using faster evolving gene copies are qualitatively and quantitatively similar to the values of the slower evolving copies in most cases (Table 2). A few exceptions occur at basal nodes in the tree, where using the faster evolving copy of duplicated genes eliminates the support of the *UVRh *and *COI *genes for Danainae as the most basal of the Nymphalidae (node 23, Figure 1 and Table 2), the support of the *BRh *gene for Pieridae as sister group to (Nymphalidae + (Lycaenidae + Riodinidae))(node 25), the support of *COI *for the node consisting of *Vanessa *+ *Nymphalis *(node 19), and adds the positive support of *COI *to Papilionidae as sister to (Pieridae + (Nymphalidae + (Lycaenidae + Riodinidae))) (node 26). However, these qualitative differences are not quantitatively important since the individual Bremer support values for these gene partitions approach zero in both slow and fast gene copy analyses of these nodes.

One possible reason for the poor performance of the *UVRh *gene in our data set is that there may be duplicate copies, especially at deeper nodes in the phylogeny, which we have not yet identified in these species that are missing from our analysis. When searching for opsins by screening cDNAs as we have done in the current study, it is difficult to know when to stop because there is far less functional data on the UV class of photoreceptor than either the blue or the long wavelength. This apparently messy result is interesting because it suggests we should continue to look harder. By contrast, for the *BRh *and *LWRh *genes, we think we have identified all but one of the duplicates in the species represented and so this may have contributed to their striking performance (but see below).

### Maximum likelihood and Bayesian phylogenetic analyses concur

**Figure 2:**
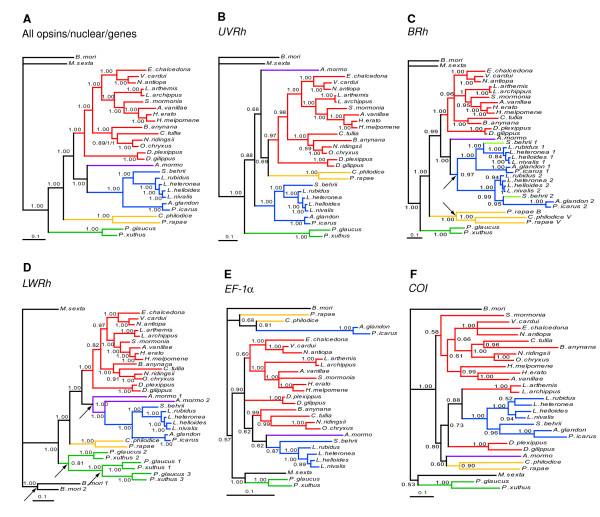
**Topologies obtained from Bayesian analyses of combined and individual gene data**. Numbers above and below branches represent clade support as posterior probabilities. Nodes with posterior probabilities below 0.5 are showed as polytomies. Branch lengths are shown as average substitutions per site. (A) An identical topology was obtained using the slower evolving copies of duplicated genes with numbers corresponding to posterior probabilities obtained from the all opsins/all nuclear/all genes datasets respectively. Only one support value is shown for clades in which all three data sets result in the same value. Traditional butterfly families are represented by the following colors: Papilionidae (green), Pieridae (yellow), Nymphalidae (red), Riodinidae (purple) and Lycaenidae (blue). (B-F) Bayesian topologies obtained for individual genes: (B) *UVRh*, (C) *BRh*, (D) *LWRh*, (E) *EF-1α*, (F) *COI*. *Satyrium behrii *is marked light green in the *BRh *gene tree to show how the two gene copies group with different lycaenid subfamilies. The six duplication events that generated the duplicated genes included in this study are indicated by black arrows.

To ensure that the good performance of the *BRh *and *LWRh *opsins and the poorer performance of the *UVRh *opsin was not an artifact of the tree reconstruction method we also performed maximum likelihood (ML) and Bayesian analysis of the data. Maximum likelihood and Bayesian analysis of the *LWRh *data set as well as the all opsins, all nuclear, all genes data sets using slower and faster evolving opsin copies resulted in identical, strongly supported topologies (Figure 2A and 2D, Additional Files 3 and 4) congruent with that obtained in our combined MP analyses (Figure 1), and with the inferred phylogenetic hypothesis of Wahlberg et al. (2005). The *BRh *gene rendered a nearly identical topology, with the only exception being an unresolved node joining the nymphalid subfamilies Satyrinae, Nymphalinae and Limenitidinae + Heliconiinae (Figure 2C, Additional File 3). In contrast, the *UVRh*, *EF-1α *and *COI *genes rendered non-traditional groupings with various degrees of bootstrap support (Figure 2B, 2E, 2F, Additional File 3).

**Figure 3:**
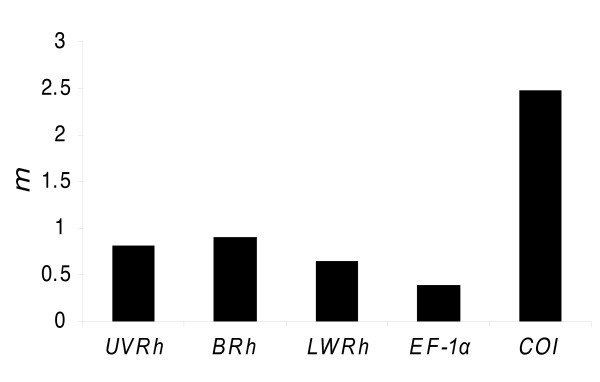
**Bayesian estimates of rate multiplier parameter (*m*) by gene partition using the slow gene data set**.

We also calculated using MrBayes a rate multiplier parameter (*m*) that reports relative substitution rate differences between partitions [4,56], which can vary widely between genes. As shown in Figure 3, rates of change for opsin genes are intermediate between fast evolving *COI *and comparatively slow evolving *EF-1α*, which can account for the strong phylogenetic signal contained by the *LWRh *and *BRh *genes at the hierarchical levels under examination in this study. On the other hand, the relatively lower performance of the *UVRh *is somewhat puzzling because its *m *falls in between that of the two other opsins.

Interestingly, of all 6 gene duplications included in our data sets (independent *BRh *duplications in Lycaenidae and Pieridae, two *LWRh *duplications in *Papilio *plus one *LWRh *duplication in *Bombyx mori *and one in *Apodemia mormo*), only one results in different groupings depending on which copy is utilized. Including *BRh1 *in the analysis resulted in the grouping of *Satyrium behrii *with the Lycaeninae under both maximum likelihood (93% bootstrap support, Additional File 3) and Bayesian analysis (Figure 2C), whereas using *BRh2 *creates either a polytomy of the three lycaenid subfamilies when employing ML (Additional File 3), or the grouping of Theclinae with Polyommatinae under a Bayesian framework (Figure 2C).

Wahlberg and Wheat [4] included a heat shock protein gene *HSP70 *in their initial study, and the resulting highly supported polyphyly of major lineages was blamed on the paralogy of heat shock protein genes. We note that it is highly likely that we would have had similar difficulties (i.e. either polyphyly or inappropriately clustered taxa) with a less complete sampling of paralogous genes in the current study. Such effects have been extensively documented elsewhere [3,57,58].

Regardless of the phylogenetic reconstruction method used, we found overwhelming support of a single phylogenetic hypothesis. Both the *LWRh *and *BRh *genes, as well as combined analyses of all opsin genes, all nuclear genes and all 5 genes rendered essentially the same tree topology, which also mirrors the current phylogenetic hypothesis for butterflies proposed by Wahlberg et al. [42] based on three genes (*COI*, *EF-1α *and *wingless*) and 99 morphological characters. Of the genes used in the above-mentioned study, *EF-1α *provided the strongest support to the topology, while *COI *the weakest (Table 2[42]). In their case, the addition of morphological data was crucial to elucidate many higher-level nodes, whereas in our study *LWRh *and *BRh *individually and in combination with other genes was sufficient to achieve this goal.

### Divergence time estimates using duplicate gene copies are similar at shallow nodes

To estimate divergence times we used two different relaxed clock methods and the same calibration points on the phylogenetic hypothesis shown in Figure 1. First we employed a semi-parametric rate smoothing penalized likelihood method [27], and second, we used a Bayesian approach [28]. We note that this is the first study of butterfly divergence times employing both methods. For calibration we used two fossils as well as a biogeographic event to constrain three nodes. The most recent common ancestors of *Vanessa cardui *and *Nymphalis antiopa *(node 19, Figure 1), and of *Pieris rapae *and *Colias philodice *(node 2) were constrained to a minimum age of 34 mya based on the Florissant fossils *Vanessa amerindica *[34,59] and *Stolopsyche libytheoides *[33,60], respectively, whereas the split between the Neartic *Papilio glaucus *and the Paleartic *P. xuthus *(node 1) was constrained between 35 and 65 mya, based on the breakage of the super continent Laurasia [61-63].

Under the Bayesian approach, three priors also need to be established, namely, the age of the root (node 26, Figure 1), the rate of evolution in substitutions per site per million years, and the variation of the rate of evolution over time, also called brownmean [28]. We selected 70 and 100 Ma as priors for the age of the ingroup node to reflect recent competing hypotheses for the origin of butterflies. Our 70 Ma prior follows the hypothesis of early butterfly diversification near the Cretaceous/Tertiary boundary (K/T, 65 Mya, [29]), and the 100 Ma prior reflects a conservative approach to a second hypothesis locating this event sometime in the Cretaceous, roughly following the early diversification of angiosperms 140 Mya [64]. Both the mass extinction of terrestrial vertebrates following the impact of a large meteor off the Yucatan Peninsula in Mexico defining the K/T boundary, and the rise of the angiosperms as dominant terrestrial plants had enormous evolutionary consequences, providing likely scenarios for the origin and diversification of insect taxa.

In general penalized likelihood (Additional File 5) reported older estimates than Bayesian analyses (Table 3, Additional Files 6 and 7), and the older the node the larger the difference between estimates obtained through the two methods and between estimates calculated using the slower and faster copies of duplicated genes. This is especially dramatic when we compare the estimates for when the oldest butterfly family, Papilionidae, is inferred to have first appeared (node 26). The penalized likelihood estimate using the slower evolving gene duplicates is much older (240.4 Mya) than the penalized likelihood estimate using the fast evolving gene copy (210.9 Mya) (Additional File 5), and both estimates are together older than those obtained using the Bayesian method, 113.1–197.4 Mya (95% confidence interval) for the slower evolving copies vs. 132.5–216.2 Mya for the faster evolving copies. It is important to note that the use of the three shallow level calibration points available under the restrictions of our data set constitutes a likely source of variation in our divergence time estimates, especially at deep nodes very distant in time from the calibrated nodes [65], so all of these estimates should be viewed with caution (See [18]).

**Table 3:** Bayesian 95% confidence intervals in millions of years for nodes shown in Figure 1.

**Node**	**Node name**	Slow (70 Ma)	Fast (70 Ma)	Slow (100 Ma)	Fast (100 Ma)
1	*Papilio*	47.2–64.8	45.4–64.8	47.8–64.8	45.8–64.7
2	*Colias *+ *Pieris*	56.4–94.1	70.6–115.0	56.6–94.7	70.7–115.3
3	*Agriades *+ *Polyommatus*	7.6–20.5	7.7–17.4	7.6–21.8	7.8–17.3
4	*L. helloides + L. nivalis*	3.8–10.0	5.4–12.8	3.8–10.3	5.5–12.7
5	(*L. helloides *+ *L. nivalis*) *L. heteronea*	7.5–18.4	9.5–21.3	7.6–18.8	9.6–21.0
6	*((L. helloides *+ *L. nivalis) L. heteronea) L. rubidus*	13.5–31.2	16.9–35.3	13.6–31.8	17.0–35.3
7	Lycaeninae + *Satyrium*	45.0–77.4	46.8–80.2	45.3–79.0	47.3–80.9
8	(Lycaeninae + *Satyrium*) (*Agriades *+ *Polyommatus*)	56.3–93.0	61.0–100.7	56.4–94.2	61.7–101.6
9	Lycaenidae + *Apodemia*	83.6–135.1	93.6–148.1	83.4–136.2	94.6–149.6
10	*Danaus*	15.1–34.7	17.6–35.8	15.3–35.6	17.9–35.6
11	*Neomimois *+ *Oeneis*	17.9–38.0	19.7–38.6	17.7–38.2	19.8–39.5
12	*(N. ridingsii *+ *O. chryxus*) *C. tullia*	49.7–81.7	54.1–88.2	49.7–82.0	54.7–89.5
13	*((N. ridingsii *+ *O. chryxus) C. tullia) Bicyclus*	58.6–93.3	64.4–101.6	58.4–93.6	64.8–102.9
14	*Heliconius*	12.3–25.3	13.6–25.9	12.4–25.6	13.5–26.1
15	*Heliconius *+ *Agraulis*	24.0–43.4	26.2–45.8	24.1–43.6	26.3–45.8
16	*(Heliconius *+*Agraulis) Speyeria*	41.2–66.8	44.8–72.1	41.4–67.1	45.3–72.6
17	*Limenitis*	4.7–13.6	5.2–12.1	4.7–13.5	5.3–12.1
18	*Limenitis *+ node 16	55.0–84.8	60.1–93.1	54.8–85.0	60.7–94.0
19	*Vanessa *+ *Nymphalis*	34.1–45.1	34.1–45.5	34.1–45.5	34.1–45.9
20	*(Vanessa *+ *Nymphalis) Euphydryas*	52.2–75.3	55.7–80.6	52.0–75.4	55.9–81.6
21	Node 18 + Node 20	70.0–107.3	78.3–119.1	69.8–108.1	78.9–120.3
22	Node 21 + Node 13	78.8–123.8	89.6–138.1	78.5–124.8	90.4–139.5
23	Node 22 + Node 10	86.1–138.2	100.1–156.7	85.6–138.5	101.3–158.3
24	Node 23 + Node 9	93.3–152.7	109.1–173.2	92.9–153.6	110.3–175.2
25	Node 24 + Node 2	101.1–170.0	118.5–190.0	100.6–170.1	119.7–192.0
26	Node 25 + Node 1	113.1–197.4	132.5–216.2	112.8–197.9	134.0–218.9

Estimates were calculated using combined data sets including slow and fast evolving opsin copies. These results were obtained using a prior distribution for the age of the ingroup node (root age) of either 70 (± 70) or 100 (± 100) Ma, a rate of evolution of 0.002 substitutions per site per million years and brownmean value of 0.02.

**Figure 4:**
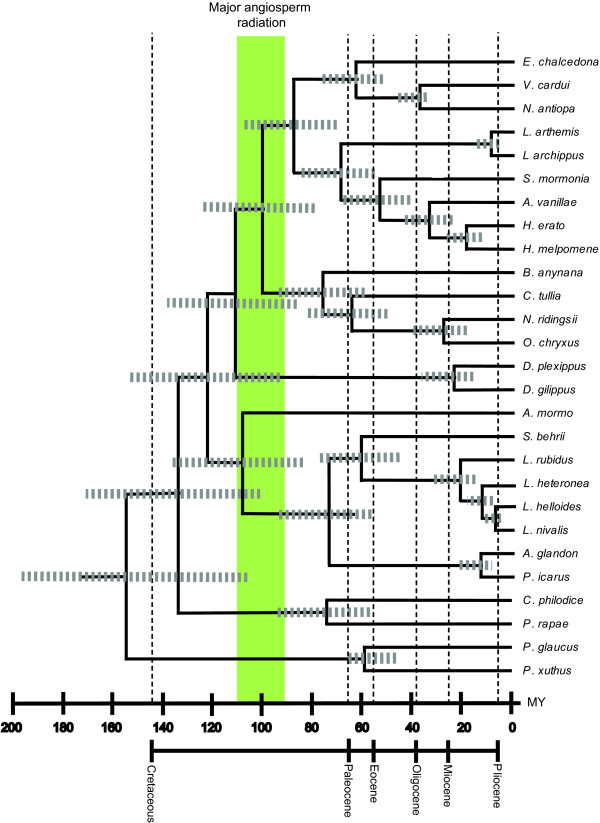
**Divergence time estimates using the Bayesian method on the slow copy combined data set**. Estimations were performed using the combined five gene data set using priors of age of ingroup node = 70 Ma, rtrate = 0.002 substitutions per site per million years and brownmean = 0.02. For each estimate 95% confidence intervals are shown in grey. Green bar indicates the major period during which flowering plants diversified, 90–115 Mya [72]. The lower bounds for the 95% confidence intervals for the lineages leading to all butterfly families falls within or after this period.

The Bayesian analyses performed on combined data sets containing slower evolving copies of duplicated genes rendered younger estimates, and therefore more conservative estimates than the analyses of combined data sets including faster evolving gene copies or the analyses using penalized likelihood (Figure 4, Additional File 8). Since the effect of the priors employed in the analyses is small, and the choice of a root age of 70 and 100 Ma did not seem to have much of an impact on divergence dates (Table 3) the rest of this discussion will be based on the divergence times estimated using the slower evolving copies, our preferred rate and brownmean priors (0.002, 0.02, respectively) and our most conservative root age prior of 70 Ma (see methods section).

Our results indicate that the Papilionidae diverged from the ancestor of all the other butterfly families combined sometime between 113.1–197.4 Mya (95% confidence interval) using the slowest evolving copies of duplicated genes (node 26, Table 3). These results are compatible with other studies focused on divergences of younger lineages within the family. Zakharov and collaborators suggest for instance the ancestor of *Papilio *diverged from the Tribe Troidini up to 100 Mya [63] while Braby et al., (2005)[32] propose a minimum age of 90 Ma for the first diversification within Troidini. We also note that two other studies [35,63] using similar calibration constraints as our study, timed the major divergences within the papilionid genus *Papilio *as occurring around 57.9 (37.8–78.0) and 57.9 (43.0–72.8) Mya, respectively, which is comparable with our estimated divergence of the lineages leading to *P. glaucus *and *P. xuthus *between 47.2–64.8 Mya (node 1, Table 3).

We estimate the split of Pieridae, the next oldest butterfly lineage to have evolved, from the ancestor of (Nymphalidae + (Lycaenidae + Riodinidae)) to be situated between 101.1–170.0 Mya (node 25, Table 3), a result consistent with Wheat et al. [36] who estimated this split to have occurred between 79 and 166 Mya. Similarly, Braby et al., (2006) [33] estimated the subtribes Pierina and Aporiina to have diverged 58 Mya, and from this result extrapolated an average age for the crown group of the Pieridae of 95 Ma (99.9% confidence interval between 82 and 112 Mya). At the subfamily level, our results situate the split between Pierinae and Coliadinae (node 2, Table 3) in the Pieridae between 56.4–94.1 Mya, which is consistent with the results of Wheat et al., (2007) [36], who, using distance methods on the *EF-1α *and *COI *genes alone, estimate this divergence is between 62 and 96 Mya.

For the youngest butterfly families, the most recent common ancestor of Nymphalidae and (Lycaenidae + Riodinidae) (node 24, Table 3), is located by our analyses at a minimum age between 93.3–152.7 Ma, and the split between Lycaenidae and Riodinidae (node 9, Table 3) at 83.6–135.1 Mya. Estimates for the origins of subfamilies within the Lycaenidae (Theclinae, Lycaeninae and Polyommatinae) and Nymphalidae (Danainae, Limenitidinae, Nymphalinae, Heliconiinae, and Satyrinae) are shown in Table 3. Wahlberg (2006) [34] suggests that the nymphalid subfamily Nymphalinae was already present the Cretaceous/Tertiary boundary, 65 Mya. In our data set, the first split within Nymphalinae (node 20, Table 3) occurs between 52.2–75.3 Mya, which is concordant with Wahlberg's estimate. We note too that Peña and Wahlberg (2008) [37] estimated the split between Satyrinae and the closely related subfamily Calinaginae (missing from our data set), to have occurred 80.5 ± 4.9 Mya. Though not directly comparable, this result is compatible with our estimated age 78.8–123.8 Mya for the divergence of Satyrinae from its more distantly related subfamilies (Nymphalinae + (Limenitidinae + Heliconiinae))(node 22).

On the other hand, Monteiro and Pierce (2001), using a mitochondrial gene molecular clock estimated the origin of the satyrine genus *Bicyclus *between 15 and 20 Mya, and the split of the subtribe to which it belongs (Mycalesina: Satyrini) from the subtribe to which *Coenonympha *belongs (Coenonymphina: Satyrini) at roughly 35 Mya (extrapolated from Figure 1, [37]). This last estimate is much younger than our estimation for the split between *Bicyclus *and (*Coenonympha + *(*Neominois + Oeneis*)) which we find between 58.6–93.3 Mya (node 13, Table 3). This discrepancy may be partly due to the currently unresolved polytomies that plague the Satyrini tribe [66].

The only divergence time estimate existent for species in the widely studied nymphalid genus *Danaus*, to which the famed monarch butterfly belongs (*D. plexippus*), corresponds to the divergence between the sister species *D. plexippus *and *D. erippus*, which based on molecular clock estimates are suggested to have split about 2 Mya [67]. Our analyses situate the split between the less closely related *D. plexippus *and the queen butterfly *D. gilippus*, between 15–35 Mya (node 10). As expected based on its position in the butterfly phylogeny, the scientifically conspicuous Danainae originated prior to the Limenitidinae (Table 3), which contains the palatable species *Limenitis archippus*, co-mimic of both the monarch and queen butterflies. We estimated the divergence between the North American *L. archippus archippus *and *L. arthemis astyanax *(the red spotted purple, which is a palatable mimic of the papilionid *Battus philenor*)(node 17), to have occurred between 4.7–13.6 Mya. This result conflicts with a previous estimate of the colonization of the Nearctic by the genus *Limenitis*, situated at about 4 Mya by a molecular clock calculation but recognized by the author to be based on a crude divergence rate percentage [68], but is compatible with the postulated Laurasian origin of the genus *Limenitis *and the Bering land bridges that connected Asia with North America in the late Miocene (11.608 ± 0.005 to 5.332 ± 0.005 Mya) [69].

Another taxon famous for its astounding mimicry complexes is the nymphalid genus *Heliconius*, for which, surprisingly, only two estimates of divergence times exist in the literature; for intraspecific or more-closely related species in *Heliconius *than those included in our study. Based on arthropod mitochondrial gene evolution rates, Brower found the first split *within H. erato *and *H. melpomene *to have occurred between 1.5 and 2 Mya [70], whereas Kronforst (2008)[71], using the same method, dated the split between the closely related *Heliconius melpomene*/*cydno *and silvaniform clades, which excludes *H. erato*, at 2.5 Mya. Our study includes *H. melpomene *and *H. erato*, members of branches representative of the first divergence within the genus, and estimates their split between 12.3–25.3 Mya (node 14).

### Did butterflies diversify in the age of angiosperms?

Butterflies are phytophagous feeders as adults and larvae on the flowering plants or angiosperms. Using all fossil evidence, including leaves, flowers, wood and pollen, the major angiosperm radiation is estimated to have occurred 90–115 Mya (Figure 4 and Additional File 8) [72]. In light of this information, the molecular timescale estimates we have presented that seem most reasonable for the butterflies are the lower bounds of the 95% confidence intervals for the slow evolving data set (Table 3). Considering the lower bounds, our data suggest that diversification of the lineages leading to the five butterfly families was completed sometime after the angiosperm radiation, ~84 Mya.

## Conclusion

Our results suggest the use of *LWRh *and *BRh *opsins in resolving both lower and higher level phylogenetic relationships in butterflies and suggest they may help to resolve relationships and divergence times estimates between even larger and more complex groups such as the moths. One possible reason for the unusually good performance of *LWRh *and *BRh *opsins may be due to the fact that we sampled highly expressed transcripts, which may bias the sample towards copies that for structural reasons are evolving under independent trajectories. It is very likely that if we had targeted genomic DNA, then we would have been more likely to pick up the kinds of duplicate opsin genes that are to be avoided for phylogenetic purposes, e.g., tandemly repeated copies undergoing gene conversion.

Another possibility is in the way we have handled this data. We propose that phylogenomic studies might usefully include duplicate gene copies for phylogenetic analysis if relative rates tests are first used to identify slow and fast evolving copies. In our data set where the spectral properties of some of the visual pigments encoded by duplicate copies are known, this simple procedure brought together a collection of genes in the slow category whose ancestral function has been retained and whose patterns of molecular evolution may have been conducive to phylogenetic reconstruction and divergence times estimation. Even for genes where no functional information is known a similar procedure may help, though of course this suggestion would best be implemented after further validation using genes with a range of other functions besides the visual pigments.

As several previous studies on butterfly family and subfamily divergence times have suggested, so too do our results support an origin of butterflies older than the 70 Ma advocated by Vane-Wright [29] and others, regardless of the absence of butterfly fossils older than Eocene ages. The present study, using a set of genes that includes opsins, which have not been used previously in butterfly divergence time estimations, reaches the same conclusion: the origin of butterflies surpasses with ample margin the Cretaceous/Tertiary boundary by at least 20 Ma.

## Methods

### Tissue Collection

Most butterflies were included based on initial studies, which indicated the potential utility of the *LWRh *gene for reconstructing family and subfamily-level relationships [6], and the availability of fossil and/or biogeographical calibration points (See discussion). These butterfly taxa were resampled (this study) for their *UVRh, BRh, EF-1α *and *COI *sequences. Other species were included because they are currently being developed as model systems in butterfly ecology and evolutionary biology. All specimens were collected as adults in the field and immediately placed in RNALater (Applied BioSystems/Ambion, Austin, TX) or freshly frozen (*Euphydryas chalcedona*, *Speyeria mormonia, Satyrium behrii *and *Agriades glandon*, Mono County, CA;*Agraulis vanillae*, Huntington Beach, CA; *Coenonympha tullia *and *Oeneis chryxus*, Boulder, CO;*Lycaena heteronea, L. helloides *and *L. nivalis*, Gunnison County, CO). The remaining specimens were kindly provided as gifts (*Nymphalis antiopa*, Irvine, CA, Peter Bryant; *Limenitis arthemis astyanax*, Baltimore County, MD, Austin Platt; *L. archippus archippus*, Franklin County, MA, Fred Gagnon; *Danaus plexippus*, Bradford County, FL, Edith Smith;*D. gilippus*, Collier County, FL; *Heliconius erato *and *H. melpomene*, Costa Rica, Larry Gilbert; *Neominois ridingsii*, Montrose County, CO, Matthew Garhart; *Lycaena rubidus *and *Colias philodice*, Gunnison County, CO, Ward Watt and Carol Boggs; *Polyommatus icarus*, Germany, Almut Kelber and *Apodemia mormo*, Hemet, CA, John Emmel).

### PCR, Cloning, and Sequencing

For *E. chalcedona*, *N. antiopa, H. erato, A. vanillae, S. mormonia, C. tullia, N. ridingsii, L. heteronea, L. helloides, L. nivalis, A. mormo, L. arthemis astyanax*, *L. archippus archippus, D. gilippus, H. melpomene, O. chryxus, S. behrii, A. glandon *and *C. philodice*, total RNA was extracted from one head with Trizol (GibcoBRL), and cDNA synthesized using the Marathon cDNA Amplification Kit (BD Biosciences Clontech, Mountain View, CA). The cDNA was then utilized in 3' RACE (rapid amplification of cDNA ends) PCR (BD Adv 2 Polymerase Mix) with, in the case of opsin genes, the adaptor primer AP1 and an arthropod opsin-specific degenerate primer (5'-GAA CAR GCW AAR AAR ATG A -3'). PCR products were gel purified (Geneclean kit, QBioGene), incubated for 10 min at 72°C with 0.5 μl *Taq *DNA polymerase (Promega) to add A-overhangs, cloned into pGem T-easy vector systems (Promega, Madison WI) and sequenced (BigDye^® ^Terminator v3.1 Cycle Sequencing Kit, Applied Biosystems) at the University of California, Irvine DNA core sequencing facilities. Duplicate gene transcripts were obtained by performing multi-plex PCR on additional clones to identify templates that did not amplify with the opsins picked up in this initial procedure. To obtain complete *UVRh, BRh *and *LWRh *opsin sequences, gene specific reverse primers were designed from the fragments and used to amplify the 5' RACE products (Additional File 9).

From these cDNAs we also amplified fragments of the mitochondrial *cytochrome oxidase subunit I *(*COI*) gene from *H. erato *and *S. mormonia *and the nuclear *elongation factor 1 alpha *(*EF-1α*) gene from *H. erato*, *S. mormonia, O. chryxus, S. behrii, A. glandon *and *A. mormo *using the primers mRon (5'- GGR GCH CCH GAT ATA GCH TTY CC -3') and mHobbes (5'-AAA TGT TGD GGN AAA AAD GTT A-3') for *COI *modified from Monteiro and Pierce (2001), and EF44(f) (5'-GCY GAR CGY GAR CGT GGT ATY AC-3') and EFrcM4(r) (5'-ACA GCV ACK GTY TGY CTC ATR TC-3') for *EF-1α *[73].

Standard phenol:chlorophorm extraction of genomic DNA was performed on one individual adult per species from *L. arthemis astyanax, L. archippus archippus, D. plexippus, D. gilippus, H. melpomene, O. chryxus, L. rubidus, S. behrii, A. glandon, P. icarus*, and *C. philodice*. From these pools of genomic DNA we obtained the *COI *and *EF-1α *genes from *D. plexippus, D. gilippus, H. melpomene, L. rubidus*, and *C. philodice*; the *COI *gene from *O. chryxus, S. behrii, A. glandon *and *P. icarus*; and the *EF-1α *gene from *L. arthemis astyanax *and *L. archippus archippus *using the primers described above.

### Phylogenetic Analysis

For each gene we aligned our sequences and others obtained from GenBank (Additional File 1) using MEGA 3.1 [74] and by hand. Besides the 5 individual gene data sets, we constructed 3 concatenated data sets combining all 3 opsin genes (*UVRh + BRh + LWRh*), all 4 nuclear genes (*UVRh + BRh + LWRh + EF-1α*) and all 5 genes (*UVRh + BRh + LWRh + EF-1α + COI*), respectively. Because not all sampled taxa possess the same duplications, to prepare these concatenated data sets we first chose which copies of the duplicated genes to use in the alignments (Additional File 2). We chose by comparing the rate of evolution of different paralogous gene pairs using the relative rate tests as implemented in MEGA 3.1 and selecting for a first round of analyses the slowest evolving copy for inclusion (except in the case of the pierid blue opsin gene duplication, in which we chose for all analyses the V (violet) opsin gene, due to the absence of *C. philodice BRh *gene from our data set). The resulting nucleotide alignments were used to reconstruct phylogenetic relationships by maximum-parsimony (MP), maximum-likelihood (ML) and Bayesian methods. We then repeated all 3 concatenated analyses on a second set of alignments, this time including the faster evolving paralogous copies of duplicated genes. We rooted our trees with two moth species, the sphingid *Manduca sexta *and the bombycid silkworm *Bombyx mori*. See Additional File 1 for GenBank numbers of sequences included in slow vs. fast data sets.

The incongruence length difference (ILD) test [75] implemented as partition homogeneity test in PAUP 4.0 for assessing incongruence between character sets showed that the three opsin gene partitions are congruent regardless of whether slower (*P *= 0.057) or faster (*P=*0.191) evolving copies are used, but the addition of *EF-1α *and *EF-1α *+ *COI *to the opsin data results in incongruent data sets in which the different partitions are evolving non-homogeneously (*P *< 0.001 for both slower/faster data sets for both combinations). Since the utility of the ILD test has been challenged [76], however, we analyzed the data partitioned (by gene) and un-partitioned. Maximum parsimony analyses were run using heuristic searches, tree bisection-reconnection (TBR) branch swapping algorithm with gaps treated as missing data and all characters equally weighted in PAUP 4.0 [77]. Clade robustness was evaluated using decay indexes (Bremer support values) using PAUP 4.0 and TreeRot [78]. Partitioned Bremer support (PBS) values were also calculated to determine the relative contribution of the 5 gene partitions to the total Bremer support of the combined, un-partitioned analysis.

For all data sets, including individual and concatenated gene sequences, the optimal nucleotide substitution model chosen by Modeltest was the most complex available to date: GTR with proportion of invariant sites (I) and gamma-distributed rates (G). Maximum likelihood analyses were conducted in PHYML online web server [79,80] for all 8 data sets and the reliability of the trees obtained was tested by 500 bootstrap replicates. The optimal DNA substitution model for each data set was determined by nested likelihood ratio tests as implemented in Modeltest 3.7 [81]. The GTR + I + G (general time reversible plus proportion of invariant sites and gamma-distributed rates for sites) substitution model was selected in every case. Proportion of invariant sites and gamma shape parameters were estimated in PAUP 4.0.

As for the Bayesian phylogenetic reconstruction method, we chose the GTR + I + G model of nucleotide evolution for all 8 data sets, where the proportion of invariant sites and the shape of the gamma parameter were estimated for each gene or partition employed. Bayesian analyses were performed using MrBayes 3.1.2 [82] on all 8 unpartitioned data sets, and repeated with the all genes data set under 2 partitioning schemes: by gene (5 partitions) and by gene and codon position (5 genes × 3 positions = 15 partitions) under the same model selected for ML analyses (GTR+I+G). In a partitioned analysis the model parameters (in this case, I and G) are calculated separately for each partition. For each data set, slow and fast, 4 chains, 3 heated and 1 cold, were run simultaneously for 4 × 10^6 ^generations sampled every 100^th ^generation. The first 20000 trees were discarded as burn-in samples. The remaining trees were used to generate a majority rule consensus tree, in which the percentage of samples recovering a particular clade represents its support measured as posterior probabilities. Resulting tree files were inspected in Tree View [83]

### Divergence Time Estimation

We performed two analyses on the combined data set of 5 genes, first using the slower evolving copies of duplicated genes, as determined by relative rate tests, and second using the faster evolving copies. Initial results for the semi-parametric penalized likelihood method were obtained with the default settings of r8s using cross-validation to find the smoothing parameter resulting in the lowest cross validation scores. We found this smoothing parameter to be 3.2 for both slow and fast data sets. Using this value and branch lengths estimated by maximum likelihood in PHYML we repeated the analysis and estimated the divergence ages presented in Additional File 5.

For the Bayesian analysis, the rate of evolution prior was selected based on the root to tip median branch length of our slow and fast trees, a proxy advocated by Thorne and Kishino (2002) [28] and explained in detail by Wiegmann et al., (2003)[84]. These branch lengths were calculated using the GTR + I + G ML model of evolution in PAUP 4.0. For both slow and fast data sets the median branch length, divided by the prior of root age resulted in a value between 0.003 and 0.005 depending on the root age prior; therefore for our preferred estimates we utilized a prior of 0.002 ± 0.002 (standard deviation). The prior for the variation in the rate of evolution over time (brownmean) was set to 0.02 ± 0.02 based on the empirical suggestion of [28] and [84], which states that a preferred brownmean multiplied by the root age prior should result in a number between 1 and 2. Large standard deviations were chosen for all priors to increase the flexibility of our analyses considering the lack of detailed information about the actual divergence times and rates of evolution of butterflies.

Under the Bayesian framework, and for both slower and faster data sets we performed analyses using all 18 permutations of prior values, which included a prior distribution for age of the ingroup node of either 70 (± 70) or 100 (± 100) Mya, a prior for the rate of molecular evolution of either 0.02 ± 0.02, 0.002 ± 0.002 or 0.0002 ± 0.0002, and a prior for the variation of the rate of evolution over time (brownmean) of either 0.02 ± 0.02, 0.002 ± 0.002 or 0.0002 ± 0.0002. Parameters were sampled after a burn-in period of 100,000 cycles, for an additional 1,000,000 cycles sampled every 100. Divergence time estimates were calculated from these 10,000 samples.

## Abbreviations

Ma: million years; Mya: million years ago; *UVRh*: ultraviolet-sensitive rhodopsin; *BRh*: blue-sensitive rhodopsin; *LWRh*: long wavelength-sensitive rhodopsin; *EF-1α*: elongation factor 1-alpha; *COI*: cytochrome oxidase I.

## Authors' contributions

NP carried out molecular genetic studies, phylogenetic analysis, divergence time estimation and drafted the manuscript. MSM carried out molecular genetic studies, sequence alignment, phylogenetic analysis, primer design and coordination of the project. EY and SL carried out molecular genetic studies. ADB conceived of the study, participated in its design and coordination, analyzed data, and revised the manuscript. All authors have read and approved the manuscript.

## Supplementary Material

Additional file 1 **Taxa and genes used in this study**. GenBank accession numbers for genes newly sequenced and included in study. Click here for file

Additional file 2 **Sequence alignments used in this study**. Amino acid alignments of UVRh, BRh, LWRh, EF-1α and COI. Click here for file

Additional file 3 **Trees from combined and individual analyses of slow-evolving-copy genes**. The maximum likelihood topologies presented were recovered using the same alignment used to generate Figure 1 (maximum parsimony) and Figure 2 (Bayesian). Click here for file

Additional file 4 **Trees from combined and individual analyses of fast-evolving-copy genes**. The maximum parsimony, maximum likelihood and Bayesian analyses of fast-evolving-copy genes recovered identical topologies. Click here for file

Additional file 5 **Penalized likelihood age estimates**. The data provided represent estimates calculated from alignments including either slow- or fast-evolving copies of duplicate genes. Click here for file

Additional file 6 **Bayesian age estimates**. The data provided represent estimates obtained using the slow-evolving-copy gene data set and several combinations of prior values. Click here for file

Additional file 7 **Bayesian age estimates**. The data in the table represent estimates obtained using the fast-evolving-copy gene data set and several combinations of prior values. Click here for file

Additional file 8 **Divergence time estimates**. The data in the figure show the impact of incorporating fast-evolving genes on Bayesian divergence time estimates using priors of age of ingroup node = 70 Ma, rtrate = 0.002 substitutions per site per million years and brownmean = 0.02. Click here for file

Additional file 9 **Primers used in the study**. The sequences represent primers used in 5'RACE of opsin genes. Click here for file
