# Supplementary material for: Impact of duplicate gene copies on phylogenetic analysis and divergence time estimates in butterflies
Source: BMC Evol Biol. 2009 May 13;9:99. doi: 10.1186/1471-2148-9-99 (PMC2689175; doi:10.1186/1471-2148-9-99)
Supplement: Additional file 1 — Taxa and genes used in this study. GenBank accession numbers for genes newly sequenced and included in study. [file 1471-2148-9-99-S1.doc]

**Additional File 1.** Taxa and genes used in this study. In bold are sequences added by this study. Slower and faster evolving gene copies used in combined analyses are marked by * and ^&^ respectively. Parentheses indicate duplicate gene names.

| **Family** | **Subfamily** | **Species** | ***UVRh*** | ***BRh*** | ***LWRh*** | ***EF1α*** | ***COI*** | ***Locality/collector*** |
| --- | --- | --- | --- | --- | --- | --- | --- | --- |
| Nymphalidae | Nymphalinae | *Vanessa cardui* | AF414074 | AY613987 | AF385333 | AY248807 | AY248782 |  |
|  | Nymphalinae | *Euphydryas chalcedona* | **EU449014** | **EU358776** | DQ924373 | AY788744 | AF187752 | California: Mono Co. |
|  | Nymphalinae | *Nymphalis antiopa* | **AY918892** | AY918893 | AY740907 | AY218266 | AY218246 | California: Irvine, Peter Bryant |
|  | Limenitinae | *Limenitis arthemis asyanax* | **AY918901** | AY918902 | AY918903 | **DQ157895** | DQ205131 | Maryland: Baltimore Co. Austin Platt |
|  | Limenitinae | *Limenitis archippus archippus* | **EU449016** | **EU358777** |  | [**DQ208217**](http://www.ncbi.nlm.nih.gov/entrez/viewer.fcgi?db=nuccore&val=77999803) | DQ205114 | Massachussets: Franklin Co. Fred Gagnon |
|  | Danainae | *Danaus plexippus* | AY605546 | AY605544 | AY605545 | **DQ157894** | **EU330440** | Florida: Bradford Co., Edith Smith |
|  | Danainae | *Danaus gilippus* | **EU449017** | **EU358779** | **EU352197** | **EU326286** | **EU330435** | Florida: Collier Co. Gulf Coast Butterflies |
|  | Heliconiinae | *Heliconius erato* | AY918904 | AY918906 | AY918907 | **DQ157892** | **EU330441** | Costa Rica Larry Gilbert |
|  | Heliconiinae | *Heliconius melpomene* | **AY918896** | AY918897 | **EU480690** | DQ448447 | **EU449025** | Costa Rica Larry Gilbert |
|  | Heliconiinae | *Agraulis vanillae* | **EU449018** | **EU358780** | DQ924367 | DQ922873 | DQ922841 | California: Huntington Beach. |
|  | Heliconiinae | *Speyeria mormonia* | **EU449019** | **EU358781** | DQ924366 | **EU326287** | **EU330436** | California: Mono Co. |
|  | Satyrinae | *Coenonympha tullia* | **EU449020** | **EU358782** | DQ924374 | AF173399 | AF170860 | Colorado: Boulder. |
|  | Satyrinae | *Bicyclus anynana* | AF484248 | [AY918894](http://www.ncbi.nlm.nih.gov/entrez/viewer.fcgi?db=nuccore&id=62860639) | AY918895 | AY218258 | AY218238 | Antonia Monteiro |
|  | Satyrinae | *Neominois ridingsii* | **EU449021** | N/A | DQ924377 | DQ339026 | DQ338870 | Colorado: Montrose Co. Matthew Garhart |
|  | Satyrinae | *Oeneis chryxus* | **EU449022** | N/A | DQ924378 | **EU326283** | **EU330437** | Colorado: Boulder. |
| Lycaenidae | Lycaeninae | *Lycaena rubidus* | AY587904 | AY587902(1)*  AY587903(2)^&^ | AY587901 | **DQ157891** | **EU330442** | Colorado: Gunnison Co. Carol Boggs |
|  | Lycaeninae | *Lycaena helloides* | **DQ517940** | **DQ517943(1)***  **DQ517946(2)**^&^ | **DQ517949** | AY954622 | AY954562 | Colorado: Gunnison Co. |
|  | Lycaeninae | *Lycaena heteronea* | **DQ517941** | **DQ517944(1)***  **DQ517947(2)**^&^ | **DQ517950** | **EU326289** | **EU330432** | Colorado: Gunnison Co. |
|  | Lycaeninae | *Lycaena nivalis* | **DQ517942** | **DQ517945(1)***  **DQ517948(2)**^&^ | **DQ517951** | **EU326288** | **EU330433** | Colorado: Gunnison Co. |
|  | Theclinae | *Satyrium behrii* | **EU449023** | DQ402498(1)*  DQ402499(2)^&^ | **EU352198** | **EU326284** | **EU330438** | California: Mono Co. |
|  | Polyommatinae | *Agriades glandon* | **EU449024** | DQ402502(1)*  DQ402503(2)^&^ | **EU352199** | **EU326285** | **EU330439** | California: Mono Co. |
|  | Polyommatinae | *Polyommatus icarus* | EU088115 | DQ402500(1)*  DQ402501(2)^&^ | EU088114 | AY496846 | **EU330434** | Germany: Almut Kelber |
| Riodinidae | Riodininae | *Apodemia mormo* | **AY587905** | AY587906 | AY587907(1)*  AY587908(2)^&^ | **EU520324** | AF170863 | California: Hemet John Emmel |
| Pieridae | Coliadinae | *Colias philodice* | **AY918898** | AY918899 (V) | **AY918900** | **DQ157890** | **EU330443** | Colorado: Gunnison Co. Ward Watt & Carol Boggs |
|  | Pierinae | *Pieris rapae* | AB208673 | AB208675 (B)  AB208674 (V) | AB177984 | AY870550 | [AY954581](http://www.ncbi.nlm.nih.gov/entrez/viewer.fcgi?db=nuccore&id=63030194) |  |
| Papilionidae | Papilioninae | *Papilio glaucus* | AF077191 | AF077192 | [AF077189](http://www.ncbi.nlm.nih.gov/entrez/viewer.fcgi?db=nuccore&id=4959066)(1)^&^  AF077190(2)*  AF098283(3) | EU136675 | AF044013 |  |
|  |  | *Papilio xuthus* | [AB028218](http://www.ncbi.nlm.nih.gov/entrez/viewer.fcgi?db=nucleotide&val=7415633) | AB028217 | AB007423(1)^&^  AB007424(2)*  AB007425(3) | AF044838 | AF043999 |  |
| Sphingidae |  | *Manduca sexta* | L78081 | AD001674 | L78080 | AF234571 | U09843 |  |
| Bombycidae |  | *Bombyx mori* | AADK01002778 | AADK01014448 | AADK01025811  AADK01025594 | D13338 | AF149768 |  |
