# Supplementary material for: Impact of duplicate gene copies on phylogenetic analysis and divergence time estimates in butterflies
Source: BMC Evol Biol. 2009 May 13;9:99. doi: 10.1186/1471-2148-9-99 (PMC2689175; doi:10.1186/1471-2148-9-99)
Supplement: Additional file 2 — Sequence alignments used in this study. Amino acid alignments of UVRh, BRh, LWRh, EF-1α and COI. [file 1471-2148-9-99-S2.doc]

**Additional File 2a.** Alignment of 29 lepidopteran UVRh complete coding sequences.

*V. cardui* MIP-VLNMDNKTENYNIYGAYFAPLSSSDGIKMLVDGLEGEDLAAVPEHWFSYAAPPASAHTALALLYCFFTAAALIGNG 80

*E. chalcedona* ...-S.T.E....D................V.............SI......................S........... 80

*N. antiopa* ...-............S.............V.............VI........................L......... 80

*L. arthemis* ...-..K...E...N.V.............V....E..NS..M.VM....L..T.......................... 80

*L. archippus* ...-..K...E...N.V.............V....E..NS..M.VM....L..T.......................... 80

*D. plexippus* ...-TIT...D.D.I.V...........E.T...M...T...........HT.PS......................... 80

*D. gilippus* ...-TIT...E.D.F.V.............M...M...T...........HT.T..............T........... 80

*H. erato* -------.E.E.Q...V...F.........V.......DSV...VM....L..TS.............S........... 80

*H. melpomene* -------.E.E.Q...V.............V.......DSA...V.....L..P.......................... 80

*A. vanillae* .M.NT.K.E.D.Q...V.............V.......DS....VM....L..T..............S........... 80

*S. mormonia* ...-.MK.E.D.D..SA.............V.......DSD...VM....L..T......................M... 80

*C. tullia* .L.-TAT...D.HDF.T..........-G.M.............M......T.E.......................... 80

*B. anynana* .M.-T.....D.Q.F.T..........V..S....E.....A...I.D..M...S......................... 80

*N. ridingsii* ...-TI.V..D.Q.F.T.............TA..................M............................. 80

*O. chryxus* ...-T.....D.H.F.T.............T...................M............................. 80

*L. rubidus* ...-S.S..T.N.S.HL............EPE......T....E......L..T..............I........... 80

*L. helloides* ...-S.V..I.N.S.HL.............SE..G...T....E......L..P..............I........... 80

*L. heteronea* ...-S.V....N.S.HL.............SE..G...T....E......L..P..............I.L......... 80

*L. nivalis* ...-S.V....N.S.HL.............SE..G...T....E......L..P..............I........... 80

*S. behrii* .V.-S.D..SHN.T.HL.............SE..G...T...........L..P..............M.L......... 80

*A. glandon* ...-PMD..-.N.T.HL............AVV..GE..T.....Q.....L..P.......M......T........... 80

*P. icarus* ...-PMD..-.N.T.HL............AME..GE..T.....Q.....L..P..............T........... 80

*A. mormo* ...-S.S.E.S..S.SM..........-.ETE..G...T...........L..H..............S...V...V... 80

*C. philodice* ...-TQD...S..Y.............-G.ER..GE..T.....MM.....K.PE.........C............... 80

*P. rapae* ...-TQD...S..Y.............-G.E...GE..T....EMM.....K.PE.........C............... 80

*P. glaucus* ..A-PAA...H...NYN.......Y..DEPVE..GA..T.A....I....LA.P........M...V.V........... 80

*P. xuthus* ...-AAV...H...NYN.......Y.LEG-VEL.GA..T......I....L..P........M...V.V........... 80

*M. sexta* -------.N.QS...Y-H..Q.EA.K.AGA.E..G...T.D....M....L..P..............I...F...V... 80

*B. mori* ---------------------------AGAVE..G...S.D.........LTFP..............I.......L... 80

*V. cardui* LVVFMFATTKSLRTSSNLLILNLAIFDFIMMAKAPLFIYNSAMRGFATGALGCQIFAVMGSYSGIGAGMTNACIAYDSHS 160

*E. chalcedona* ..IY.....................L..M......I.........Y...VM.....S..........A............ 160

*N. antiopa* ..M...S..................L..M.....................M......L...................... 160

*L. arthemis* ..M.V....................L..M......I.....................L...................... 160

*L. archippus* ..M.V....................L..M......I.....................L...................... 160

*D. plexippus* M.M.I.L..................S.........I.M....L....A.PV...M.S...A......S............ 160

*D. gilippus* M.M.I.M.................MS...........M....L....A.D....M..L.......V.S............ 160

*H. erato* ..I.....SS..S......................I....................S..........S............ 160

*H. melpomene* ..I.V..............................F...............................S............ 160

*A. vanillae* ..I.........S...........M..........I....................S..........S............ 160

*S. mormonia* ..I.I.....I.S......................I....................S...A......AT........... 160

*C. tullia* ..M...S...C.S...........ML..M..L...V....A.N....S.V....M..L...................... 160

*B. anynana* ..M...S.................ML..L......I......N....S.V....M..L...................... 160

*N. ridingsii* ..M...?.....S...........ML..M......I.....VNS.....?....M..L...................... 160

*O. chryxus* ..M.........S...........ML..M......I.....INK...A......M......................... 160

*L. rubidus* ..M..................Q..ML..M........M...........V....M..L...............M...... 160

*L. helloides* ..M..................Q..ML..M........M................M..L...............M...... 160

*L. heteronea* ..M..................Q..ML..M........M................M..L...............M...... 160

*L. nivalis* ..M..................Q..ML..M........M................M..L...............M...... 160

*S. behrii* ..IYI................Q...L...........M................M..L...............M...... 160

*A. glandon* ..M.I................Q..ML..M......I.....M........M..KM..L...................... 160

*P. icarus* ..M.I................Q..ML..M......I.....M........M..KM.GL...................... 160

*A. mormo* ..I..................Q..ML..M..L...M.............?M...M.G....................... 160

*C. philodice* M.I....S......A.........M......L...M.M.........A...W.KM.........M..A............ 160

*P. rapae* M.M.I..S......A.........M......L...M.......K...A..MW..M......................... 160

*P. glaucus* ..M.I.SAS.....P....VVQ...L..L..L...M.....MK....A.VM...M..F...V..TA..L....M...... 160

*P. xuthus* ..M...SAS.....P....VVQ..VL..L..L...M.....MK....S.VI...M..F...V..TA..L....M...... 160

*M. sexta* M.M...S..........F.V....ML..M......M...........V.TV...M..L..A............M...... 160

*B. mori* ..I.V.S.....S........Q..ML..M......M.............TI.....S...A...M............R.. 160

*V. cardui* TMTRPLDGRLSRGKALLMMALVWIYATPWSLMPLFKVWGRFVPEGYLTSCTFDYLSNTFDTKLFVACMFVCSYVFPMSFI 240

*E. chalcedona* ..................V.IM.M...........Q....Y..............T......P..?.I...........M 240

*N. antiopa* ....................IM.......................................................... 240

*L. arthemis* .I......S...........C..................................T...........V.T.......... 240

*L. archippus* .I......S...........C..................................T...........V.T.......... 240

*D. plexippus* .I.........Q........F..M.......L.......S.............................T.......TM. 240

*D. gilippus* .I.........Q........C..M.......L.......S..................S..........T........M. 240

*H. erato* .I......S...........C........A.....N.............................G.I.T...L...C.. 240

*H. melpomene* .I......S...........C..............N...................T.........G.I.T...L...G.. 240

*A. vanillae* .I.S....S...........C..............N...................T.........G.I.L....L..... 240

*S. mormonia* .I......S...........C........A.....S...................T.........G.I.........LL. 240

*C. tullia* ....................C..........L....I....A.............T.........G.......C...... 240

*B. anynana* ..................I.CI.M......ML..L.I..S..........S....T.S.......G.......C...F.M 240

*N. ridingsii* ..............V.....CM.........L.......................T....N....G.............M 240

*O. chryxus* ....................CM..............I..S...............T.........G.............M 240

*L. rubidus* ..............VM....CM.........L........Y..............TD..........I..........M. 240

*L. helloides* ..............VM...TFM.........L........Y..............T...........I..........M. 240

*L. heteronea* ..............VM....CM.........L........Y..............T...........I..........M. 240

*L. nivalis* ..............VM....CM.........L........Y..............T...........I..........M. 240

*S. behrii* ...S..........VM....CM.........L....I...Y..............T.S.......G.I..........L. 240

*A. glandon* ...........Q...I..V.CM.............E....Y........................G.I.L.......TM. 240

*P. icarus* ...........Q...I..V.FM.............E....Y........................G.I.L.......TM. 240

*A. mormo* ........S.....VMM...FI.M.......L........Y..............T........................ 240

*C. philodice* .I.S.......S..V....ICM.M.T.........S...S...............T.S..N....G...T.......LC. 240

*P. rapae* ...S.......S..V.....FM...T.........S.........F.........TT...N.................C. 240

*P. glaucus* .I............V....VC..V.TA..AIL.QLQI...Y....F.........TT...N.....S....V.....LA. 240

*P. xuthus* .I............V....VC..L.TA..AIL.QLQI...Y....F.........TT...N.....S....V.M...IA. 240

*M. sexta* .I......S..E..V...V.F..M.S...A.L..L.I..SY.........S....T.............T........LM 240

*B. mori* .I......S..K..V.....F..M.C...A.L..L.I..SY..............T...........I..........MM 240

*V. cardui* MYFYSGIVKQVFAHEAALSEQAKKMNVESLSSNQNASAESAEIRIAKAALTVCFLFVASWTPYGVMSLMGAFGDQQLLTP 320

*E. chalcedona* I.............................R............................................R.... 320

*N. antiopa* I...........................................M..............................R.... 320

*L. arthemis* I.............................R.....A.....V.M..............................R.... 320

*L. archippus* I.............................R.....A.....V.M..............................R.... 320

*D. plexippus* ..............................R...........M.......................A........R.... 320

*D. gilippus* ..............................R...........M..............S........A........R.... 320

*H. erato* ..............................R.............M................................... 320

*H. melpomene* ..............................R.............M................................... 320

*A. vanillae* ........S.....................R.............M................................... 320

*S. mormonia* ..............................R.............M..............................R.... 320

*C. tullia* ........S..................D..............M.M....................TA.I......S.... 320

*B. anynana* L.............................R.....A......SM.....................A....Y...N.... 320

*N. ridingsii* L.............................R............SM..............................S.... 320

*O. chryxus* L.............................R............SM..............................S.... 320

*L. rubidus* I.....M....................D..R.....A.......M.....................A.I......S.... 320

*L. helloides* I.....M....................D..R.....A.......M.....................A.I......S.... 320

*L. heteronea* I.....M....................D..R.....A.......M.....................A.I......S.... 320

*L. nivalis* I.....M....................D..R.....A.......M.....................A.I......S.... 320

*S. behrii* I..........................D..R.....A.......M.....................A.I......S.... 320

*A. glandon* ........R..................D..R.....G......SM.....................A........N.... 320

*P. icarus* ........R..................D..R.....G......SM.....................A........N.... 320

*A. mormo* I.....M.............................G.......M............S........A........N.... 320

*C. philodice* I.............................R.....G.....M.........................I......E.... 320

*P. rapae* I.................R...........R.....A......S.............T.................N.... 320

*P. glaucus* ...........................D...........................Y............I......N.... 320

*P. xuthus* L..........................D........A..................Y............I......N.... 320

*M. sexta* ...............................A..GG.S.....SM.....................A......N...... 320

*B. mori* I.............................RA..SGASQ...MSM.....................A............. 320

*V. cardui* GVTMIPAVTCKLVACIDPWVYAISHPKYSQELQSRMPWLQINEPDDNASTGTNNTANSS-APA-TA 386

*E. chalcedona* ........A..T.....................K.........................-...AS. 386

*N. antiopa* ...........V...............................................-...-.. 386

*L. arthemis* ........A..A...M.................R......M..............T...-.T.AS. 386

*L. archippus* ........A..A...................P.R..S...M..............T...-.T.AS. 386

*D. plexippus* ....M...A..T.....................R.............V.NT..G.T..TA..--.. 386

*D. gilippus* ........A..A...M.................R.............V.NT..G.T..TA..--.. 386

*H. erato* ........A..T.....................RS.....MS.............T..T-...AS. 386

*H. melpomene* ........A..T.....................RS.....MS.............T..T-...A.. 386

*A. vanillae* ...........A.....................R......MR.....V.......T..T-...A.. 386

*S. mormonia* ........A..A.....................R......M..............T...-...AS. 386

*C. tullia* .......L...A.....................R.............V.......T..-A..--.. 386

*B. anynana* ...........A...M.................R......M...........T..T..T-T.--.. 386

*N. ridingsii* .................................R..........S..V....T..-.K.A..--.. 386

*O. chryxus* ...........A.....................R..................T..T...-..--.. 386

*L. rubidus* ....M...A..A.....................R......M...S.T.....T..T..T-...AS. 386

*L. helloides* ....M...A..A.....................R......M...S.T.....T..T..T-...AS. 386

*L. heteronea* ....M...A..A.....................R......M...S.T.....T..T..T-...AS. 386

*L. nivalis* ....M...A..A.....................R......M...S.T.....T..T..T-...AS. 386

*S. behrii* ....M...A..A.....................R......M...S.T.....T..T..T-...AS. 386

*A. glandon* ....M......A.....................R......M...S.T.....TA.-..-A...-S. 386

*P. icarus* ....M......A.....................R......M...S.S.....TA....-A...-S. 386

*A. mormo* ...........A.....................K.............T.NAS.A....-A..PAS. 386

*C. philodice* .......M...A...M.................RS......S.......NTS.T.T..-A...-W- 386

*P. rapae* .......M...A..........M...........S............T..TS.T.T..-A...-W- 386

*P. glaucus* ....M..LA..G.....................K.......D.....V.NT........-...--- 386

*P. xuthus* ....M..LA..G.....................K.......D.......NT.S......-...--- 386

*M. sexta* ........A..A....S.........M......R.......D....TV..A.S..T..-AP..A.. 386

*B. mori* ....M...A..A...M.................R............TT..A.S..VS.-AP..AP. 386

**Additional File 2b.** Alignment of 35 lepidopteran BRh complete coding sequences.

*V. cardui* -MATNYTDDIG---PVAYPLKMVTQEVVEHMLGWNIPEDHQDLVHEHWSNFPAVSKYWHYGLAFIYTILMLASVSGNGIV 80

*E. chalcedona* -..........---.A.......S..............E.........R..........F...C..S....T.L....M. 80

*N. antiopa* -.......?..---.....................................................M...?.L...... 80

*L. arthemis* -..........---.........SK....N........E..E.................IC..L......V..M...... 80

*L. archippus* -..........---.........S....QN........E..E............N....IC..L..S...I..M...... 80

*D. plexippus* -....F.E...---.M...M...S..............E.........R..........F.......L.....MT..... 80

*D. gilippus* -....F.E...---.M.......S..............E.........R..................L.....MT..... 80

*H. erato* -..........---.M.......S..............E..G..........S....A.FV..L...C.....L...... 80

*H. melpomene* -..........---.........S..............E..................A.FV..L..SF............ 80

*A. vanillae* -..A.......---.........S..............E..E...............A.FT......L..I......... 80

*S. mormonia* MA.S...E...---...H...L.S..............E....................I...L..SL..I..M...... 80

*C. tullia* -....F.....---...W.........E..........E..A......RD.............M...V..I..CL..... 80

*B. anynana* -......E...---...W.....S..............E......D...D.........F...L...V..F..M...... 80

*L. rubidus*1 -..Y.F...F.---...H.....SS.AE..........EY.YF.....................M..L..C..CL..... 80

*L. rubidus*2 -.EG.F..NM.---.L...FQ..SK.TE..V....Y..EY.FM.QD...SY..I.............M.F....T..... 80

*L. helloides1* -------------------------------------------------.....................C..CL..... 80

*L. helloides*2 -.EG.F..NM.---.L...FQ..SK.TD..V....Y..EY.FM.QD...SY..I.............M.F...IT..... 80

*L. heteronea*1 -..Y.F...F.---...H.....SS.AE..........EY.YF.....................M..L..C..CL..... 80

*L. heteronea*2 -.EG.F..NM.---.L...FQ..SK.TD..V....Y..EY.FM.QD...SY..I.............M.F...IT..... 80

*L. nivalis*1 -..Y.F...F.---...H.....SS.AE.........DEY.YF.....................M.....C..CL..... 80

*L. nivalis*2 -.EG.F..NM.---.L...FQ..SK.AD..V....Y..EY.FM.QD...SY..I.............M.F...IT..... 80

*S. behrii*1 -..Y.L.E.F.---..G......SS.TE.........DEY.YF.......Y.............M..M..C..CL..... 80

*S. behrii*2 -..G.F..NL.---.L...FQ...K.TD..V....Y..EY.FM.Q...RSY..I.............M.F...IT..... 80

*A. glandon*1 -..Y.L...F.---...-A....SD.AE.......V..EY.YF..D..RAY.....W.......M..M..FC.CL...M. 80

*A. glandon*2 -.D-.S.EN..---.T..AFQ.IEE.TK.NI..Y.Y..EY.FM.QD...SY..IN.....S..L...M.FI..IT..... 80

*P. icarus*1 -..F.L...F.---...-A....SD.AE.......V..EY.YF..D..RAY.....W.......M..M..FC.CL...M. 80

*P. icarus*2 -.D-.S.GN..---.M..AFQ..EE.TK..I..Y.Y..EY.FM.QD...SY..IN.....S..L...M.FI..IT..... 80

*A. mormo* -..M.F..G..---.L.L..Q.MA..AE.......M..E..H..Q....SY............CM.AL.W....T..... 80

*C. philodice*V -..E.F.ENE----.I.F.Y...SH.IQK......M..E..H......RQ..D.DRSS..L..LM....TMS..T...L. 80

*P. rapae*B -DTV.A.A.G.---AI..AF...SS..Q.N...F...PE.........R.....D.F...M..L...M..VS.LC..... 80

*P. rapae*V -.EL...AGD----.I.F.F...SG..QQ........AE..G......RQ....D.FS..L..L..F...IF..T..SL. 80

*P. glaucus* -..A..S....---.M...M.L.SS.I....M......E..AM..A..RS.......Y.FI..L...M..VT.LI..... 80

*P. xuthus* -..A..S....---.M...M.L.SS.M....M......E..AM..A..RS.......Y.FI..L...M..VT.LV..... 80

*M. sexta* -....F.QELYEIG.M......MSKD.A..........E......D..R...........V..LM..M..VT.LT..... 80

*B. mori* -..L.F.QEMSDIG.M.......SS.M...........E.............S.......C..LM.AM..VT.LV..... 80

*V. cardui* MWIFSTSKSLRSASNMFVINLAVFDLMMMLEMPMLVVNSFYQRLLGYQLGCDMYAVLGSLSGIGGAMTNAIIAFDRYKTM 160

*E. chalcedona* ...................................IS......P..................Y....G............ 160

*N. antiopa* .................................L..T........................................... 160

*L. arthemis* I.M...................I..M..........M.......V.........................V......... 160

*L. archippus* I.M...................I..M..........M.......V.............F...........V......... 160

*D. plexippus* I.M.........P.........I..F.......L..L...H.S.......................I...V........I 160

*D. gilippus* I.M.........P.........M..F...F...L..L...H.S.......................I...V........I 160

*H. erato* I.M...............M......M.......L..L.......V.........G...........I...V..Y.....I 160

*H. melpomene* I.M........T......M......M...M......L.......V........F............I...V..Y.....I 160

*A. vanillae* I.....................A..V.......L.II.....HPI.F.......G...........I..VV..Y.....I 160

*S. mormonia* I.M....................................Y.......R....L.G.F........SI............. 160

*C. tullia* ..M.......................T......L..I...H..PV.........G...........I..V...Y...... 160

*B. anynana* I.M...................M......I...L..I...H.S.V.....................I..V...Y...... 160

*L. rubidus*1 I.........S.P.........L..TL..F...L.I......KM.....S..I..SF.AM...................I 160

*L. rubidus*2 I.......A...P............V........FIL...HH.IV...TV..I..T...M..F...I...V..Y.....I 160

*L. helloides1*  I.........S.P.........L..TL..F...L.I......KM.....S..I..S..AM...................I 160

*L. helloides*2 I.......A...P............V........FIL...HH.II...TV..I..T...M..F...I...V..Y.....I 160

*L. heteronea*1 I.........S.P.........L..TL..F...L.I......KM.....S..I..SF.AM...................I 160

*L. heteronea*2 I.......A...P............V........FIL...HH.II...TV..I..S...M..F...I...V..Y.....I 160

*L. nivalis*1 I.........S.P.........L..TL......L.I......KM.....N..I..S...M...................I 160

*L. nivalis*2 I.......A...P............V........FIL...HH.II...TV..I..T...M..F...I...V..Y.....I 160

*S. behrii*1 I...........P.........I..TL..F.....IA.....KM.....S..I..SF.A...M................I 160

*S. behrii*2 I...........P............V...I...IF.L...H.HII...AV.NV..T...I..F...I...VM.Y.....I 160

*A. glandon*1 I...........P..F..M...M..TL..F.....I......TM.....S.....AF.AM..M....Q...M........ 160

*A. glandon*2 ........A...P.....M......V.......IF.L..YHHHMV...VV.NV..T...M..F...I...V..Y.....I 160

*P. icarus*1 I...........P..F..M...M..TL..F.....M......TM.....S.....AF.AM..M....Q...M........ 160

*P. icarus*2 ........A...P............V.......IF.L..YHHHMI...AV.NV..T...M..F...I...V..Y.....I 160

*A. mormo* I...........P.........L..T..A...........H..MV..ET..II.G...............V......... 160

*C. philodice*V ..M........TP.........L...A..G...H.IL.....SM...E....I.................VM.Y...... 160

*P. rapae*B V................IV...L...V..M...H.IM...F.KM...........T...V....A.I...V..Y.....I 160

*P. rapae*V I................IV...I...T......H.M......TMI.N.M...I...F.A.......I...V..Y.....I 160

*P. glaucus* I.M..........................I...L.IA.....HPI.F.....I......M......I...V........I 160

*P. xuthus* I.M...........S..............I...L.MA.....HPI.F.....V......M......I...V........I 160

*M. sexta* I.M...............M..............L.IM.......V.......V.............I...V........I 160

*B. mori* I.M.G.................M..........L.II.......V.....................I...V........I 160

*V. cardui* SSPLDGSLNRVQASLLILFSWLWALPFTFLPAFSVWGRYVPEGFLTTCSFDYMTDDQDTKIFVMCIFVWSYVIPMTFICC 240

*E. chalcedona* .................................KI...F.............L.E.....M.T....I...L..LM...F 240

*N. antiopa* .................................K....F.....................M..I.M.I.......V...F 240

*L. arthemis* .............A...A.T.F......I....K...............LTTLPE.....V....M......V..L...F 240

*L. archippus* .............A...A.T.F...........K...............LTTL.E....SV....M.I.......L...F 240

*D. plexippus* ......R..HT..A.....T.V..T..S.........K.............FL.E.E..RV..............CC..Y 240

*D. gilippus* ......R...T..A.....T.V..T..S.........K.............FL.E.E..RV....M.........CC..Y 240

*H. erato* ...................T........I.....I...F.............F.E.....V....M.T...C...LL?.Y 240

*H. melpomene* .........S.........T........IM....I...F.............F.E.....V....M.A...C...LLL.Y 240

*A. vanillae* ........T.T........T....V.............F.............F.E.....V..L.M.A.......L...Y 240

*S. mormonia* ........T..........T.M......L.........F.............I.E....SL....M.I.......L.M.Y 240

*C. tullia* ........T.M..Y....LT.I...........QI..K.............F....T..SV....M.M.......M.M.F 240

*B. anynana* ........T.......M..T..............I..KF.............F.E.T...L....M.I...A...LSM.F 240

*L. rubidus*1 .C....RITK...LI..A...V.SM........K....FM............L...P...L......C...M...I.L.F 240

*L. rubidus*2 .C.....VTKT..L.....T.V......I....KL.SKF.............L.E.S...V...SCC....F..VIIL.Y 240

*L. helloides1* .C....RITK...LI..A...V.S.........K....FM............L..EP...L......C...M...I.L.F 240

*L. helloides*2 .C.....VTKT..L.....T.V......I....KL.SKF.............L.E.S......LSCC....F..VIIL.Y 240

*L. heteronea*1 .C....RITK...LI..A...V.SM........K....FM............L.......L......C...M...I.L.F 240

*L. heteronea*2 .C.....VTKT..L.....T.V......I.....L.SKF.............L.E.S...V..LSCC....F..VIIL.Y 240

*L. nivalis*1 .C....RITK...LI..A...V.S.........K..S.FM............L..EP...L......C...L...I.L.F 240

*L. nivalis*2 .C....RVTKT..L.....T.V......I....KL.SKF.............L.E.S......LSCC....F..VIIL.Y 240

*S. behrii*1 .C.....ITK...LI.MA...V.S....L.........FM............L..HP...L......C....M..I.L.F 240

*S. behrii*2 .C.....VTKT..L..M..T.V......V....QL.SKF.............L...N......LSCC....F..I.IL.Y 240

*A. glandon*1 ......RITN...MI..V.T.I.T..........I...FM............L.E.T...V......L...AT..M.L.F 240

*A. glandon*2 .C....RVTKS..LA.MMMT.M.S....M....R...KF.............L.E.SA.RM..LVCCM.D.FL.IFML.Y 240

*P. icarus*1 ......RITN...MI..V.T.I.T.......F.R....FM............L.E.T...V......L....T....L.F 240

*P. icarus*2 .C....RVTKS..LA.MVMT.V.S....M...IQ...KF.............L.E.YA.RM..LVCCM.N.FL.VFML.Y 240

*A. mormo* .C.....M.K...I..CV.T....M.......LD....F.............L...T.I.V......M....M..L.L.T 240

*C. philodice*V ...I..RI.SA..T..VM.T..Y.M...VF.LTKT..SF.T..........FLS..VS.--..L.MSI........L..T 240

*P. rapae*B .C.I...I.K...II..V.T.F.T....I..LTR...KF............SF...S..RV..A...........VL..F 240

*P. rapae*V .C.I..RI.K....I..AVT.M......I..WTR...Q.TT..........FLSE.....A..A.M.C........LM.T 240

*P. glaucus* .C.....M.K.......V.T.F.S....M...LK....F.............F.......V..A.......A...AL..Y 240

*P. xuthus* .C.....I.K.......A.T.F.SM...I...LK....F.............F.......V..A.......A...AL..Y 240

*M. sexta* .......M.T...G...A.T.F......I.....M..SF.............F.E....EV..A.......CM..AL..Y 240

*B. mori* .C.....I.K......MA.T.F......I.....M.................F.......V..A.......C....LM.Y 240

*V. cardui* FYSKLFGAVRLHERMLKEQAKKMNVKSLAANKEDSGKSIEMSIAKVAFTIFFLFLYSWTPYAFVTMTGAFGDSGLLTPVA 320

*E. chalcedona* ......S...........................A...V.I.............VCA.......S........S.....T 320

*N. antiopa* ..............................G...A...V................C........................ 320

*L. arthemis* ..............................G...A...V.I.............VC..............E..SI...LT 320

*L. archippus* ..............................G...A...V.I.............VC........A........SI...LT 320

*D. plexippus* ......S.........................D.....V.IR.......M....VCA.......A........SI..... 320

*D. gilippus* ......S.........................D.A...V.IR.......M....ICA.......A........SI..... 320

*H. erato* ..................................A...V...............VC.................SI..... 320

*H. melpomene* ..................................A...................VC........A.V......SI....T 320

*A. vanillae* ..................................A...V...............VC........................ 320

*S. mormonia* ..................................A...................VC........A............... 320

*C. tullia* ......S.......................G...A...V.I.............VC........A.M......S...... 320

*B. anynana* ......S.......................G...A...V.I.............VC........A.I......SM..... 320

*L. rubidus*1 ......S...M..K..R............S..D.A...V.I.............VC.................NI..... 320

*L. rubidus*2 Y.FQ......T..K..R............S....G...V.I.............ICA........LV......SI.S... 320

*L. helloides1* ......S...M..K..R............S..D.A...V.I.............VC.................NI..... 320

*L. helloides*2 Y.FQ......T..K..R............S....G...V.I.M...........ICA........LV......SI.S... 320

*L. heteronea*1 ......S...M..K..R............S..D.A.T.V.I.............VC.................NI..... 320

*L. heteronea*2 Y.FQP.....T..K..R............S....G...V.I.M...........ICA........LV......SI.S... 320

*L. nivalis*1 ......S...M..K..R............S..D.A...V.I.............VC.................NI..... 320

*L. nivalis*2 Y.FQ......T..K..R............S....G...V.I.M...........ICA........LV......SI.S... 320

*S. behrii*1 ......S...M..K..R............S..D.A...V.I.............VC.................NM.S... 320

*S. behrii*2 Y.FQ......N..K..R...........SS....G...V.I.............ICA........LV......SI..... 320

*A. glandon*1 ......N...A..S..R............S..DEA.T.V.IR.......M....VC................K.I..... 320

*A. glandon*2 Y..R......T..K..R...........-S..D.G.A.V..RM...........VCA........LV......SI..... 320

*P. icarus*1 ......N...A..S..R............S..DEA.T.V.IR.......M....VC................K.I..... 320

*P. icarus*2 Y.AL......T..K..R...........-S..D.G.A.V..RM.........M.VCA........LV......SI..... 320

*A. mormo* ......?......N...............S....A...V.I.............VC..........N......SI..... 320

*C. philodice*V ..L.......H..K...............S....A...V.I.............IC.....GI.A.I......S.....V 320

*P. rapae*B ......S......K..R............S..D.A...V.I.............VCA.....V...I.T....N....HV 320

*P. rapae*V ......V...H..S...............S....A...V.I.M...........IC.....GV...I......S.....V 320

*P. glaucus* ...Q.....S...K..Q............S....AS..V.............M.VCG.....I......Y...S..S... 320

*P. xuthus* ...Q.........S..Q............S....AS..V.I...........M.VCG............Y...S...... 320

*M. sexta* ...Q............Q............S....NSR.V.I.M...........ICA................T....M. 320

*B. mori* ...Q............Q.................ASS.V.I.M......M....VCA................N....M. 320

*V. cardui* TMVPAVCAKIVSCIDPWVYAINHPSYSAELQKSLPWMGVREADPDSVSSA-SGATAQTQNPTAE- 385

*E. chalcedona* ........................K.......RV........NS.T..TS-.............A 385

*N. antiopa* ..................................................-........S....A 385

*L. arthemis* ...........................V....R................T-........HAA..A 385

*L. archippus* ...........................V....R................T-........HAA..- 385

*D. plexippus* ..M......V...L..................RV...........NT.NV-........-NP..A 385

*D. gilippus* ..M......V...L..................RV...........NT.NV-........-NP..A 385

*H. erato* ..M.............................R...............TS-.............A 385

*H. melpomene* ..M.............................R............T..TS-.............A 385

*A. vanillae* ................................R...............TS-.A......HAN..A 385

*S. mormonia* .........V......................RV...........T..TS-.........AA..A 385

*C. tullia* .............M............R.....RV.......P......N.-....TH..H.S.DA 385

*B. anynana* .............M............R...E.RV.......P......TS-........-NA..A 385

*L. rubidus*1 .............M................E.RVS.L..K.PN..T..TS-.T..S.---AP.DA 385

*L. rubidus*2 .........T...M................T.R...L....S...TA..S-.T..S...HH...A 385

*L. helloides1* .............M................E.RVS.L..K.PN..T..TS-.T..S.---AP..A 385

*L. helloides*2 .........T...M................T.R...L....S...TA..S-.T..S...HH...A 385

*L. heteronea*1 .............M................E.RVS.L..K.PN..T..TS-.T..S.---AP..A 385

*L. heteronea*2 .........T...M................T.R...L....S...TA..S-.T..S...HQ...A 385

*L. nivalis*1 .............M................E.RVS.L..K.PN..T..TS-.T..S.---AP..A 385

*L. nivalis*2 .........T...M................T.R...L....S...TA..S-.T..S...HH...A 385

*S. behrii*1 .............L................E.RVS.L..K.PN..T..TS-.T..S.....Q..A 385

*S. behrii*2 ..M......T...M................T.R...L....Q...TA..S-.T......HQ...A 385

*A. glandon*1 ......A.......................E.RVS.L..K.PS..T..QS-.T..S.---VPQ.A 385

*A. glandon*2 .........T..............K.....T.R...L....K...TA..S-.T..S...H....A 385

*P. icarus*1 ......A......M................E.RVS.L..K.PS..T..QS-.T..S.---VPQ.A 385

*P. icarus*2 .........T..............K.....T.R...L....K...TA..S-.T..S..HH....A 385

*A. mormo* ..............................E.RI.......PNH.T..TS-.T..S...HAAT.A 385

*C. philodice*V ..I...FC.A...L...........F.V..E.RV.....S.P...AQ..T-GS.VTN----S..A 385

*P. rapae*B ..I...F..S....................E.......I..PSAETQ.TN-ASTAT.S--AS.DA 385

*P. rapae*V ..I...FC.A...............FR...ESRV.......P...AT.TN-ASTSTT----P.DA 385

*P. glaucus* ..I....C.....M..........R.......R...L....Q......TSN.VT.T.SHT.N..T 385

*P. xuthus* ..I....C.....M..................R...L....Q...T..NSN.VT.T.SHT....A 385

*M. sexta* ..I....C.V...M..................R........Q...A..TTT.V...GF.P.A..A 385

*B. mori* .......C.V...M................T.R...L....S...A..TTT.VG...S.-A...A 385

**Additional File 2c.** Alignment of 35 lepidopteran LWRh complete coding sequences.

*V. cardui*  MAITSLD--PGAAALQAWGGQMAAFG-SNETVVDKVLPDMLHLVDPHWYQFPPMNPLWHGLLGFVIGILGFISITGNGMV 80

*E. chalcedona* .......LG...............Y.-.....?..AP.E....I........?...M.Y.....FM.?...?.V...... 80

*N. antiopa* .......--.................-...........E......................................... 80

*L. arthemis* .......PG...............Y.-....................................L...V...V..A..... 80

*L. archippus* .......PG...............Y.-...........................D........L.M.V...?..?..... 80

*D. plexippus* .....M.PG.................-................I............M........M.V...M........ 80

*D. gilippus* .....M.PG...T.......E.....-................I............M........M.V.S...V...... 80

*H. erato* .......PG.........?.......-................I....H..................V.....V...... 80

*H. melpomene* .......PG.................-................I.A..H..................V.....V...... 80

*A. vanillae* .......AG.............V...-................I.......................V.....V...... 80

*S. mormonia* .......PG.................-................I.......................V.....V...... 80

*C. tullia* ....NM.PG..V....G.E..AM.Y.-..M..L..AT......M..Y...........Y......MVV.AV...C..... 80

*B. anynana* .....M.PG..I........HA.?Y.-........................................V...V........ 80

*N. ridingsii* .....M.PG..V..M......AMP..-...?...................Y........S.....M.V......A..... 80

*O. chryxus* .....M.PG..V..M......AM.Y.-................I......Y............L.M.V.....FA..... 80

*L. rubidus* .S.....PA..V..M....P.AM.Y.-G....I.....E...KI.A................A...SMICI.AT...... 80

*L. helloides* .S.....AA..V..M....P.AM.Y.-G....I.....E...KI.A............Y...A..MS.ICV.AT...... 80

*L. heteronea* .S.....PA..V..M....P.AM.Y.-G....I.....E...KI..................A...S.ICL.AT...... 80

*L. nivalis* .S.....PA..V..M.G..PRAM.Y.-G....I.....E...KI.A................A...STICL.AS...... 80

*S. behrii* .T.A...PA..V..M......AL...-...........E...KI................I.A.I.SVV.I.AT...A.. 80

*A. glandon* .T.MN..PA..V....S..P.AS.LF-N..........E...SI................I.A.ITSVILM.AS...... 80

*P. icarus* .T.MN..PA..V....S..PRAS.LF-N...G.....SE...SI....D..........AI.A.ITSVILM.AS...... 80

*A. mormo1* .T..N..PG.RF.PIE.-----L...-...........E....I.....E..............FMACITI.AFA..... 80

*A. mormo2* ....N..PG..V....S..P.TM..S-N.M........E...KI.K.........D..Y..V.?.MAWICMTAFS..A.. 80

*C. philodice* .....M.PA..V..M.....HAE.YS-..Q........E....I.A............Y.....T.S..A...V...... 80

*P. rapae* ....N..PA..V..M.SF.IHAE...-..Q..M.....E.M..I.........L.....A....T.SV.A...M...... 80

*P. glaucus1* ..LD...PAAT--FGH..A.K.E.Y.-..Q..I.Q...E.M..I......................AV...M.LS..... 80

*P. glaucus2* ...AN..PGL..-.AEV....A...S-..Q......S...M..I............M.......T..V...M........ 80

*P. glaucus3* ..LDY.NTGAA--KMGT.N...S.Y.-A.Q........E....I..............Y.....T.AC.AIT.....A.. 80

*P. xuthus1* ..MD...PGAA--SAP..A.KIE.Y.-..H..I.Q...E....I......................AV...M.LS..... 80

*P. xuthus2* ...AN.EPGM.--.SE.....A....-..Q......T...M..I............M.......T..V...M........ 80

*P. xuthus3* ..LNY.NTGAA--KMDT.N...S.Y.-A.Q........E....I..............Y.....T.TC.AIT.....A.. 80

*M. sexta* .-----.PG..L......AAKSP.Y.AA.Q......P...M.MI...............A....T..V...V.MS..... 80

*B. mori1* .-SM.M.AG..F....S.SS.V....N..Q....S.S.E....I.AY............A....T..V.....MM..... 80

*B. mori2* .--I...PG..M.........V..Y.AA.Q......P.....M................A....T..V...M..S..... 80

*V. cardui*  IYIFTTTKSLKTPSNILVVNLAFSDFLMMCVMSPPMVVNCYTETWVFGPLACQLYACAGSLFGCASIWTMTMIAFDRYNV 160

*E. chalcedona* V...............F.........?LLGL.A........Y......................V............... 160

*N. antiopa* V.......T................................N......................V............... 160

*L. arthemis* V.......T.................C..LF.A........Y...................................... 160

*L. archippus* V.......T.................C..L.......MS..Y...?...?G......T...................... 160

*D. plexippus* V.......T.................C..AI.A...LI...N......................G............... 160

*D. gilippus* V............................AL.A...MI...N.......F..............V............... 160

*H. erato* V.......T....................FM.A....M...N...................Y..V............... 160

*H. melpomene* V............................FM.A....M...N...................Y..V............... 160

*A. vanillae* V............................FM.A....M...H...................Y..V............... 160

*S. mormonia* V.......T....................AM.A........Y...................Y..V............... 160

*C. tullia* V.......T....................AL.A........Y...................L.................. 160

*B. anynana* V.................M..........L...........N......................T.......M....... 160

*N. ridingsii* V.......T......V.?...........T...........Y..................?L..V............... 160

*O. chryxus* V....?.......................T....A.....FY...................................... 160

*L. rubidus* ....S..........L......L....IITT....V...T.Y...M......DI...C......V............... 160

*L. helloides* ....S..........L......L....IITT....A...T.Y...M......DI...C......V............... 160

*L. heteronea* ....S..........L......L....IITT....V...T.Y...M......DI...C......V............... 160

*L. nivalis* ....S..........L......L....IITT....L...T.Y...M......DT...C......V............... 160

*S. behrii* ?...C...T......L......L....IITT....V...T.Y...M......DI...C......V............... 160

*A. glandon* ........T......LFI....L....IITT....V...T.Y...I......DI...C......V...S........... 160

*P. icarus* ........T......LFI....L....II?T....V...T.Y...I......DI...C......V...S........... 160

*A. mormo1* L..............L..M.......C.IV..G...L.S..Y..........E...........G...S........... 160

*A. mormo2* L...NS....S....LF.........A.I..LA..VL..S.YQ.......F.DI...C......V............... 160

*C. philodice* ...............L.............AM.A..LCI.S.YQ......V...F...F......V......A........ 160

*P. rapae* V..............L.............AM.A..L...S.N.......T...F...F......V......A........ 160

*P. glaucus1* ..M.....T......L..L...V......TC.A..L...S.H..........A...A......TI............... 160

*P. glaucus2* V....S.........L.............LC.A........Y..........E..........SM............... 160

*P. glaucus3* ........N......L......V......AC.A..LII.S.N........F.AI...G...Y.TV......A........ 160

*P. xuthus1* ........T......L..L...V......TC.A..L...S.H..........A...A......TI.......M....... 160

*P. xuthus2* V....S.........L.............LC.A...LI...Y..........E..........SM............... 160

*P. xuthus3* ........N......L......V......AC.A..LII.S.N........F.AI...G...Y.TV......A........ 160

*M. sexta* ....MS.........L..............A...A......Y....W..F..E...................M....... 160

*B. mori1* ....M...N......L..............A...A..I...N.......F..E..G........................ 160

*B. mori2* ....MS.........L..............A...A......N.......F..E........................... 160

*V. cardui*  IVKGIAAKPLTINGAMLRVLGIWVFSLAWTVAPLFGWGRYVPEGNMTACGTDYLDKSWFNRSYILIYSIFCYFSPLFLII 240

*E. chalcedona* ...........V...L...FA..M...L.....ML..........L.......F..G.L......L..V....L...... 240

*N. antiopa* .........M.............M.........M..................................V....M...... 240

*L. arthemis* .........M.....L.......A...S..I......................F..T.G......F...A..YM...... 240

*L. archippus* .........M.....L.......A.A.?..I......................F..T.V......V...A..YT...... 240

*D. plexippus* ....L....M.....L.......A.........M...................F...FA.....V...V....A...... 240

*D. gilippus* ....M....M.....L.......A......L......................F...VA.IT..VT...A...A...... 240

*H. erato* .........M.....L...F...A......I......................F.Q.FS......L...A..YA...... 240

*H. melpomene* .........M.....L...FF..A.............................F.Q.IS.MT...L...A..YA...... 240

*A. vanillae* .........M.....L...FF..A...G..L......................F...LS..T...L...A..YA...... 240

*S. mormonia* .........M.....L.S.F...L.............................F....S..T..ML..VA..YL....M. 240

*C. tullia* ...............L...F...M...I.....M....S...................VH.....V..V...YA...... 240

*B. anynana* ......G........L...FA..L......I.......S..............F....Q......F......Y...L..C 240

*N. ridingsii* .........M.....L.......L...G.....MM...S...................L......V..L...Y...L... 240

*O. chryxus* .........M.....L.......L.........M....S...................L......V..V...Y...L... 240

*L. rubidus* ...............L..I....L......IT......K.A.....CV..........VH....IL..VA...A?.L... 240

*L. helloides* ...............L..I....L......IT......K.A.....CV..........VH....IL..VA...A...... 240

*L. heteronea* ...............L..I....L......IT......K.A.....CV..........VH....IL..VA...A...... 240

*L. nivalis* ...............L.QI....L......IT......K.A.....CV..........VH....IL..VA...A...... 240

*S. behrii* .........M.N...L..I....L......LT......K.A.....CV..........VH....IL...A...A...... 240

*A. glandon* ...........NG..L..I..V.L.......T......K.A.....CV..........VH.?..IL..FA...M...... 240

*P. icarus* ...........NG..L..I....L......IT......K.A.....CV..........VH....IL..FAY..M...... 240

*A. mormo1* .........M.....L.SIF.M.L.............CS...................LS.....V....V..A..?... 240

*A. mormo2* ....M..........L..I....L.?....L..M....K.A..........NS..QALD?V?..WL..VA...L..G..C 240

*C. philodice* ...............L.QIFAV.A......L..I...SS...............S.DLLSQI..IT...A...L..A..V 240

*P. rapae* .........M...S.L.SI..V.L......L..I...S................S.D.AS....IL.A.A...L.....V 240

*P. glaucus1* .........M.N...L..I.A...S........M...NS...............N.D..S....VA.A.....T..A... 240

*P. glaucus2* .........M.....L..I....L......I..I...NS...............N...LS.....V....V.YM..L... 240

*P. glaucus3* .........MS....L..I.A..LS........I...NS........V......S.DMLS....IA.AV....L..G... 240

*P. xuthus1* .........M.N...L..I.A...S........M...NS...............N.D..S....VA.A.....T..A... 240

*P. xuthus2* .........M.....L..I....L......I..ML..NS...............S...LS.....V....V.YT..L... 240

*P. xuthus3* .........MS....L..I.A..LS........I...NS........V......S.D.LS....IA.AV....L..G..V 240

*M. sexta* ....M....M.S...L..M...........LL.F...NS...............S...VS........V.V..L..L... 240

*B. mori1* .........M.N...L..I....A.........F...NS...............T.D..S....VV..V.V..A..L..V 240

*B. mori2* .........M.N...L..I....A......L..F...NS...............S.D..S........V.V..A..L..M 240

*V. cardui*  YSYFFIVQAVAAHEKAMSEQAKKMNVASLS--SSDAANTSAECKLAKVALMTISLWFMAWTPYLVINYAGIFETATITPL 320

*E. chalcedona* ........?.....S...............--...................................F....D.I.L... 320

*N. antiopa* ..............................--................................................ 320

*L. arthemis* ..............................--...Q..................................M...M..S.. 320

*L. archippus* ......I.......................--...QG.....................................M..S.. 320

*D. plexippus* ......I.......................--...Q...............................FC...DG.P.S.. 320

*D. gilippus* ......I.......................--...Q...............................FC...DG.P.S.. 320

*H. erato* ....?.........................--........................................K.M..S.I 320

*H. melpomene* ..............................--...Q....................................K.M..S.I 320

*A. vanillae* ..............................--........................................D.M..S.I 320

*S. mormonia* ..............................--...Q......................................M..S.. 320

*C. tullia* ...T................?.........QQ.DADK.....................................M.MS.I 320

*B. anynana* ......I.......................--..EN......................................M..S.. 320

*N. ridingsii* ......M.......................--...Q................................S.....MQ.S.. 320

*O. chryxus* ..............................--...Q................................S.....MQ.S.. 320

*L. rubidus* ...W..I...S..................R--................................I..W....K.SL.S.. 320

*L. helloides* ...W..I...S..................R--................................I..W....K.SL.S.. 320

*L. heteronea* ...W..I...S..................R--................................I..W....K.SL.S.. 320

*L. nivalis* ...W..I...S..................R--................................I..W....K.SL.S.. 320

*S. behrii* ...W..I...S..................R--...G.DK.........................I..W....K.SM.S.. 320

*A. glandon* ...W..I...S..................R--...Q............................I..F....K.EL.S.. 320

*P. icarus* ...W..I...S..................R--...Q............................I..F....K.ER.S.. 320

*A. mormo1* ......I...S...................--...QS..............................FT.V....K.... 320

*A. mormo2* ......L...S......R...........R--...Q..............I.....T.......I..MS..-HG.GL... 320

*C. philodice* ..............................--..EQS...........................?..F..V....P.S.. 320

*P. rapae* ...W..........S...............--..EQ...............................F..V...SP.S.. 320

*P. glaucus1* ......I.......S...............--..E.................M..............FT........S.. 320

*P. glaucus2* ..............S..............R--..E.................................T.V....A.S.. 320

*P. glaucus3* ...W..I.......................--...................................F..V....P.S.V 320

*P. xuthus1* ......I.......................--..E................................FT........S.. 320

*P. xuthus2* .............................R--..E.................................T.V....P.S.. 320

*P. xuthus3* ...W..I.......................--...................................F..V....P.S.V 320

*M. sexta* ..............................--..E.................................T.V..S.P.S.. 320

*B. mori1* ...YY.....S...................--..E.....T...........................T.ML.S.P.S.. 320

*B. mori2* ..............................--..E..............................M..T.V..S.P.S.. 320

*V. cardui*  ATIWGSVFAKANAVYNPIVYGISHPKYRAALYASFPALACQPSP--EDNASVAS-AATA-TEEKPSA 387

*E. chalcedona* ............................................--........-....-....... 387

*N. antiopa* ............................................--........-....-....... 387

*L. arthemis* V.......................................A.E.--Q..T....-S...-....... 387

*L. archippus* V.......................................A.E.--Q..T....-S...-.?..... 387

*D. plexippus* ......................................S..A.S--D..V.A..-....C....... 387

*D. gilippus* ...........................................A--D..V.A..-....C....... 387

*H. erato* V...................................G....SA.--...G....-....-...?... 387

*H. melpomene* V.....................................S..TA.--..TG....-....-....... 387

*A. vanillae* V...................................G....SA.--...G....-....-....... 387

*S. mormonia* V...................................G.....AA--.E.G....-....-....... 387

*C. tullia* V....A...........................R..........GDS..T.T..-...H-....... 387

*B. anynana* V................................R..G....AAA--...G....-....-....... 387

*N. ridingsii* V................................R.........A--..T.....-....-....A.. 387

*O. chryxus* V.M..........C...................R........KA--D.T.....-....-....... 387

*L. rubidus* V.....I.....SI?...............?.....S.......--DESG....-TG..VQ...... 387

*L. helloides* V.....I.....SI..................?...S.......--DETG....-?G..IQ...... 387

*L. heteronea* V.....I.....SI......................S......S--DETG....-TG..IQ...... 387

*L. nivalis* V.....I.....SL......................S......S--DETG....-TG..IQ...... 387

*S. behrii* V.....I.....SI...................R.........A--DESG....-S...VQ...... 387

*A. glandon* .............I.......M...........R.........A--DESG....-SG..VQ...... 387

*P. icarus* .............I......DM...........R.........A--DESG....-SG..VQ...... 387

*A. mormo1* F...........TI......................S....?..--DESG....S.T..VQ...Q.. 387

*A. mormo2* S.....L....STI...................K........ES--DESG.I..T.?..---...A. 387

*C. philodice* S...............................Q...........S-.ETG....-....C....... 387

*P. rapae* S...............................Q...........A-.ETG....-....C....... 387

*P. glaucus1* G..........................S....Q...S.....AA--D..T.Q..-GK.TVC...... 387

*P. glaucus2* ................................QK..S.......--.ETG....-G..TAC...... 387

*P. glaucus3* S..........................S....QR..S.......--DESG....-GN..VC....P. 387

*P. xuthus1* G..........................S....Q...S.....AA--D..T.QV.-GK..VC...... 387

*P. xuthus2* ................................QK..S......A--.ETG....-G..TAC...... 387

*P. xuthus3* S..........................S....QR..S.......--DESG....-GN..VC...AP. 387

*M. sexta* ......L....................Q.....K..S.Q..SA.--..AG....-GT..VS....A. 387

*B. mori1* ......L....................Q....K...V.Q.HSTTT-DEAS....-G-.TVM....T- 387

*B. mori2* ......L....................Q........S.Q..SA.P-D.GG....-G...VS....A. 387

**Additional File 2d.** Alignment of 29 lepidopteran EF1α partial coding sequences.

*V. cardui* IDIALWKFETAKYYVTIIDAPGHRDFIKNMITGTSQADCAVLIVAAGTGEFEAGISKNGQTREHALLAFTLGVKQLIVGV 80

*E. chalcedona* ................................................................................ 80

*N. antiopa* ................................................................................ 80

*L. arthemis* ................................................................................ 80

*L. archippus* ................................................................................ 80

*D. plexippus* ............F................................................................... 80

*D. gilippus* ..........S.F................................................................... 80

*H. erato* ................................................................................ 80

*H. melpomene* ................................................................................ 80

*A. vanillae* ................................................................................ 80

*S. mormonia* ..........N..................................................................... 80

*C. tullia* ................................................................................ 80

*B. anynana* ................................................................................ 80

*N. ridingsii* ................................................................................ 80

*O. chryxus* -------......................................................................... 80

*L. rubidus* ................................................................................ 80

*L. helloides* ................................................................................ 80

*L. heteronea* ................................................................................ 80

*L. nivalis* ......................................R......................................... 80

*S. behrii* ................................................................................ 80

*A. glandon* ................................................................................ 80

*P. icarus* ................................................................................ 80

*A. mormo* ................................................................................ 80

*C. philodice* ..........S..................................................................... 80

*P. rapae* ..........G..................................................................... 80

*P. glaucus* ..........S..................................................................... 80

*P. xuthus* ..........S..................................................................... 80

*M. sexta* ..........S..................................................................... 80

*B. mori* ..........S..................................................................... 80

*V. cardui* NKMDSTEPPYNEGRFEEIKKEVSSYIKKIGYNPAAVAFVPISGWHGDNMLEASTKMPWFKGWQVERKEGKAEGKCLIEAL 160

*E. chalcedona* ..........S.S...................................................D............... 160

*N. antiopa* ................................................................................ 160

*L. arthemis* ..........S.S.................................................A........D........ 160

*L. archippus* ..........S.S.................................................A........D........ 160

*D. plexippus* ..........S.S......................................Q...........I................ 160

*D. gilippus* ........T.S.S......................................Q...........I..R............. 160

*H. erato* ............A..........................................................D........ 160

*H. melpomene* ............A..........................................................D........ 160

*A. vanillae* ..........S.S......................................P...................D........ 160

*S. mormonia* ..........S.S......................................P............D............... 160

*C. tullia* ..........S.P......................................P............................ 160

*B. anynana* ..........S.S..........................................................D........ 160

*N. ridingsii* ..........S.P......................................P............................ 160

*O. chryxus* ..........S.P......................................P............................ 160

*L. rubidus* ..........S.S................................................................... 160

*L. helloides* ..........S.P................................................................... 160

*L. heteronea* ..........S.P......................................P............................ 160

*L. nivalis* ..........S.P................................................................... 160

*S. behrii* ............S...................................................D............... 160

*A. glandon* ..........S.S................................................................... 160

*P. icarus* ..........S.S................................................................... 160

*A. mormo* ..........S.P......................................P............................ 160

*C. philodice* ..........S.S......................................P..........L................. 160

*P. rapae* ............S......................................P..........N................. 160

*P. glaucus* ..........S.S......................................P..........N................. 160

*P. xuthus* ..........S.S......................................P..........N................. 160

*M. sexta* ..........S.S......................................P..........L................. 160

*B. mori* ..........S.P......................................P...................D..S..... 160

*V. cardui* DAILPPARPTDKALRLPLQDVYKIGGIGTVPVGRVETGVLKPGTIVVFAPANITTEVKSVEMHHEALQEAVPGDNVGFNV 240

*E. chalcedona* ................................................................................ 240

*N. antiopa* ................................................................................ 240

*L. arthemis* ............................................V................................... 240

*L. archippus* ............................................V................................... 240

*D. plexippus* ................................................................................ 240

*D. gilippus* ................................................................................ 240

*H. erato* ................................................................................ 240

*H. melpomene* ................................................................................ 240

*A. vanillae* ................................................................................ 240

*S. mormonia* ............P................................................................... 240

*C. tullia* ............P......................................................S............ 240

*B. anynana* ................................................................................ 240

*N. ridingsii* ...................................................................S............ 240

*O. chryxus* ......................................I............................S............ 240

*L. rubidus* ..................................................................I............. 240

*L. helloides* ................................................................................ 240

*L. heteronea* ................................................................................ 240

*L. nivalis* ................................................................................ 240

*S. behrii* ................................................................................ 240

*A. glandon* ...................................................S............................ 240

*P. icarus* ...................................................S............................ 240

*A. mormo* ............P...............................V................................... 240

*C. philodice* ............P................................................................... 240

*P. rapae* ................................................................................ 240

*P. glaucus* ................................................................................ 240

*P. xuthus* ................................................................................ 240

*M. sexta* ............P................................................................... 240

*B. mori* ............P................................................................... 240

*V. cardui* KNVSVKELRRGYVAGDSKNNPPKGAADFTAQVIVLNHPGQISNGYTPVLDCHTAHIACKFAEIKEKVDRRSGKSTEDNPK 320

*E. chalcedona* ......................................................................T.....E... 320

*N. antiopa* ................................................................................ 320

*L. arthemis* ............................................................................E... 320

*L. archippus* ............................................................................E... 320

*D. plexippus* ............................................................................E... 320

*D. gilippus* ............................................................................E... 320

*H. erato* ..................S........L.................................................... 320

*H. melpomene* ................................................................................ 320

*A. vanillae* ............................................................................E... 320

*S. mormonia* ......................R......................................................... 320

*C. tullia* ............................................................................E... 320

*B. anynana* ......................................................................T........I 320

*N. ridingsii* ................................................................................ 320

*O. chryxus* ......................................................................T......... 320

*L. rubidus* ......................................................................T......... 320

*L. helloides* ............................................................................E... 320

*L. heteronea* ..G.........................................................................E... 320

*L. nivalis* ............................................................................E... 320

*S. behrii* ................T............................................................... 320

*A. glandon* ......................R.....................................................E... 320

*P. icarus* ................................................................................ 320

*A. mormo* ...........F..........R.....................................................E... 320

*C. philodice* ......................................................................T......... 320

*P. rapae* ..................S...................................................T......... 320

*P. glaucus* ......................R...............................................T......... 320

*P. xuthus* ......................................................................T......... 320

*M. sexta* ......................................................................T......... 320

*B. mori* ......................................................................T.....V... 320

*V. cardui* SIKSGDAAIVNLVPSKPLCVEAFQEFPPLG----- 355

*E. chalcedona* .....................S......------- 355

*N. antiopa* .....E.....................L..----- 355

*L. arthemis* ............Q.................RFAVR 355

*L. archippus* ............Q.................RFAVR 355

*D. plexippus* ..............................RFAVR 355

*D. gilippus* ..............................RFAVR 355

*H. erato* ............Q.................RFAVR 355

*H. melpomene* ............Q.................RFAVR 355

*A. vanillae* ............Q.................RFAVR 355

*S. mormonia* ..........I.Q.................RFAVR 355

*C. tullia* ..............................RFAV- 355

*B. anynana* .....................S....--------- 355

*N. ridingsii* ............................------- 355

*O. chryxus* ..............................RFAVR 355

*L. rubidus* .....................S........RFAVR 355

*L. helloides* .....................S........RFAVR 355

*L. heteronea* .....................S........RFAVR 355

*L. nivalis* .....................S........RFAVR 355

*S. behrii* .....................S........RFAVR 355

*A. glandon* .....................S........RFAVR 355

*P. icarus* .....................S........RFAVR 355

*A. mormo* .....................S........RFAVR 355

*C. philodice* .....................S........RFAVR 355

*P. rapae* .....................S........RFAVR 355

*P. glaucus* .....................S........RFAVR 355

*P. xuthus* .....................S........RFAVR 355

*M. sexta* .................M...S........RFAVR 355

*B. mori* .....................S........RFAVR 355

**Additional File 2e.** Alignment of 29 lepidopteran COI partial coding sequences.

*V. cardui* RMNNMSFWLLPPSLMLLISSSIVENGAGTGWTVYPPLSSNIAHSGSSVDLAIFSLHLAGISSILGAINFITTIINMRVNS 80

*E. chalcedona* ...............................................................................N 80

*N. antiopa* ..............I..............................A...............................I.N 80

*L. arthemis* ------------------A............................................................G 80

*L. archippus* ------------------A..........................................................I.G 80

*D. plexippus* ..............I..........................................................L...I.N 80

*D. gilippus* .........................................................................L...I.N 80

*H. erato* .L...L........I............................G.................................I.N 80

*H. melpomene* ..............I............................G.................................I.N 80

*A. vanillae* ..............I............................G.................................I.N 80

*S. mormonia* ..............I............................G.................................I.K 80

*C. tullia* ..............I............................G..........................S........G 80

*B. anynana* ..............V.....N......................G.A....T...................S......TIG 80

*N. ridingsii* .............................................................................I.N 80

*O. chryxus* ..........................V..................................................I.N 80

*L. rubidus* ..............L..............................P...............................I.N 80

*L. helloides* ..............L..............................................................I.N 80

*L. heteronea* ..............F..............................................................I.N 80

*L. nivalis* ..............L..............................................................I.N 80

*S. behrii* ...............................................................................N 80

*A. glandon* ..........................V................G...................................N 80

*P. icarus* --...G........I.................G..............................................N 80

*A. mormo* ..............F............................G.A.................................N 80

*C. philodice* ..............T..............................................................I.N 80

*P. rapae* ..............T..............................................................ISN 80

*P. glaucus* ..............T.....M...S..................GS.....V..........................I.N 80

*P. xuthus* ..............T.....M......................GS.....V..........................I.N 80

*M. sexta* .............................................................................I.N 80

*B. mori* ...............................................................M........M....L.N 80

*V. cardui* MSFDQMPLFVWAVGITALLLLLSLPVLAGAITMLLTDRNINTSFFDPAGGGDPISYQHLFWFFG?????????PGFGMIT 160

*E. chalcedona* ......................................................L.........H?EVYILIL......S 160

*N. antiopa* ......................................................L.........????YILIL......S 160

*L. arthemis* ......S..I.S...........................L..............L.........HPEVYILIL......S 160

*L. archippus* ......S..I.S...........................L..............L.........HPEVYILIL......S 160

*D. plexippus* .T.....................................L..............L.........HPEVYILIL......S 160

*D. gilippus* .L.......I.......V.....................L..............L.........HPEVYILIL......S 160

*H. erato* .....L.................................L..............L.........HPEVYILIL......S 160

*H. melpomene* .....L.................................L..............L.........HPEVYILIL......S 160

*A. vanillae* .....L.................................L..............L.........HPEVYILIL......S 160

*S. mormonia* .......................................L..............L.........HPEVYILIL......S 160

*C. tullia* ..Y....................................L..............L.........HPEVYILIL......S 160

*B. anynana* ..YS.........S.........................L..............L........V??????LIL......S 160

*N. ridingsii* .TY....................................L..............L.........HPEVYILIL......S 160

*O. chryxus* .TY....................................L..............L.........HPEVYILIL......S 160

*L. rubidus* L.....S..I.............................L..............L.........HPEVYILIL....I.S 160

*L. helloides* L.....S..I.............................L..............L.........HPEVYILIL....I.S 160

*L. heteronea* L.....S..I.............................L..............L.........HPEVYILIL....I.S 160

*L. nivalis* L.....S..I.............................L..............L.........HPEVYILIL....I.S 160

*S. behrii* L.....S..I.............................L..............L.........HPEVYILIL....I.S 160

*A. glandon* L.....S..I.............................L..............L.........HPEVYILIL....I.S 160

*P. icarus* L.....S..I.............................L..............L.........HPEVYILIL....I.S 160

*A. mormo* ...........S...........................L..............L.........HPEVYILIL....I.S 160

*C. philodice* .......................................L..............L.........HPEVYILIL......S 160

*P. rapae* .......................................L..............L.........HPEVYILIL......S 160

*P. glaucus* .......................................L..............L.........HPEVYILIL......S 160

*P. xuthus* .......................................L...........N..L.........HPEVYILIL......S 160

*M. sexta* .................F.....................L..............L.........HPEVYILIL......S 160

*B. mori* .....L...........F.....................L..............L.........HPEVYILIL......S 160

*V. cardui* HIISQESGKKETFGCLGMIYAMMAIGLLGFIVWAHHMFTVGMDIDTRAYFTSATMIIAVPTGIKIFSWLATLHGTQINYS 240

*E. chalcedona* ..............Y................................................................. 240

*N. antiopa* ................................................................................ 240

*L. arthemis* .M............Y........................................................I........ 240

*L. archippus* .M............Y........................................................I........ 240

*D. plexippus* ..............S........................................................I........ 240

*D. gilippus* ..............S........................................................M........ 240

*H. erato* ................................................................................ 240

*H. melpomene* ................................................................................ 240

*A. vanillae* ................................................................................ 240

*S. mormonia* ................................................................................ 240

*C. tullia* ......................L........................................................N 240

*B. anynana* ..............Y.......L......................................................... 240

*N. ridingsii* ......................L......................................................... 240

*O. chryxus* ......................L......................................................... 240

*L. rubidus* ..............S.......L................I...............................I........ 240

*L. helloides* ..............S.......L..............................................T.I........ 240

*L. heteronea* ..............S.......L................................................I........ 240

*L. nivalis* ..............S.......L................................................I........ 240

*S. behrii* ..............S.......L................................................IY....... 240

*A. glandon* ..............V.......L................................................IY....I.. 240

*P. icarus* ..............A.......L................................................IY....... 240

*A. mormo* ..............S........................I.....................................SLN 240

*C. philodice* ..............S.........................................................Y....... 240

*P. rapae* ..............S.........................................................Y....... 240

*P. glaucus* ...........................................T.................................... 240

*P. xuthus* ...........................................T...........................F........ 240

*M. sexta* .......T...............................I.......................................N 240

*B. mori* ......................L................................................M.......N 240

*V. cardui* PSMLWSLGFIFLFTVGGLTGVILANSSIDITLHDTYYVVAHFHYVLSMGAVFAILGGFIHWYPLFTGLMMNNYLLKIQFI 320

*E. chalcedona* .....................V................................M......................... 320

*N. antiopa* ..........................................................V..................... 320

*L. arthemis* .....................V................................F.............A..P........ 320

*L. archippus* .....................V................................F.............T..P........ 320

*D. plexippus* ..I......V...........V..............................................TL.P........ 320

*D. gilippus* ..I......V...........V..............................................TL.P........ 320

*H. erato* .....................V....................................V...S......L.P........ 320

*H. melpomene* .....................V....................................V...S.....LL.P........ 320

*A. vanillae* .....................V....................................V..........L.P........ 320

*S. mormonia* ....................................................................TLSP........ 320

*C. tullia* ..............................A...........................V.........IL.P........ 320

*B. anynana* ..............................A.......................I.............TL.PF....... 320

*N. ridingsii* ...........................L..A.......................F...V.........VL.P........ 320

*O. chryxus* ...........................L..A.......................F...V..........L.P........ 320

*L. rubidus* ......................................................F....F........FL.P........ 320

*L. helloides* ......................................................F....F........YL.P........ 320

*L. heteronea* .....................V................................F....F...S....YL.P........ 320

*L. nivalis* ...M..................................................F....F........YL.P........ 320

*S. behrii* ......................................................F.............L..P.Y...... 320

*A. glandon* ......................................................F.............CL.......... 320

*P. icarus* ......................................................F.............YL.......... 320

*A. mormo* .P.......V............................................FA............SL.SFY...... 320

*C. philodice* .........V....................I......................................L.PFY...... 320

*P. rapae* .........V....................I.....................................SL...Y...... 320

*P. glaucus* ..I......V...................V........................M.S...........SL.P.......F 320

*P. xuthus* ..I......V...................V........................M.S...........S..P.......F 320

*M. sexta* ..I......V............................................M..........L..NL.P.......F 320

*B. mori* .NI......V............................................I....N........SL.S.M.....F 320

*V. cardui* SMFIGVN 327

*E. chalcedona* ....... 327

*N. antiopa* ....... 327

*L. arthemis* ...L... 327

*L. archippus* ...L... 327

*D. plexippus* ...L... 327

*D. gilippus* ...L... 327

*H. erato* ....... 327

*H. melpomene* ....... 327

*A. vanillae* ....... 327

*S. mormonia* T...... 327

*C. tullia* ....... 327

*B. anynana* ....... 327

*N. ridingsii* ....... 327

*O. chryxus* ....... 327

*L. rubidus* I...... 327

*L. helloides* I..MA.. 327

*L. heteronea* I...... 327

*L. nivalis* I...... 327

*S. behrii* I...... 327

*A. glandon* I...... 327

*P. icarus* I...... 327

*A. mormo* ....... 327

*C. philodice* T...... 327

*P. rapae* V...... 327

*P. glaucus* T..F... 327

*P. xuthus* T..F... 327

*M. sexta* I..L... 327

*B. mori* T...... 327
