# Supplementary material for: Impact of duplicate gene copies on phylogenetic analysis and divergence time estimates in butterflies
Source: BMC Evol Biol. 2009 May 13;9:99. doi: 10.1186/1471-2148-9-99 (PMC2689175; doi:10.1186/1471-2148-9-99)
Supplement: Additional file 3 — Trees from combined and individual analyses of slow-evolving-copy genes. The maximum likelihood topologies presented were recovered using the same alignment used to generate Figure 1 (maximum parsimony) and Figure 2 (Bayesian). [file 1471-2148-9-99-S3.doc]

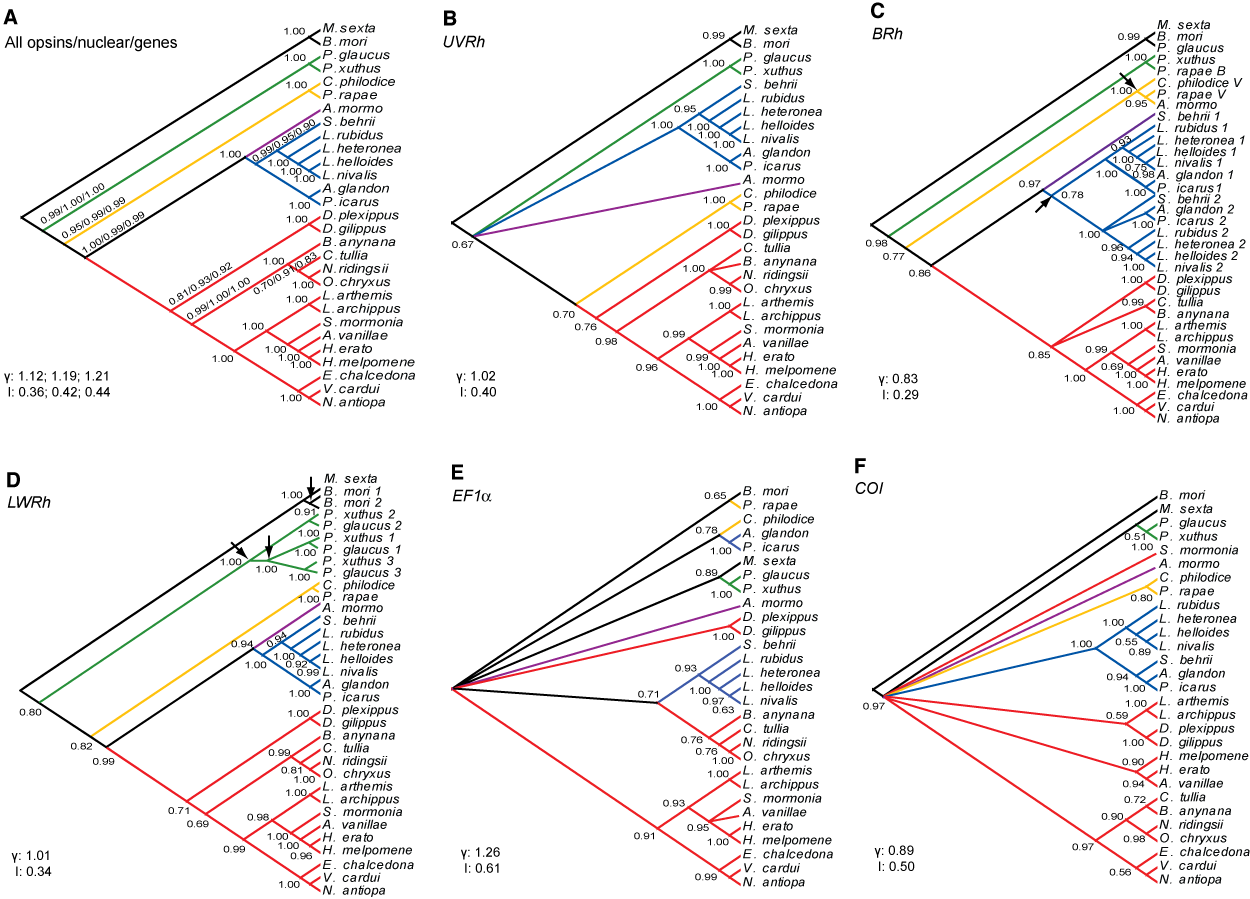


**Additional File 3**

**Maximum likelihood trees from combined and individual slow-evolving-copy genes.**

Topologies obtained from maximum likelihood (ML) analyses of combined and individual gene data. Numbers above and below branches represent clade support as proportion of 500 bootstrap samples. (A) An identical topology was obtained with all three combined data sets using the slower evolving copies of duplicated genes with numbers corresponding to proportion of bootstrap samples obtained from all opsins / all nuclear / all genes datasets, respectively. Only one bootstrap support is shown for clades in which all three data sets result in the same value. Traditional butterfly families are colored as in Figure 2. (B-F) ML topologies obtained for individual genes: (B) *UVRh*, (C) *BRh,* (D) *LWRh,* (E) *EF-1α,* (F) *COI*. Displayed in the lower left corner of each panel are the shape of the gamma parameter (γ) and proportion of invariant sites (I) used in maximum likelihood analyses. Nodes with bootstrap support under 0.50 are showed as polytomies.
