# Supplementary material for: Impact of duplicate gene copies on phylogenetic analysis and divergence time estimates in butterflies
Source: BMC Evol Biol. 2009 May 13;9:99. doi: 10.1186/1471-2148-9-99 (PMC2689175; doi:10.1186/1471-2148-9-99)
Supplement: Additional file 4 — Trees from combined and individual analyses of fast-evolving-copy genes. The maximum parsimony, maximum likelihood and Bayesian analyses of fast-evolving-copy genes recovered identical topologies. [file 1471-2148-9-99-S4.doc]

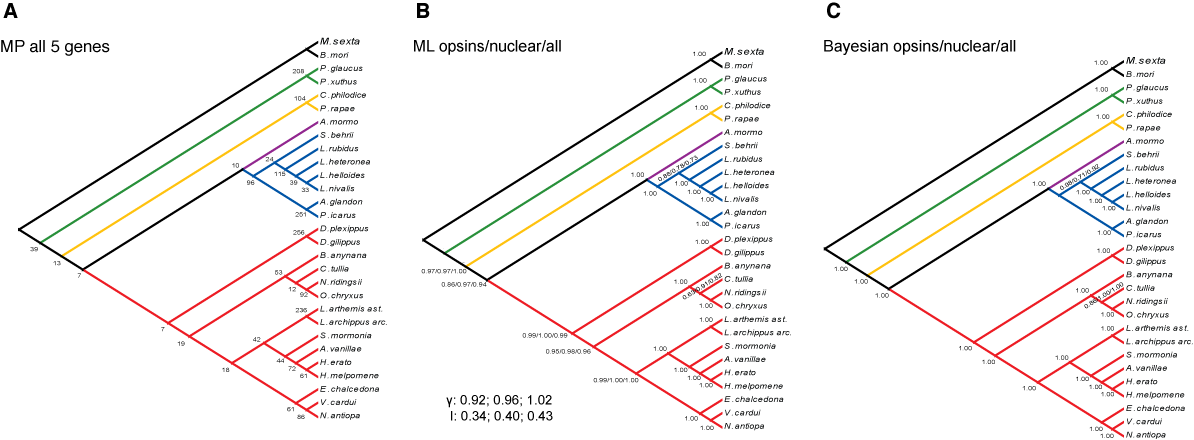


**Additional File 4**

**Trees from combined and individual analyses of fast-evolving-copy genes.**

Identical topologies obtained from maximum parsimony (MP), maximum likelihood (ML) and Bayesian analyses of combined data sets of all opsins, all nuclear and all 5 genes using faster evolving copies of duplicated genes. Numbers above and below branches represent clade support as decay indexes (Bremer support values) in the case of (A) MP, (B) proportion of 500 bootstrap samples in ML and (C) Bayesian posterior probabilities. Only one bootstrap support/posterior probability is shown for clades in which all three data sets result in the same value (B and C).
