# Supplementary material for: Impact of duplicate gene copies on phylogenetic analysis and divergence time estimates in butterflies
Source: BMC Evol Biol. 2009 May 13;9:99. doi: 10.1186/1471-2148-9-99 (PMC2689175; doi:10.1186/1471-2148-9-99)
Supplement: Additional file 5 — Penalized likelihood age estimates. The data provided represent estimates calculated from alignments including either slow- or fast-evolving copies of duplicate genes. [file 1471-2148-9-99-S5.doc]

**Additional File 5**. Age estimates in millions of years of internal nodes of the topology shown in Figure 1.

| **Node** | **Node name** | **Slow** | **Fast** |
| --- | --- | --- | --- |
| 1 | *Papilio* | 65.00 | 62.28 |
| 2 | *Colias + Pieris* | 98.85 | 107.17 |
| 3 | *Agriades + Polyommatus* | 9.98 | 12.53 |
| 4 | *L. helloides + L. nivalis* | 3.96 | 6.72 |
| 5 | *(L. helloides + L. nivalis) L. heteronea* | 8.54 | 12.39 |
| 6 | *((L. helloides + L. nivalis) L. heteronea) L. rubidus* | 17.91 | 23.37 |
| 7 | Lycaeninae + *Satyrium* | 70.34 | 66.75 |
| 8 | (Lycaeninae + *Satyrium)( Agriades + Polyommatus)* | 90.25 | 87.41 |
| 9 | Lycaenidae + *Apodemia* | 151.61 | 138.67 |
| 10 | *Danaus* | 22.33 | 24.45 |
| 11 | *Neominois + Oeneis* | 24.29 | 27.69 |
| 12 | *(N. ridingsii + O. chryxus) C. tullia* | 75.43 | 73.07 |
| 13 | *((N. ridingsii + O. chryxus) C. tullia) Bicyclus* | 94.12 | 87.12 |
| 14 | *Heliconius* | 16.98 | 19.31 |
| 15 | *Heliconius* + *Agraulis* | 35.12 | 36.23 |
| 16 | *((Heliconius) Agraulis) Speyeria* | 61.68 | 60.22 |
| 17 | *Limenitis* | 7.70 | 7.93 |
| 18 | *Limenitis +* node 16 | 88.30 | 79.88 |
| 19 | *Vanessa + Nymphalis* | 34.00 | 34.64 |
| 20 | *(Vanessa + Nymphalis) Euphydryas* | 69.60 | 68.62 |
| 21 | Node 18 + node 20 | 117.02 | 106.22 |
| 22 | Node 21 + node 13 | 141.18 | 125.76 |
| 23 | Node 22 + node 10 | 156.04 | 146.12 |
| 24 | Node 23 + node 9 | 176.26 | 164.34 |
| 25 | Node 24 + node 2 | 197.19 | 182.80 |
| 26 | Node 25 + node 1 | 240.44 | 210.88 |

Age estimates calculated through penalized likelihood in r8s using a smoothing parameter of 3.2 as found by cross validation analyses. Shown are divergence time estimates calculated using all 5 genes, first using the slower evolving copies of duplicated genes (slow) and second using the faster evolving copies (fast).
