# Supplementary material for: Impact of duplicate gene copies on phylogenetic analysis and divergence time estimates in butterflies
Source: BMC Evol Biol. 2009 May 13;9:99. doi: 10.1186/1471-2148-9-99 (PMC2689175; doi:10.1186/1471-2148-9-99)
Supplement: Additional file 6 — Bayesian age estimates. The data provided represent estimates obtained using the slow-evolving-copy gene data set and several combinations of prior values. [file 1471-2148-9-99-S6.doc]

**Additional File 6**. Age estimates in millions of years of internal nodes of the topology shown in Fig. 1. Age estimates calculated through MCMC Bayesian analyses in PAML/Multidivtime. These results were obtained using different combinations of prior values, reported at the top of each column, which respectively represent the age of the ingroup node, the rate of evolution and the variation of the rate of evolution over time (brownmean). Shown are divergence time estimates and 95% confidence intervals calculated using all 5 genes including the slower evolving copies of duplicated genes. An asterisk marks the combination of prior values which estimates are shown in figure 4.

| Node | Node name | 70, 0.04, 0.04 | 70, 0.02, 0.02 | 70, 0.02, 0.002 | 70, 0.02, 0.0002 | 70, 0.002, 0.02 * | 70, 0.002, 0.002 |
| --- | --- | --- | --- | --- | --- | --- | --- |
| 1 | *Papilio* | 58.8 (46.5-64.8) | 58.9 (46.8-64.8) | 60.1 (49.8-64.8) | 61.3 (53.4-64.9) | 59.2 (47.2-64.8) | 60.1 (50.1-64.8) |
| 2 | *Colias + Pieris* | 72.4 (54.7-92.2) | 73.1 (54.7-92.9) | 77.2 (60.0-95.5) | 83.7 (68.4-100.9) | 74.4 (56.4-94.1) | 77.7 (60.4-96.6) |
| 3 | *Agriades + Polyommatus* | 12.5 (7.4-21.2) | 12.3 (7.3-20.8) | 11.7 (7.6-18.2) | 11.5 (8.3-15.7) | 12.4 (7.6-20.5) | 11.9 (7.8-18.2) |
| 4 | *L. helloides + L. nivalis* | 6.3 (3.6-10.0) | 6.3 (3.7-9.8) | 6.6 (4.1-9.9) | 7.0 (4.9-9.8) | 6.5 (3.8-10.00) | 6.7 (4.1-9.9) |
| 5 | *(L. helloides + L. nivalis) L. heteronea* | 12.0 (7.3-18.5) | 11.9 (7.4-18.2) | 11.9 (7.8-17.4) | 11.8 (8.5-16.1) | 12.1 (7.5-18.4) | 12.0 (7.9-17.6) |
| 6 | *((L. helloides + L. nivalis) L. heteronea) L. rubidus* | 20.9 (13.3-31.8) | 20.8 (13.3-31.1) | 20.0 (13.5-28.8) | 18.9 (13.9-25.3) | 20.9 (13.5-31.2) | 20.2 (13.7-29.3) |
| 7 | Lycaeninae + *Satyrium* | 58.6 (43.6-76.3) | 58.9 (43.8-76.6) | 59.9 (46.0-76.0) | 60.9 (49.1-74.4) | 59.9 (45.0-77.4) | 60.5 (46.7-76.6) |
| 8 | (Lycaeninae + *Satyrium)( Agriades + Polyommatus)* | 71.1 (53.9-91.4) | 71.6 (54.0-91.4) | 74.1 (58.1-92.5) | 77.6 (63.7-93.3) | 73.1 (56.3-93.0) | 75.0 (59.2-93.0) |
| 9 | Lycaenidae + *Apodemia* | 104.0 (78.9-132.7) | 105.3 (79.5-132.9) | 112.7 (90.5-137.3) | 122.8 (103.4-144.3) | 108.0 (83.6-135.1) | 113.8 (92.0-138.1) |
| 10 | *Danaus* | 23.0 (14.7-35.0) | 23.0 (14.9-35.3) | 22.5 (17.7-32.2) | 22.3 (16.8-29.1) | 23.1 (15.1-34.7) | 22.6 (15.7-32.4) |
| 11 | *Neominois + Oeneis* | 26.4 (17.4-38.1) | 26.3 (17.3-37.8) | 26.4 (18.5-36.5) | 26.5 (20.0-34.2) | 26.6 (17.9-38.0) | 26.5 (18.5-36.2) |
| 12 | *(N. ridingsii + O. chryxus) C. tullia* | 62.7 (47.7-79.8) | 63.1 (47.7-80.7) | 65.8 (52.2-81.9) | 69.7 (57.2-84.3) | 64.3 (49.7-81.7) | 66.3 (52.2-82.3) |
| 13 | *((N. ridingsii + O. chryxus) C. tullia) Bicyclus* | 72.4 (56.3-91.6) | 73.0 (56.3-92.3) | 76.5 (61.9-93.8) | 81.5 (68.1-97.3) | 74.5 (58.6-93.3) | 77.2 (62.5-94.5) |
| 14 | *Heliconius* | 17.9 (12.1-25.4) | 17.9 (12.1-25.1) | 18.0 (12.7-24.4) | 18.7 (14.2-24.0) | 18.1 (12.3-25.3) | 18.1 (13.0-24.7) |
| 15 | *Heliconius* + *Agraulis* | 32.3 (23.3-42.9) | 32.4 (23.4-42.7) | 33.0 (24.8-42.5) | 34.7 (27.5-42.9) | 32.8 (24.0-43.4) | 33.2 (25.1-42.9) |
| 16 | *((Heliconius) Agraulis) Speyeria* | 51.8 (40.1-65.8) | 52.0 (40.4-65.8) | 53.6 (42.7-66.6) | 56.4 (46.6-67.6) | 52.9 (41.2-66.8) | 53.9 (42.9-66.7) |
| 17 | *Limenitis* | 8.0 (4.6-14.4) | 7.9 (4.6-14.0) | 7.6 (4.9-11.8) | 7.4 (5.2-10.1) | 7.9 (4.7-13.6) | 7.6 (4.9-11.6) |
| 18 | *Limenitis +* node 16 | 66.6 (52.9-82.8) | 67.0 (53.3-82.9) | 69.8 (57.1-85.1) | 74.0 (62.5-87.5) | 68.3 (55.0-84.8) | 70.2 (57.4-85.4) |
| 19 | *Vanessa + Nymphalis* | 37.1 (34.1-45.1) | 37.0 (34.1-44.9) | 36.7 (34.1-43.6) | 36.2 (34.1-41.6) | 37.1 (34.1-45.1) | 36.7 (34.1-43.7) |
| 20 | *(Vanessa + Nymphalis) Euphydryas* | 60.8 (50.7-73.9) | 61.1 (50.7-73.8) | 63.1 (53.8-75.4) | 65.7 (57.3-76.6) | 62.0 (52.2-75.3) | 63.3 (54.0-75.3) |
| 21 | Node 18 + node 20 | 84.6 (66.5-105.1) | 85.5 (67.0-105.7) | 90.5 (74.6-109.1) | 97.4 (83.8-113.8) | 87.4 (70.0-107.3) | 91.1 (75.5-109.4) |
| 22 | Node 21 + node 13 | 96.6 (74.5-121.1) | 97.6 (74.9-122.4) | 104.2 (85.2-125.7) | 113.2 (97.0-132.2) | 100.2 (78.8-123.8) | 105.1 (86.0-127.0) |
| 23 | Node 22 + node 10 | 106.5 (80.9-134.9) | 107.8 (81.4-135.8) | 115.7 (93.4-140.2) | 126.5 (107.8-147.7) | 110.8 (86.1-138.2) | 116.8 (94.9-141.4) |
| 24 | Node 23 + node 9 | 116.7 (87.2-149.6) | 118.3 (88.0-150.3) | 127.8 (102.4-155.7) | 140.7 (119.7-164.4) | 121.8 (93.3-152.7) | 129.1 (103.9-157.2) |
| 25 | Node 24 + node 2 | 127.9 (93.6-165.7) | 129.7 (94.5-166.8) | 140.9 (111.1-173.2) | 155.8 (131.9-182.6) | 133.9 (101.1-170.0) | 142.6 (113.4-174.9) |
| 26 | Node 25 + node 1 | 145.9 (103.9-191.7) | 148.2 (104.6-193.9) | 161.7 (125.4-200.4) | 178.3 (151.0-208.8) | 153.7 (113.1-197.4) | 163.8 (128.4-203.0) |

Continuation Additional File 6.

| Node | Node name | 70, 0.002, 0.0002 | 70, 0.0002, 0.02 | 70, 0.0002, 0.002 | 70, 0.0002, 0.0002 | 100, 0.04,0.04 | 100, 0.02, 0.02 |
| --- | --- | --- | --- | --- | --- | --- | --- |
| 1 | *Papilio* | 61.5 (53.8-64.9) | 59.9 (48.4-64.9) | 60.7 (51.3-64.9) | 61.7 (54.3-64.9) | 59.0 (47.2-64.8) | 59.1 (47.1-64.8) |
| 2 | *Colias + Pieris* | 84.3 (68.8-102.1) | 80.2 (60.3-102.6) | 82.2 (64.0-102.7) | 87.1 (71.3-105.1) | 73.0 (54.4-93.3) | 73.8 (55.4-93.9) |
| 3 | *Agriades + Polyommatus* | 11.5 (8.3-15.6) | 13.1 (8.1-21.9) | 12.4 (8.1-19.3) | 11.9 (8.6-16.1) | 12.6 (7.3-21.7) | 12.5 (7.4-21.0) |
| 4 | *L. helloides + L. nivalis* | 7.1 (4.9-9.9) | 7.0 (4.2-10.8) | 7.1 (4.5-10.6) | 7.4 (5.1-10.2) | 6.4 (3.7- 10.0) | 6.4 (3.7-10.1) |
| 5 | *(L. helloides + L. nivalis) L. heteronea* | 11.9 (8.5-16.1) | 13.0 (8.2-19.8) | 12.7 (8.3-18.8) | 12.3 (8.8-16.9) | 12.1 (7.4-18.6) | 12.1 (7.4-18.6) |
| 6 | *((L. helloides + L. nivalis) L. heteronea) L. rubidus* | 19.0 (13.9-25.4) | 22.3 (14.4-34.0) | 21.2 (14.3-31.5) | 19.7 (14.5-26.3) | 21.1 (13.5-31.6) | 21.0 (13.4-31.5) |
| 7 | Lycaeninae + *Satyrium* | 61.2 (49.3-75.0) | 65.0 (48.7-86.1) | 64.3 (49.1-82.9) | 63.5 (50.9-78.3) | 59.3 (43.9-77.0) | 59.5 (44.6-77.1) |
| 8 | (Lycaeninae + *Satyrium)( Agriades + Polyommatus)* | 78.0 (63.7-94.1) | 79.9 (61.3-102.7) | 80.0 (62.7-100.3) | 81.0 (66.2-98.3) | 71.9 (54.4-92.2) | 72.5 (55.0-92.0) |
| 9 | Lycaenidae + *Apodemia* | 123.7 (104.5-146.0) | 120.6 (95.9-149.2) | 123.1 (100.4-148.8) | 128.8 (108.9-151.8) | 105.2 (79.0-134.1) | 106.8 (80.9-133.7) |
| 10 | *Danaus* | 22.4 (17.0-29.1) | 23.7 (16.0-35.0) | 23.4 (16.5-33.2) | 23.0 (17.4-30.2) | 23.3 (14.9-36.1) | 23.0 (15.0-34.7) |
| 11 | *Neominois + Oeneis* | 26.6 (20.1-34.8) | 28.6 (19.4-41.2) | 28.1 (19.8-39.3) | 27.5 (20.7-36.0) | 26.7 (17.5-38.5) | 26.5 (17.6-38.0) |
| 12 | *(N. ridingsii + O. chryxus) C. tullia* | 70.1 (57.8-84.6) | 70.1 (54.2-89.7) | 70.7 (55.9-89.1) | 72.6 (59.4-87.9) | 63.3 (48.1-81.0) | 63.9 (48.7-81.0) |
| 13 | *((N. ridingsii + O. chryxus) C. tullia) Bicyclus* | 82.0 (68.5-97.6) | 81.5 (64.4-102.5) | 82.4 (66.8-102.0) | 85.0 (71.0-101.5) | 73.1 (56.7-92.5) | 73.9 (57.7-92.8) |
| 14 | *Heliconius* | 18.7 (14.2-24.2) | 19.2 (13.4-27.3) | 19.1 (13.8-26.0) | 19.3 (14.7-25.1) | 18.1 (12.1-25.9) | 18.0 (12.2-25.4) |
| 15 | *Heliconius* + *Agraulis* | 34.8 (27.6-43.2) | 35.1 (25.9-47.2) | 35.0 (26.7-45.5) | 36.0 (28.4-44.9) | 32.6 (23.7-43.4) | 32.6 (23.8-43.3) |
| 16 | *((Heliconius) Agraulis) Speyeria* | 56.7 (46.9-68.3) | 56.9 (44.5-72.3) | 57.2 (45.6-71.7) | 58.5 (48.1-70.5) | 52.2 (40.4-66.3) | 52.5 (40.9-66.5) |
| 17 | *Limenitis* | 7.4 (5.3-10.2) | 8.2 (5.1-13.4) | 7.9 (5.1-12.1) | 7.7 (5.4-10.6) | 8.1 (4.7-14.7) | 7.9 (4.7-13.9) |
| 18 | *Limenitis +* node 16 | 74.4 (62.5-88.3) | 74.0 (59.4-92.1) | 74.7 (61.1-92.0) | 76.9 (64.6-91.4) | 67.2 (53.3-83.8) | 67.7 (53.9-84.2) |
| 19 | *Vanessa + Nymphalis* | 36.2 (34.1-41.8) | 38.0 (34.1-47.9) | 37.4 (34.1-46.0) | 36.7 (34.1-43.1) | 37.1 (34.1-45.1) | 37.0 (34.1-44.9) |
| 20 | *(Vanessa + Nymphalis) Euphydryas* | 66.0 (57.4-76.8) | 66.2 (55.2-81.4) | 66.5 (56.4-80.9) | 67.8 (58.7-79.3) | 61.2 (50.8-74.5) | 61.6 (51.4-74.7) |
| 21 | Node 18 + node 20 | 98.0 (84.0-114.5) | 95.8 (77.8-117.2) | 97.4 (81.4-117.9) | 101.3 (86.8-118.2) | 85.4 (67.0-106.8) | 86.5 (68.3-106.6) |
| 22 | Node 21 + node 13 | 114.0 (97.5-132.9) | 110.9 (89.2-136.1) | 113.2 (93.7-136.4) | 118.2 (101.0-138.0) | 97.6 (74.6-123.3) | 99.0 (76.5-123.3) |
| 23 | Node 22 + node 10 | 127.4 (108.6-149.1) | 123.7 (98.8-151.5) | 126.5 (104.3-152.3) | 132.5 (113.1-154.9) | 107.7 (81.0-137.5) | 109.4 (83.2-137.0) |
| 24 | Node 23 + node 9 | 141.8 (120.4-166.2) | 137.3 (109.5-169.0) | 140.7 (115.6-169.3) | 147.9 (126.1-173.2) | 118.1 (87.5-152.1) | 120.2 (89.9-151.3) |
| 25 | Node 24 + node 2 | 157.1 (132.9-184.6) | 153.6 (121.4-190.0) | 157.2 (128.4-190.3) | 164.9 (139.9-193.8) | 129.4 (94.1-168.7) | 132.0 (96.7-168.7) |
| 26 | Node 25 + node 1 | 179.8 (152.0-211.2) | 180.6 (142.9-226.1) | 183.5 (149.8-223.8) | 189.5 (161.1-222.5) | 147.9 (104.1-195.7) | 151.0 (108.2-196.3) |

Continuation Additional File 6

| Node | Node name | 100, 0. 02, 0.002 | 100, 0. 02, 0.0002 | 100, 0.002, 0.02 * | 100, 0.002, 0.002 | 100, 0.002, 0.0002 | 100, 0.0002, 0.02 |
| --- | --- | --- | --- | --- | --- | --- | --- |
| 1 | *Papilio* | 60.2 (49.8-64.8) | 61.4 (53.6-64.9) | 59.3 (47.8-64.8) | 60.2 (59.0-64.8) | 61.5 (53.8-64.9) | 59.9 (48.5-64.8) |
| 2 | *Colias + Pieris* | 77.5 (60.3-96.5) | 84.3 (69.1-101.0) | 74.9 (56.6-94.7) | 78.0 (61.1-96.5) | 84.7 (69.3-102.2) | 81.1 (60.4-104.0) |
| 3 | *Agriades + Polyommatus* | 11.9 (7.7-18.5) | 11.6 (8.3-15.5) | 12.6 (7.6-21.8) | 11.9 (7.7-18.6) | 11.6 (8.4-15.8) | 13.3 (8.2-22.6) |
| 4 | *L. helloides + L. nivalis* | 6.6 (4.2-9.9) | 7.1 (4.9-9.9) | 6.5 (3.8-10.3) | 6.7 (4.2-10.1) | 7.1 (4.9-9.9) | 7.2 (4.3-11.2) |
| 5 | *(L. helloides + L. nivalis) L. heteronea* | 12.0 (7.8-17.7) | 11.9 (8.6-16.2) | 12.2 (7.6-18.8) | 12.1 (7.9-17.8) | 12.0 (8.6-16.3) | 13.2 (8.3-20.6) |
| 6 | *((L. helloides + L. nivalis) L. heteronea) L. rubidus* | 20.3 (13.7-29.7) | 19.0 (14.0-25.2) | 21.2 (13.6-31.8) | 20.4 (13.7-29.9) | 19.1 (14.0-25.3) | 22.7 (14.5-35.5) |
| 7 | Lycaeninae + *Satyrium* | 60.4 (46.8-77.3) | 61.3 (49.5-74.9) | 60.5 (45.3-79.0) | 60.7 (46.6-77.6) | 61.6 (49.8-75.4) | 65.8 (49.2-87.6) |
| 8 | (Lycaeninae + *Satyrium)( Agriades + Polyommatus)* | 74.7 (58.8-93.4) | 78.2 (64.3-94.0) | 73.7 (56.4-94.2) | 75.2 (59.5-94.4) | 78.4 (64.3-94.9) | 80.8 (62.2-104.6) |
| 9 | Lycaenidae + *Apodemia* | 113.4 (91.1-138.4) | 123.8 (104.7-145.2) | 108.7 (83.4-136.2) | 114.5 (92.2-139.7) | 124.2 (105.2-146.1) | 121.6 (96.3-151.1) |
| 10 | *Danaus* | 22.6 (15.7-32.4) | 22.4 (16.9-29.1) | 23.2 (15.3-35.6) | 22.7 (15.9-32.6) | 22.5 (17.1-29.3) | 24.0 (16.0-36.5) |
| 11 | *Neominois + Oeneis* | 26.6 (18.6-36.8) | 26.6 (20.1-34.4) | 26.8 (17.7-38.2) | 26.7 (18.9-36.6) | 26.7 (20.0-34.6) | 28.8 (19.5-41.7) |
| 12 | *(N. ridingsii + O. chryxus) C. tullia* | 66.2 (52.3-82.6) | 70.1 (57.5-84.3) | 64.7 (49.7-82.0) | 66.6 (52.5-82.5) | 70.4 (58.0-84.6) | 70.7 (54.6-90.7) |
| 13 | *((N. ridingsii + O. chryxus) C. tullia) Bicyclus* | 77.0 (61.8-94.7) | 82.0 (68.8-97.2) | 74.9 (58.4-93.6) | 77.6 (62.4-94.8) | 82.3 (68.8-97.9) | 82.2 (64.7-103.4) |
| 14 | *Heliconius* | 18.1 (13.0-24.6) | 18.8 (14.3-24.3) | 18.2 (12.4-25.6) | 18.2 (12.9-24.8) | 18.8 (14.4-24.3) | 19.5 (13.5-28.0) |
| 15 | *Heliconius* + *Agraulis* | 33.2 (25.1-43.0) | 34.8 (27.6-43.2) | 33.0 (24.1-43.6) | 33.3 (25.0-43.1) | 34.9 (27.9-43.2) | 35.5 (26.1-47.9) |
| 16 | *((Heliconius) Agraulis) Speyeria* | 53.9 (43.0-67.3) | 56.7 (46.9-68.2) | 53.1 (41.4-67.1) | 54.2 (43.2-67.3) | 56.8 (47.0-68.3) | 57.5 (44.9-74.2) |
| 17 | *Limenitis* | 7.6 (4.9-11.7) | 7.4 (5.3-10.1) | 7.9 (4.7-13.5) | 7.6 (5.0-11.8) | 7.5 (5.3-10.2) | 8.3 (5.1-14.1) |
| 18 | *Limenitis +* node 16 | 70.2 (57.5-85.9) | 74.4 (62.8-88.1) | 68.6 (54.8-85.0) | 70.7 (57.9-86.0) | 74.6 (62.8-88.2) | 74.7 (59.8-94.0) |
| 19 | *Vanessa + Nymphalis* | 36.7 (34.1-43.8) | 36.2 (34.1-41.6) | 37.2 (34.1-45.5) | 36.8 (34.1-43.9) | 36.3 (34.1-41.7) | 38.3 (34.1-44.5) |
| 20 | *(Vanessa + Nymphalis) Euphydryas* | 63.3 (53.9-75.9) | 66.0 (57.4-76.5) | 62.3 (52.0-75.4) | 63.6 (54.3-75.9) | 66.2 (57.6-76.9) | 66.7 (55.4-83.4) |
| 21 | Node 18 + node 20 | 91.0 (74.9-109.7) | 98.0 (84.1-113.7) | 87.8 (69.8-108.1) | 91.6 (75.5-110.1) | 98.3 (84.5-114.3) | 96.6 (78.3-118.9) |
| 22 | Node 21 + node 13 | 104.9 (85.3-127.1) | 114.0 (97.9-132.5) | 100.7 (78.5-124.8) | 105.7 (86.1-127.8) | 114.4 (97.9-133.2) | 111.8 (89.8-137.7) |
| 23 | Node 22 + node 10 | 116.5 (93.7-141.5) | 127.4 (108.9-148.3) | 111.4 (85.6-138.5) | 117.6 (95.0-143.0) | 127.8 (109.1-149.1) | 124.7 (99.6-153.6) |
| 24 | Node 23 + node 9 | 128.7 (102.7-156.6) | 141.8 (120.9-165.6) | 122.5 (92.9-153.6) | 130.0 (103.7-158.4) | 142.3 (121.4-166.2) | 138.5 (109.8-170.5) |
| 25 | Node 24 + node 2 | 141.9 (111.7-174.3) | 157.0 (133.2-184.2) | 134.7 (100.6-170.1) | 143.5 (112.9-176.1) | 157.8 (134.0-185.4) | 155.1 (122.5-192.2) |
| 26 | Node 25 + node 1 | 162.9 (126.3-201.6) | 179.7 (152.2-210.1) | 154.7 (112.8-197.9) | 165.1 (128.9-204.3) | 180.6 (153.4-211.6) | 182.3 (143.9-229.0) |

Continuation Additional file 6

| Node | Node name | 100, 0. 0002, 0.002 | 100, 0. 0002, 0.0002 |
| --- | --- | --- | --- |
| 1 | *Papilio* | 60.7 (51.2-64.9) | 61.8 (54.5-64.9) |
| 2 | *Colias + Pieris* | 83.1 (64.8-103.8) | 87.3 (71.3-105.8) |
| 3 | *Agriades + Polyommatus* | 12.6 (8.2-19.9) | 12.0 (8.7-16.4) |
| 4 | *L. helloides + L. nivalis* | 7.2 (4.5-10.7) | 7.4 (5.1-10.4) |
| 5 | *(L. helloides + L. nivalis) L. heteronea* | 12.9 (8.4-19.2) | 12.4 (8.8-16.9) |
| 6 | *((L. helloides + L. nivalis) L. heteronea) L. rubidus* | 21.6 (14.5-32.2) | 19.7 (14.4-26.5) |
| 7 | Lycaeninae + *Satyrium* | 65.0 (49.8-84.9) | 63.7 (51.4-78.6) |
| 8 | (Lycaeninae + *Satyrium)( Agriades + Polyommatus)* | 80.8 (63.5-102.8) | 81.3 (66.6-98.4) |
| 9 | Lycaenidae + *Apodemia* | 124.2 (101.5-151.9) | 129.1 (108.7-152.1) |
| 10 | *Danaus* | 23.5 (16.3-33.3) | 23.1 (17.5-30.4) |
| 11 | *Neominois + Oeneis* | 28.5 (19.9-39.9) | 27.6 (20.9-36.0) |
| 12 | *(N. ridingsii + O. chryxus) C. tullia* | 71.3 (56.4-90.2) | 72.8 (59.9-87.5) |
| 13 | *((N. ridingsii + O. chryxus) C. tullia) Bicyclus* | 83.2 (67.1-103.5) | 85.1 (71.1-101.5) |
| 14 | *Heliconius* | 19.3 (13.8-26.5) | 19.4 (14.8-25.0) |
| 15 | *Heliconius* + *Agraulis* | 35.4 (26.8-46.2) | 36.1 (28.6-44.8) |
| 16 | *((Heliconius) Agraulis) Speyeria* | 57.6 (45.8-72.7) | 58.7 (48.1-70.9) |
| 17 | *Limenitis* | 7.9 (5.2-12.3) | 7.7 (5.5-10.4) |
| 18 | *Limenitis +* node 16 | 75.3 (61.4-93.0) | 77.1 (64.6-91.6) |
| 19 | *Vanessa + Nymphalis* | 37.6 (34.1-46.7) | 36.7 (34.1-43.0) |
| 20 | *(Vanessa + Nymphalis) Euphydryas* | 66.9 (56.5-81.2) | 67.9 (58.8-79.6) |
| 21 | Node 18 + node 20 | 98.2 (81.4-119.4) | 101.6 (87.1-118.7) |
| 22 | Node 21 + node 13 | 114.1 (94.3-138.7) | 118.5 (101.6-138.3) |
| 23 | Node 22 + node 10 | 127.6 (105.1-154.5) | 132.9 (113.1-155.0) |
| 24 | Node 23 + node 9 | 142.1 (116.2-172.8) | 148.3 (126.0-173.7) |
| 25 | Node 24 + node 2 | 158.9 (129.8-194.2) | 165.4 (139.9-194.6) |
| 26 | Node 25 + node 1 | 185.7 (150.8-228.1) | 190.2 (161.3-223.2) |
