# Supplementary material for: Impact of duplicate gene copies on phylogenetic analysis and divergence time estimates in butterflies
Source: BMC Evol Biol. 2009 May 13;9:99. doi: 10.1186/1471-2148-9-99 (PMC2689175; doi:10.1186/1471-2148-9-99)
Supplement: Additional file 7 — Bayesian age estimates. The data in the table represent estimates obtained using the fast-evolving-copy gene data set and several combinations of prior values. [file 1471-2148-9-99-S7.doc]

**Additional File 7**. Age estimates in millions of years of internal nodes of the topology shown in Fig. 1. Age estimates calculated through MCMC Bayesian analyses in PAML/Multidivtime. These results were obtained using different combinations of prior values, reported at the top of each column, which respectively represent the age of the ingroup node, the rate of evolution and the variation of the rate of evolution over time (brownmean). Shown are divergence time estimates and 95% confidence intervals calculated using all 5 genes including the faster evolving copies of duplicated genes. An asterisk marks the combination of prior values which estimates are shown in additional figure 4.

| Node | Node name | 70, 0.04, 0.04 | 70, 0.02, 0.02 | 70, 0.02, 0.002 | 70, 0.02, 0.0002 | 70, 0.002, 0.02 * | 70, 0.002, 0.002 | 70, 0.002, 0.0002 |
| --- | --- | --- | --- | --- | --- | --- | --- | --- |
| 1 | *Papilio* | 57.9 (45.3-64.7) | 57.9 (45.3-64.7) | 58.4 (46.6-64.8) | 59.8 (49.9-64.8) | 58.0 (45.4-64.8) | 58.4 (46.8-64.8) | 59.9 (49.9-64.8) |
| 2 | *Colias + Pieris* | 90.7 (69.4-113.5) | 91.0 (70.1-113.8) | 92.8 (73.1-114.9) | 98.4 (79.8-119.1) | 91.7 (70.6-115.0) | 93.3 (73.2-115.7) | 98.7 (80.1-120.1) |
| 3 | *Agriades + Polyommatus* | 11.7 (7.6-17.5) | 11.7 (7.7-17.3) | 11.7 (7.8-17.0) | 12.1 (8.7-16.5) | 11.7 (7.7-17.4) | 11.8 (8.0-17.3) | 12.2 (8.7-16.6) |
| 4 | *L. helloides + L. nivalis* | 8.5 (5.2-12.6) | 8.5 (5.4-12.7) | 8.5 (5.5-12.5) | 8.9 (6.2-12.3) | 8.5 (5.4-12.8) | 8.7 (5.6-12.7) | 8.9 (6.1-12.4) |
| 5 | *(L. helloides + L. nivalis) L. heteronea* | 14.4 (9.3-21.0) | 14.4 (9.5-21.1) | 14.4 (9.6-20.6) | 14.5 (10.3-19.8) | 14.5 (9.5-21.3) | 14.6 (9.8-20.9) | 14.6 (10.4-19.9) |
| 6 | *((L. helloides + L. nivalis) L. heteronea) L. rubidus* | 24.6 (16.6-35.5) | 24.7 (16.9-34.9) | 24.4 (17.0-34.3) | 23.8 (17.4-31.8) | 24.8 (16.9-35.3) | 24.6 (17.0-34.8) | 23.9 (17.5-31.9) |
| 7 | Lycaeninae + *Satyrium* | 61.6 (46.3-79.5) | 61.8 (46.7-79.3) | 62.2 (47.5-79.2) | 63.9 (51.0-79.3) | 62.3 (46.8-80.2) | 62.8 (48.0-80.4) | 64.2 (50.9-79.3) |
| 8 | (Lycaeninae + *Satyrium)( Agriades + Polyommatus)* | 79.1 (60.4-100.0) | 79.3 (61.0-99.7) | 80.4 (62.7-100.4) | 84.3 (68.2-102.7) | 80.0 (61.0-100.7) | 81.1 (63.3-101.7) | 84.6 (68.2-103.0) |
| 9 | Lycaenidae + *Apodemia* | 118.4 (91.5-146.5) | 118.7 (92.5-146.9) | 121.5 (97.6-148.4) | 129.6 (108.0-154.5) | 120.0 (93.6-148.1) | 122.5 (97.9-150.0) | 130.2 (107.9-155.6) |
| 10 | *Danaus* | 25.4 (17.7-35.6) | 25.4 (17.7-35.7) | 25.4 (18.0-35.0) | 25.6 (19.1-33.7) | 25.5 (17.6-35.8) | 25.5 (18.1-35.4) | 25.6 (19.2-33.8) |
| 11 | *Neominois + Oeneis* | 27.8 (19.4-38.2) | 28.0 (19.4-38.4) | 28.0 (20.2-38.0) | 28.3 (21.0-37.4) | 28.0 (19.7-38.6) | 28.2 (20.2-38.5) | 28.4 (20.9-37.2) |
| 12 | *(N. ridingsii + O. chryxus) C. tullia* | 69.2 (53.7-86.9) | 69.5 (53.7-86.9) | 70.5 (55.1-87.9) | 73.8 (59.6-90.6) | 70.0 (54.1-88.2) | 71.0 (56.0-88.9) | 74.1 (59.8-91.1) |
| 13 | *((N. ridingsii + O. chryxus) C. tullia) Bicyclus* | 80.6 (63.4-100.1) | 81.0 (63.8-100.5) | 82.3 (65.5-101.7) | 86.4 (71.0-104.7) | 81.6 (64.4-101.6) | 82.9 (66.4-102.3) | 86.7 (71.1-105.1) |
| 14 | *Heliconius* | 18.9 (13.4-25.8) | 19.0 (13.6-25.9) | 19.1 (13.8-25.7) | 19.9 (15.1-25.8) | 19.1 (13.6-25.9) | 19.3 (14.1-26.1) | 20.0 (15.0-26.0) |
| 15 | *Heliconius* + *Agraulis* | 34.8 (25.8-45.4) | 34.9 (26.2-45.2) | 35.2 (26.6-45.6) | 37.0 (29.1-46.4) | 35.2 (26.2-45.8) | 35.5 (27.1-46.1) | 37.1 (29.2-46.8) |
| 16 | *((Heliconius) Agraulis) Speyeria* | 56.8 (44.3-71.5) | 57.1 (45.0-71.7) | 57.7 (45.6-72.0) | 60.5 (49.3-73.8) | 57.5 (44.8-72.1) | 58.2 (46.2-72.7) | 60.6 (49.2-74.2) |
| 17 | *Limenitis* | 8.0 (5.2-12.2) | 8.0 (5.3-12.1) | 8.0 (5.3-11.8) | 8.0 (5.7-11.1) | 8.1 (5.2-12.1) | 8.1 (5.4-11.8) | 8.0 (5.7-11.2) |
| 18 | *Limenitis +* node 16 | 74.5 (59.5-92.2) | 74.8 (59.9-92.4) | 75.8 (61.3-92.9) | 79.7 (66.1-95.9) | 75.4 (60.1-93.1) | 76.5 (62.0-94.0) | 79.9 (66.2-96.5) |
| 19 | *Vanessa + Nymphalis* | 37.3 (34.1-45.4) | 37.4 (34.1-45.4) | 37.3 (34.1-45.4) | 37.2 (34.1-44.4) | 37.4 (34.1-45.5) | 37.4 (34.1-45.6) | 37.2 (34.1-44.8) |
| 20 | *(Vanessa + Nymphalis) Euphydryas* | 66.0 (55.1-80.4) | 66.1 (55.4-80.3) | 66.9 (56.4-80.9) | 69.3 (59.2-82.6) | 66.5 (55.7-80.6) | 67.3 (56.6-81.2) | 69.5 (59.5-82.8) |
| 21 | Node 18 + node 20 | 96.3 (77.2-117.9) | 96.7 (78.0-117.8) | 98.5 (80.7-119.0) | 104.1 (88.1-123.8) | 97.5 (78.3-119.1) | 99.2 (81.5-120.6) | 104.4 (87.8-124.3) |
| 22 | Node 21 + node 13 | 111.4 (88.1-136.8) | 111.9 (89.1-136.7) | 114.3 (92.9-138.4) | 121.3 (102.2-143.9) | 113.0 (89.6-138.1) | 115.1 (93.7-139.3) | 121.8 (102.3-144.9) |
| 23 | Node 22 + node 10 | 125.6 (98.2-154.5) | 126.1 (98.9-154.7) | 129.1 (104.4-156.7) | 137.6 (115.6-162.9) | 127.5 (100.1-156.7) | 130.2 (105.0-158.2) | 138.1 (115.2-164.1) |
| 24 | Node 23 + node 9 | 138.2 (106.8-170.4) | 138.7 (107.9-171.1) | 142.2 (114.2-172.9) | 152.4 (127.6-180.9) | 140.3 (109.1-173.2) | 143.5 (115.1-175.0) | 153.1 (127.7-181.8) |
| 25 | Node 24 + node 2 | 150.9 (115.3-187.4) | 151.4 (117.0-187.8) | 155.5 (123.8-189.9) | 166.9 (139.3-198.6) | 153.4 (118.5-190.0) | 157.0 (125.0-193.1) | 167.7 (139.7-199.4) |
| 26 | Node 25 + node 1 | 170.5 (128.8-213.0) | 171.0 (130.9-213.4) | 175.9 (139.2-216.7) | 188.6 (157.0-224.2) | 173.6 (132.5-216.2) | 177.7 (140.5-219.7) | 189.6 (158.0-225.5) |

Continuation Additional File 7.

| Node | Node name | 70, 0.0002, 0.02 | 70, 0.0002, 0.002 | 70, 0.0002, 0.0002 | 100, 0.04, 0.04 | 100, 0.02, 0.02 | 100, 0. 02, 0.002 |
| --- | --- | --- | --- | --- | --- | --- | --- |
| 1 | *Papilio* | 59.2 (47.9-64.8) | 59.6 (48.7-64.8) | 60.6 (51.2-64.9) | 58.0 (45.7-64.7) | 58.1 (45.8-64.7) | 58.6 (46.9-64.8) |
| 2 | *Colias + Pieris* | 98.1 (76.5-122.6) | 99.1 (78.0-123.0) | 102.9 (83.6-125.3) | 91.2 (69.9-114.3) | 91.3 (70.5-113.8) | 93.5 (73.7-115.7) |
| 3 | *Agriades + Polyommatus* | 12.5 (8.3-18.4) | 12.5 (8.5-17.8) | 12.7 (9.1-17.3) | 11.7 (7.7-17.5) | 11.7 (7.7-17.6) | 11.7 (7.9-17.0) |
| 4 | *L. helloides + L. nivalis* | 9.2 (5.9-13.4) | 9.2 (6.0-13.4) | 9.3 (6.4-13.2) | 8.5 (5.3-12.7) | 8.5 (5.3-12.6) | 8.6 (5.6-12.6) |
| 5 | *(L. helloides + L. nivalis) L. heteronea* | 15.5 (10.3-22.4) | 15.5 (10.5-22.0) | 15.2 (10.8-20.9) | 14.5 (9.5-21.2) | 14.5 (9.4-21.0) | 14.5 (9.8-20.7) |
| 6 | *((L. helloides + L. nivalis) L. heteronea) L. rubidus* | 26.3 (18.1-37.7) | 26.1 (18.2-36.5) | 25.0 (18.2-33.7) | 24.8 (16.9-35.5) | 24.7 (16.9-35.4) | 24.5 (17.2-34.1) |
| 7 | Lycaeninae + *Satyrium* | 66.7 (50.9-86.0) | 66.9 (51.4-85.4) | 67.1 (53.1-83.5) | 62.1 (46.7-80.3) | 62.1 (46.6-79.7) | 62.7 (48.2-79.6) |
| 8 | (Lycaeninae + *Satyrium)( Agriades + Polyommatus)* | 86.0 (67.0-108.2) | 86.7 (67.8-108.0) | 88.5 (71.5-108.3) | 79.6 (60.8-100.6) | 79.7 (60.9-100.4) | 81.0 (63.9-100.9) |
| 9 | Lycaenidae + *Apodemia* | 130.3 (104.3-159.4) | 131.8 (106.8-160.2) | 136.6 (113.7-162.7) | 119.0 (92.5-147.7) | 119.4 (93.5-147.4) | 122.5 (98.5-149.0) |
| 10 | *Danaus* | 26.5 (18.8-37.1) | 26.6 (19.0-36.6) | 26.6 (19.9-35.1) | 25.5 (17.7-36.4) | 25.5 (17.7-36.1) | 25.5 (18.2-35.3) |
| 11 | *Neominois + Oeneis* | 29.9 (21.1-41.5) | 29.9 (21.3-40.7) | 29.6 (21.9-39.0) | 27.9 (19.6-38.8) | 28.0 (19.4-38.7) | 28.1 (19.9-38.7) |
| 12 | *(N. ridingsii + O. chryxus) C. tullia* | 75.0 (58.6-95.0) | 75.6 (59.3-94.3) | 77.4 (62.3-95.0) | 69.5 (53.5-87.9) | 69.6 (53.9-87.7) | 70.9 (55.5-89.0) |
| 13 | *((N. ridingsii + O. chryxus) C. tullia) Bicyclus* | 87.6 (69.7-108.9) | 88.4 (70.7-109.2) | 90.7 (74.4-109.9) | 81.0 (63.2-101.0) | 81.2 (64.0-100.7) | 82.9 (66.2-102.7) |
| 14 | *Heliconius* | 20.3 (14.5-27.7) | 20.5 (14.8-27.6) | 20.8 (15.7-27.0) | 19.1 (13.4-26.1) | 19.1 (13.5-26.1) | 19.3 (14.0-25.8) |
| 15 | *Heliconius* + *Agraulis* | 37.4 (28.1-49.1) | 37.7 (28.7-48.8) | 38.7 (30.2-49.0) | 35.0 (26.1-45.8) | 35.0 (26.1-45.8) | 35.5 (27.1-45.8) |
| 16 | *((Heliconius) Agraulis) Speyeria* | 61.3 (48.1-77.5) | 61.7 (49.1-77.5) | 63.2 (51.1-77.8) | 57.1 (44.4-72.2) | 57.3 (44.9-71.9) | 58.2 (46.1-72.8) |
| 17 | *Limenitis* | 8.5 (5.6-12.6) | 8.4 (5.6-12.4) | 8.4 (5.9-11.7) | 8.1 (5.2-12.4) | 8.0 (5.2-12.2) | 8.0 (5.3-11.7) |
| 18 | *Limenitis +* node 16 | 80.6 (64.9-99.7) | 81.2 (65.6-100.0) | 83.3 (68.7-101.1) | 74.9 (59.4-92.9) | 75.1 (60.1-92.7) | 76.4 (62.0-94.0) |
| 19 | *Vanessa + Nymphalis* | 38.5 (34.2-48.7) | 38.4 (34.1-48.3) | 38.0 (34.1-46.5) | 37.4 (34.1-45.8) | 37.4 (34.1-45.8) | 37.4 (34.1-45.4) |
| 20 | *(Vanessa + Nymphalis) Euphydryas* | 70.3 (58.6-86.7) | 70.7 (58.9-86.6) | 72.0 (61.2-86.6) | 66.2 (55.3-80.9) | 66.3 (55.5-80.7) | 67.2 (56.5-81.3) |
| 21 | Node 18 + node 20 | 104.7 (85.7-127.7) | 105.8 (87.2-128.2) | 109.0 (92.0-130.0) | 96.8 (77.4-118.9) | 97.1 (78.4-118.8) | 99.3 (81.5-120.3) |
| 22 | Node 21 + node 13 | 122.1 (99.4-148.7) | 123.4 (101.2-148.7) | 127.5 (107.3-152.0) | 112.0 (88.3-138.3) | 112.4 (89.5-137.6) | 115.2 (93.8-140.1) |
| 23 | Node 22 + node 10 | 138.6 (112.6-168.2) | 140.2 (114.6-168.8) | 145.1 (121.9-172.3) | 126.3 (98.3-155.6) | 126.8 (99.7-156.3) | 130.2 (105.4-158.1) |
| 24 | Node 23 + node 9 | 153.5 (123.9-187.1) | 155.4 (126.4-187.3) | 161.2 (135.8-191.4) | 138.9 (107.3-172.1) | 139.5 (108.5-172.2) | 143.6 (115.4-174.7) |
| 25 | Node 24 + node 2 | 169.4 (136.9-206.6) | 171.3 (139.4-207.4) | 177.4 (148.9-210.7) | 151.6 (115.7-189.0) | 152.3 (117.1-189.0) | 157.0 (124.8-192.2) |
| 26 | Node 25 + node 1 | 194.0 (156.1-237.3) | 195.9 (158.7-238.3) | 201.3 (169.3-238.4) | 171.3 (129.0-214.6) | 172.3 (130.6-215.2) | 177.7 (140.3-219.0) |

Continuation Additional File 7

| Node | Node name | 100, 0. 02, 0.002 | 100, 0. 02, 0.0002 | 100, 0.002, 0.02 * | 100, 0.002, 0.002 | 100, 0.002, 0.0002 | 100, 0.0002, 0.02 |
| --- | --- | --- | --- | --- | --- | --- | --- |
| 1 | *Papilio* | 58.6 (46.9-64.8) | 59.9 (50.2-64.8) | 58.2 (45.8-64.7) | 58.7 (47.2-64.8) | 60.1 (50.6-64.8) | 59.4 (48.2-64.8) |
| 2 | *Colias + Pieris* | 93.5 (73.7-115.7) | 98.9 (80.6-119.5) | 92.4 (70.7-115.3) | 94.1 (74.0-116.7) | 99.4 (80.9-120.4) | 98.8 (76.6-122.9) |
| 3 | *Agriades + Polyommatus* | 11.7 (7.9-17.0) | 12.2 (8.7-16.5) | 11.7 (7.8-17.3) | 11.9 (8.0-17.0) | 12.3 (8.8-16.7) | 12.6 (8.4-18.6) |
| 4 | *L. helloides + L. nivalis* | 8.6 (5.6-12.6) | 9.0 (6.2-12.5) | 8.6 (5.5-12.7) | 8.7 (5.7-12.7) | 9.0 (6.2-12.6) | 9.2 (6.0-13.6) |
| 5 | *(L. helloides + L. nivalis) L. heteronea* | 14.5 (9.8-20.7) | 14.6 (10.4-20.0) | 14.5 (9.6-21.0) | 14.7 (9.9-21.0) | 14.7 (10.4-20.0) | 15.6 (10.4-22.6) |
| 6 | *((L. helloides + L. nivalis) L. heteronea) L. rubidus* | 24.5 (17.2-34.1) | 23.9 (17.5-32.1) | 24.8 (17.0-35.3) | 24.8 (17.3-34.6) | 24.1 (17.6-32.4) | 26.4 (18.2-37.9) |
| 7 | Lycaeninae + *Satyrium* | 62.7 (48.2-79.6) | 64.3 (51.0-80.0) | 62.6 (47.3-80.9) | 63.3 (48.6-80.5) | 64.8 (51.3-80.5) | 67.2 (51.1-86.8) |
| 8 | (Lycaeninae + *Satyrium)( Agriades + Polyommatus)* | 81.0 (63.9-100.9) | 84.7 (68.5-103.5) | 80.5 (61.7-101.6) | 81.8 (64.2-102.0) | 85.3 (69.0-104.2) | 86.7 (67.6-109.6) |
| 9 | Lycaenidae + *Apodemia* | 122.5 (98.5-149.0) | 130.2 (107.7-155.6) | 121.0 (94.6-149.6) | 123.6 (99.7-150.7) | 131.2 (108.7-156.8) | 131.5 (105.5-160.7) |
| 10 | *Danaus* | 25.5 (18.2-35.3) | 25.8 (19.4-34.0) | 25.6 (17.9-35.6) | 25.6 (18.1-35.5) | 25.8 (19.2-34.3) | 26.7 (18.7-37.4) |
| 11 | *Neominois + Oeneis* | 28.1 (19.9-38.7) | 28.5 (21.1-37.5) | 28.3 (19.8-39.5) | 28.3 (20.3-38.6) | 28.6 (21.3-37.6) | 30.1 (21.3-41.5) |
| 12 | *(N. ridingsii + O. chryxus) C. tullia* | 70.9 (55.5-89.0) | 74.2 (59.9-91.2) | 70.6 (54.7-89.5) | 71.5 (56.5-89.1) | 74.6 (60.3-91.4) | 75.6 (59.2-94.8) |
| 13 | *((N. ridingsii + O. chryxus) C. tullia) Bicyclus* | 82.9 (66.2-102.7) | 86.8 (71.3-104.9) | 82.4 (64.8-102.9) | 83.4 (67.0-103.1) | 87.3 (71.5-105.6) | 88.3 (70.5-109.5) |
| 14 | *Heliconius* | 19.3 (14.0-25.8) | 20.0 (15.1-26.2) | 19.2 (13.5-26.1) | 19.4 (14.1-26.2) | 20.1 (15.2-26.2) | 20.5 (14.7-27.9) |
| 15 | *Heliconius* + *Agraulis* | 35.5 (27.1-45.8) | 37.1 (29.1-46.7) | 35.3 (26.3-45.8) | 35.8 (27.2-46.4) | 37.3 (29.3-47.0) | 37.7 (28.5-49.1) |
| 16 | *((Heliconius) Agraulis) Speyeria* | 58.2 (46.1-72.8) | 60.7 (49.2-74.4) | 57.8 (45.3-72.6) | 58.6 (46.5-73.0) | 61.0 (49.6-74.9) | 61.8 (48.6-77.6) |
| 17 | *Limenitis* | 8.0 (5.3-11.7) | 8.1 (5.8-11.2) | 8.1 (5.3-12.1) | 8.1 (5.5-11.8) | 8.1 (5.7-11.2) | 8.5 (5.7-12.8) |
| 18 | *Limenitis +* node 16 | 76.4 (62.0-94.0) | 80.0 (66.2-96.7) | 75.9 (60.7-94.0) | 77.0 (62.7-94.7) | 80.3 (66.5-97.2) | 81.2 (65.5-100.2) |
| 19 | *Vanessa + Nymphalis* | 37.4 (34.1-45.4) | 37.2 (34.1-44.5) | 37.6 (34.1-45.9) | 37.5 (34.1-46.0) | 37.3 (34.1-44.7) | 38.7 (34.1-49.1) |
| 20 | *(Vanessa + Nymphalis) Euphydryas* | 67.2 (56.5-81.3) | 69.5 (59.7-83.0) | 66.9 (55.9-81.6) | 67.6 (57.0-82.1) | 69.8 (59.8-83.1) | 70.7 (58.8-87.1) |
| 21 | Node 18 + node 20 | 99.3 (81.5-120.3) | 104.5 (88.2-124.0) | 98.3 (78.9-120.3) | 99.9 (82.5-121.0) | 105.0 (88.7-124.8) | 105.5 (86.6-128.2) |
| 22 | Node 21 + node 13 | 115.2 (93.8-140.1) | 121.9 (102.7-144.5) | 113.9 (90.4-139.5) | 116.0 (94.6-140.4) | 122.6 (103.2-145.6) | 123.1 (100.2-149.4) |
| 23 | Node 22 + node 10 | 130.2 (105.4-158.1) | 138.3 (116.0-164.1) | 128.6 (101.3-158.3) | 131.3 (106.6-159.3) | 139.2 (116.7-165.0) | 139.8 (113.8-169.8) |
| 24 | Node 23 + node 9 | 143.6 (115.4-174.7) | 153.2 (128.1-181.1) | 141.6 (110.3-175.2) | 144.9 (117.0-176.3) | 154.3 (128.9-183.1) | 154.9 (125.3-188.1) |
| 25 | Node 24 + node 2 | 157.0 (124.8-192.2) | 167.9 (139.8-199.7) | 154.8 (119.7-192.0) | 158.5 (127.0-193.4) | 169.1 (141.4-200.6) | 170.9 (138.0-208.7) |
| 26 | Node 25 + node 1 | 177.7 (140.3-219.0) | 189.7 (157.8-225.0) | 175.4 (134.0-218.9) | 179.6 (142.5-219.8) | 191.2 (159.5-226.9) | 195.8 (158.1-240.3) |

Continuation Additional File 7

| Node | Node name | 100, 0. 0002, 0.002 | 100, 0. 0002, 0.0002 |
| --- | --- | --- | --- |
| 1 | *Papilio* | 59.7 (49.0-64.8) | 60.7 (51.9-64.9) |
| 2 | *Colias + Pieris* | 99.5 (78.5-123.1) | 103.4 (84.0-125.7) |
| 3 | *Agriades + Polyommatus* | 12.6 (8.7-18.2) | 12.8 (9.2-17.4) |
| 4 | *L. helloides + L. nivalis* | 9.3 (6.1-13.5) | 9.4 (6.5-13.1) |
| 5 | *(L. helloides + L. nivalis) L. heteronea* | 15.5 (10.5-22.3) | 15.3 (10.9-21.0) |
| 6 | *((L. helloides + L. nivalis) L. heteronea) L. rubidus* | 26.1 (18.3-36.9) | 25.1 (18.4-33.6) |
| 7 | Lycaeninae + *Satyrium* | 67.2 (51.9-85.6) | 67.5 (53.7-83.9) |
| 8 | (Lycaeninae + *Satyrium)( Agriades + Polyommatus)* | 87.0 (68.7-108.3) | 89.1 (72.0-108.7) |
| 9 | Lycaenidae + *Apodemia* | 132.5 (107.8-160.6) | 137.4 (113.7-164.1) |
| 10 | *Danaus* | 26.7 (19.0-37.0) | 26.7 (19.8-35.2) |
| 11 | *Neominois + Oeneis* | 30.0 (21.6-41.0) | 29.8 (22.2-39.5) |
| 12 | *(N. ridingsii + O. chryxus) C. tullia* | 75.9 (59.8-95.4) | 77.8 (63.1-95.2) |
| 13 | *((N. ridingsii + O. chryxus) C. tullia) Bicyclus* | 88.8 (71.5-110.1) | 91.0 (74.6-110.1) |
| 14 | *Heliconius* | 20.5 (15.0-27.7) | 20.9 (15.6-27.2) |
| 15 | *Heliconius* + *Agraulis* | 37.8 (28.7-49.2) | 38.8 (30.4-49.0) |
| 16 | *((Heliconius) Agraulis) Speyeria* | 61.9 (48.9-77.7) | 63.4 (51.4-77.8) |
| 17 | *Limenitis* | 8.5 (5.7-12.4) | 8.4 (5.9-11.7) |
| 18 | *Limenitis +* node 16 | 81.5 (65.7-101.1) | 83.7 (69.3-101.2) |
| 19 | *Vanessa + Nymphalis* | 38.5 (34.1-48.6) | 38.2 (34.1-46.8) |
| 20 | *(Vanessa + Nymphalis) Euphydryas* | 70.9 (59.5-87.2) | 72.2 (61.4-86.6) |
| 21 | Node 18 + node 20 | 106.2 (87.5-129.2) | 109.4 (92.5-130.4) |
| 22 | Node 21 + node 13 | 124.0 (102.1-150.2) | 128.1 (107.9-152.3) |
| 23 | Node 22 + node 10 | 140.9 (115.5-170.4) | 145.8 (122.6-172.8) |
| 24 | Node 23 + node 9 | 156.2 (127.2-188.4) | 162.1 (135.7-192.2) |
| 25 | Node 24 + node 2 | 172.3 (140.3-208.9) | 178.4 (149.5-211.7) |
| 26 | Node 25 + node 1 | 197.2 (160.3-239.3) | 202.5 (170.1-239.3) |
