# Supplementary material for: Impact of duplicate gene copies on phylogenetic analysis and divergence time estimates in butterflies
Source: BMC Evol Biol. 2009 May 13;9:99. doi: 10.1186/1471-2148-9-99 (PMC2689175; doi:10.1186/1471-2148-9-99)
Supplement: Additional file 8 — Divergence time estimates. The data in the figure show the impact of incorporating fast-evolving genes on Bayesian divergence time estimates using priors of age of ingroup node = 70 Ma, rtrate = 0.002 substitutions per site per million years and brownmean = 0.02. [file 1471-2148-9-99-S8.doc]

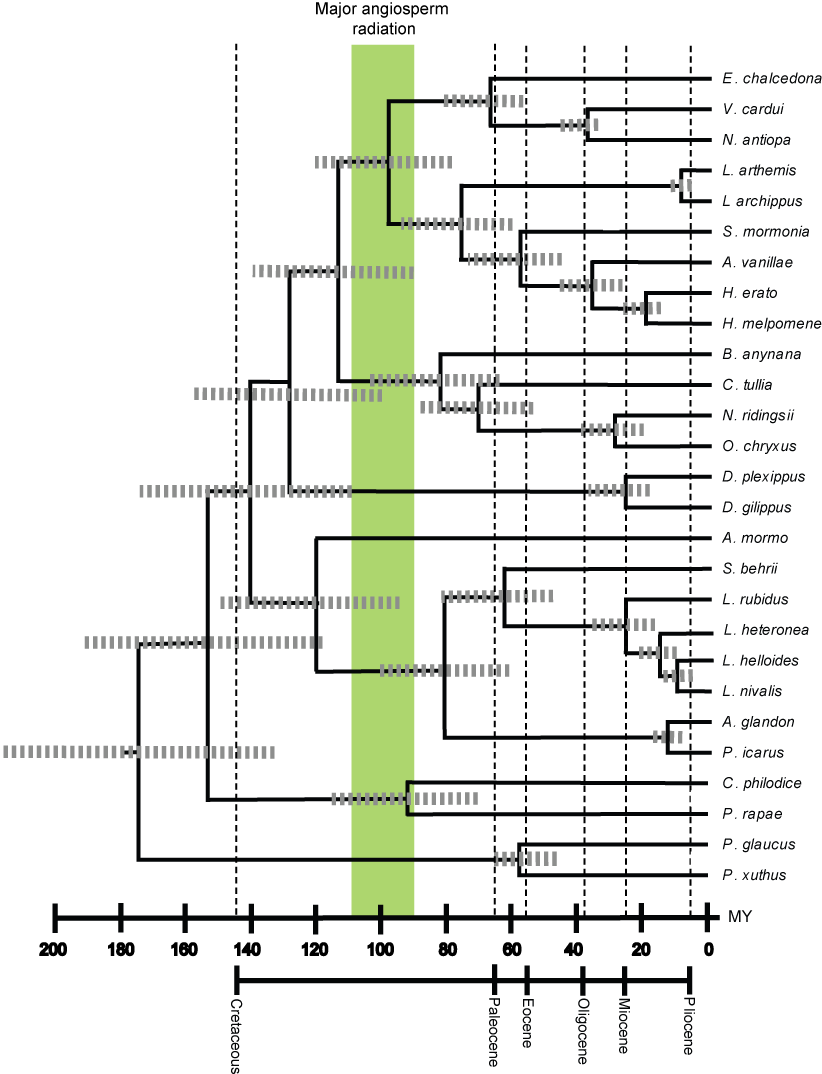


**Additional File 8.**

**Divergence time estimates.**

Divergence time estimates using the Bayesian method on the fast copy combined data set. Estimations were performed using the combined five gene data set using priors of age of ingroup node = 70 Ma, rtrate = 0.002 substitutions per site per million years and brownmean = 0.02. For each estimate 95% confidence intervals are shown. Green bar indicates the major period during which flowering plants diversified, 90-115 Mya.
