# Supplementary material for: Impact of duplicate gene copies on phylogenetic analysis and divergence time estimates in butterflies
Source: BMC Evol Biol. 2009 May 13;9:99. doi: 10.1186/1471-2148-9-99 (PMC2689175; doi:10.1186/1471-2148-9-99)
Supplement: Additional file 9 — Primers used in the study. The sequences represent primers used in 5'RACE of opsin genes. [file 1471-2148-9-99-S9.doc]

**Additional File 9.** Primers used in 5’RACE of opsin cDNAs.

| **Species** | ***UVRh*** | ***BRh*** | ***LWRh*** |
| --- | --- | --- | --- |
| *E. chalcedona* | AGGATCGATACACGCTACAGTCTT | CACAGACGAACAGGAAGAAGATTG | AGTAGCTAGAGGCGTGAGTGTGAT |
| *N. antiopa* | AAAGCTCCTATTAACGACATCACG |  | GCAGTTTCGAAG ATACCAGCATAG |
| *L. arthemis* | CAAGAAGCAACGAAGAGGAAACAC |  | N/A |
| *L. archippus* | GGCGGGTCGACGCTGATGCTGCTGTTGCAGAAGA | GGCGGGTCGACGCTTCTGCGGCTGCGTGTTGC | N/A |
| *D. gilippus* | AACGACGAACAATGCGAAACC | CATTCAACCTTACTACCATCACAA | TTCTGCCAATGCGATGTTCTTTAT |
| *H. melpomene* | ACGCAACAAAGAGGAAGCACAC |  | GTTTTGAAGATTCCGGCATAGTTG |
| *A. vanillae* | ATGGCATGCGTCGTTGTAGTT | TCTGGAGTTCCGCCCTGTATCTA | GGTCACAATAGGGCTAATCGTCAT |
| *S. mormonia* | GACGCAACAAAGAGGAAGCACAC | ATGGATCAATGCAGGAGACGAC | TTCGAAGATTCCGGCATAGTTGAT |
| *C. tullia* | CTCTTGCCTGTACTTGGGATGACT | CCTGTCACCAAAAGCTCCTATCAT | GATGGCGCTCGTCTATGTGTC |
| *N. ridingsii* | TCGCTGAAGTTCTTGTCTGTATTT | N/A | TCGAAGATGCCGGAGTAGTTGA |
| *O. chryxus* | GGAATCATTGTTACGCCTGGTGT | N/A | GAAGATGCCGGA GTAGTTGATGAC |
| *L. helloides* | GGTGTCCAGGCGCAGATGAAGAGG | RTCCTYTATCCGGTTGTCGTTCGT  GGTGTCCAGGCGCAGATGAAGAGG | GTCCAAGCACGGTCGGTCAGTT |
| *L. heteronea* | AATGTAGATGTAGAAATGCTTGCA | GTGACGATGACTGGAGCCTTC  GGTGTCCAGGCGCAGATGAAGAGG | CATCTGGGGTTCAATCTTCGC |
| *L. nivalis* | GAGAGGCCGTGTTATTGTTGAGTG | RTCCTYTATCCGGTTGTCGTTCGT  TTCACGGATTCGATGTAACTTTAG | GTCCAAGCACGGTCGGTCAGTT |
| *S. behrii* | CTACAGCCTTACAAGCCACAGC |  | ACGCCATGAACCAGAGTGAGAT |
| *A. glandon* | AGGTGTCAACAAGTTCTGGTCTCC |  | CTCGGTACTTAGGATGGCTTATGC |
| *A. mormo* | GAAGAAACGAACAGGAAACAAACT |  | AAGTGGAGTGATTTTCGCTGTCT  AGATGGTTGACAGTGGAGTTAGAC |
| *C. philodice* | AGCGACAAATAGGAAACAGACAGT |  | CGAATACACCAGCGAAGTTGAT |
